# Positive and Negative Parenting Practices and Offspring Disruptive Behavior: A Meta-Analytic Review of Quasi-Experimental Evidence

**DOI:** 10.1037/bul0000495

**Published:** 2025-11

**Authors:** Lucy Karwatowska, Francesca Solmi, Jessie R. Baldwin, Sara R. Jaffee, Essi Viding, Jean-Baptiste Pingault, Bianca Lucia De Stavola

**Affiliations:** 1 Great Ormond Street Institute of Child Health, University College London; 2 Division of Psychiatry, University College London; 3 Department of Clinical, Educational and Health Psychology, University College London; 4 Department of Psychology, University of Pennsylvania; 5 Social, Genetic and Developmental Psychiatry Centre, King’s College London

**Keywords:** disruptive behavior, causal inference, meta-analysis, parenting, quasi-experimental

## Abstract

Disruptive behavior disorders (DBDs) are common in childhood and adolescence, with global estimates of 5.7%. While parenting practices are associated with DBDs, it is not clear whether these associations reflect causal effects or confounding. To strengthen causal inference, we meta-analyzed quasi-experimental evidence on the relationship between parenting practices and DBD symptoms. We conducted multilevel random-effects meta-analyses to pool results and assess evidence of heterogeneity and moderator analyses to further investigate potential sources of heterogeneity. We identified 45 studies that used data from 28 distinct cohorts (*n* = 38,591) and implemented seven different quasi-experimental methods. There was evidence of a causal effect of negative parenting practices on offspring DBD symptoms (Pearson’s *r* = 0.13; 95% confidence interval, CI [0.09, 0.16]; 95% prediction interval, PI [−0.08, 0.35]; *n* = 30,677), but no effect of positive parenting practices (*r* = −0.06; 95% CI [−0.14, 0.02]; 95% PI [−0.39, 0.28]; *n* = 21,100). Moderator analyses indicated that the effect of negative parenting was consistent across offspring characteristics and maternal and paternal parenting but varied by type of quasi-experimental method, informant for the exposure and outcome, and study quality. The present study thus provides evidence of a small, harmful, causal effect of negative parenting practices on offspring DBDs. Effectively targeting such parenting practices could reduce the substantial societal burden of DBDs, with a potential 4% decrease in the global prevalence of DBD symptoms. This is equivalent to approximately 4.5 million school-aged children no longer meeting clinical thresholds for DBDs, which may reduce pressure on the criminal justice, health care, and social welfare sectors.

## Public Significance Statement

Prevention programs that effectively target negative parenting practices, such as harsh and inconsistent discipline, could reduce the prevalence of disruptive behavior disorders by 4% worldwide, possibly preventing 4.5 million school-aged children from exhibiting clinical symptoms of disruptive behavior. Given the substantial financial and social costs of disruptive behavior, as well as the demand they place on services, even small shifts at the population level could yield large societal impacts. Furthermore, the impact of successful prevention programs could be exponential across generations, as more children are exposed to models of parenting that do not rely on negative practices.

Disruptive behavior disorders (DBDs), which include conduct disorder (CD) and oppositional defiant disorder (ODD), are characterized by symptoms such as physical aggression, lying, stealing, and frequently losing one’s temper. When persistent, DBDs are associated with increased mental and physical health problems and poorer educational and social outcomes (Fairchild et al., 2019). In addition to these individual-level consequences, DBDs impose a substantial financial burden on society, including costs related to health care, education, social services, and the criminal justice system (Rissanen et al., 2022; Rivenbark et al., 2018). Globally, an estimated 3.6% of children meet criteria for ODD, 2.1% for CD, and thus 5.7% for any DBD (Polanczyk et al., 2015). Given the significant individual and societal burden of DBDs, it is crucial to understand their underlying causes, which involve a complex interplay of genetic and environmental risk factors (Fairchild et al., 2019).

Caregivers play a key role in child development, and the most extensively studied and widely used interventions for DBDs focus on modifying parenting practices (e.g., Eyberg et al., 2008; Pilling et al., 2013). These interventions are based on the assumption that parenting influences offspring’s disruptive behavior through *social learning* (i.e., offspring learn disruptive behaviors by observing and imitating their parents; Bandura & Walters, 1963) and *operant conditioning* (i.e., parents modify their offspring’s disruptive behavior through the use of rewards and punishments; Patterson, 1982). Parent management training, an umbrella term to describe prevention and intervention programs that target parenting practices, aims to improve the parent–child relationship by reducing negative parenting practices (e.g., harsh, coercive parenting) and promoting positive parenting practices (e.g., warm, consistent, and supportive parenting), alongside encouraging greater parental involvement and communication between parents and their offspring. Experimental studies (i.e., randomized control trials [RCTs]) have shown that parent management training reduces child disruptive behavior, with effect sizes that are moderate in magnitude, but highly heterogeneous (Cohen’s *d* = −0.21 to −0.69; Leijten et al., 2019).

Although RCTs are considered the “gold standard” for establishing the causal effect of interventions, they do not identify which aspect(s) of parenting interventions lead to reductions in disruptive behavior (Leijten et al., 2019). Parenting interventions target a suite of positive and negative parenting practices, only some or all of which could be central for the effectiveness of interventions (Leijten et al., 2022). Quasi-experimental studies, which estimate population-level causal effects using observational data, can be used to examine the association between *specific* parenting practices (e.g., harsh discipline, coercion) and disruptive behaviors.

Quasi-experimental studies operate within the counterfactual framework, comparing hypothetical scenarios in which the same individual is exposed and unexposed to a risk factor. The causal effect of the exposure is defined as the difference between the average outcome in each scenario. The estimation of these causal effects is achieved either by adopting (a) methods that rely on an instrument (e.g., regression discontinuity, Mendelian randomization, difference-in-difference approaches) or (b) confounder-control methods (e.g., extensions to regression-based methods, propensity score matching). RCTs and quasi-experimental studies ask different but complementary questions that can be used to triangulate our understanding of the influence of parenting practices on disruptive behavior.

Quasi-experimental studies can also address some of the challenges and limitations associated with RCTs. RCTs are expensive to run and, as a result, often have a short follow-up duration, difficulty recruiting a diverse range of individuals, and sometimes suffer from different levels of attrition by intervention arm, resulting in low external validity (Bärnighausen, Røttingen, et al., 2017; Hernán & Robins, 2016). In comparison, quasi-experimental studies have the potential for higher external validity as they often use data from large, representative samples with long-term follow-up (Bärnighausen, Tugwell, et al., 2017).

This review aims to provide a quantitative synthesis of findings from quasi-experimental studies of parenting and DBDs. To the best of our knowledge, only one narrative review to date has synthesized evidence on the relationship between harsh parental discipline, maltreatment, and antisocial behavior (Jaffee et al., 2012). In their review, Jaffee and colleagues concluded that there was evidence that negative parenting practices had causal effects on antisocial behavior. They also reported evidence of reverse causation (i.e., parent- *and* child-driven effects) and familial confounding, indicating that genes shared by parents and children or other aspects of the familial environment could account for observed associations between maltreatment or harsh discipline and antisocial behavior.

The studies included in this previous review were not quantitatively synthesized, and since its publication, there has been a substantial increase in the number of studies using quasi-experimental methods to investigate this topic. Thus, a quantitative synthesis of the results from quasi-experimental studies is both timely and important. The results will complement existing quantitative reviews of experimental (Mingebach et al., 2018) and observational studies (Pinquart, 2017; Rothbaum & Weisz, 1994) on parenting practices and DBDs. We will also examine whether any reported causal effects vary by offspring characteristics and/or study features, namely:

1 *Offspring sex:* The prevalence of DBDs is higher in boys than in girls (Polanczyk et al., 2015), which could indicate that boys either experience a greater number of risk factors for DBDs than girls or that these risk factors have a greater impact on DBD in boys than in girls (Moffitt et al., 2001). Thus, we will examine whether the effects of parenting on disruptive behavior varied according to offspring sex.
2 *Offspring age:* There may be “sensitive periods” during childhood or adolescence when parenting practices are more or less influential on DBDs (Scott et al., 2018; Wachs et al., 2014). Previous findings are mixed: some studies report larger effects in early childhood, others find larger effects in later childhood and adolescence, while some find a consistent effect across developmental stages (Gardner et al., 2019; Jeong et al., 2021; Pinquart, 2017; Rodrigues et al., 2021). We will explore this further by considering whether our results varied by offspring age at assessment(s).
3 *Type of DBD outcome:* It has been suggested that parenting practices may have a greater influence on broader measures of disruptive behavior (e.g., multiple symptoms) compared to narrower, symptom-specific measures (e.g., aggression; Pinquart, 2017). Consequently, we will explore whether our results differed according to the type of DBD outcome.
4 *Type of quasi-experimental method:* Different types of quasi-experimental methods address different sources of confounding (Goetghebeur et al., 2020; Pingault et al., 2022). As such, we will examine whether the type of quasi-experimental method used influences the magnitude of the reported effects.
5 *Time between exposure and outcome assessments:* Previous meta-analyses of parenting interventions suggest that the effects on disruptive behavior can remain significant at follow-up (Mingebach et al., 2018). Therefore, we will assess whether the time between exposure and outcome assessments moderates the effect of parenting practices on DBD symptoms.
6 *Informant for the exposure and outcome:* Shared method variance, a bias that can arise when the same informant reports both the exposure and outcome, can inflate associations between parenting practices and disruptive behavior (De Los Reyes et al., 2009). To assess the potential impact of this bias, we will compare the results from studies where the same informant reported both the exposure and outcome with those using different informants.
7 *Study quality:* Given that variations in study characteristics can influence findings (Lipsey & Wilson, 2001), we will evaluate whether our results differ according to the study’s risk of bias rating.
8 *Maternal or paternal parenting:* Although much less studied, paternal parenting is thought to have similar effects on offspring DBD symptoms to maternal parenting (Jeong et al., 2016). To add to the literature, we will examine whether the impact of parenting practices on DBD symptoms differs for mothers versus fathers.

## Method

### Transparency and Openness

The protocol for the present review was preregistered with the International Prospective Register of Systematic Reviews database (CRD42020169313; Karwatowska et al., 2024) and published in a peer-reviewed journal (Karwatowska et al., 2020). We adhered to the Preferred Reporting Items for Systematic Reviews and Meta-Analyses (Shamseer et al., 2015; Supplemental Table S1) statement and Meta-Analyses of Observational Studies in Epidemiology (Stroup et al., 2000; Supplemental Table S2) reporting guidelines. The data and scripts are available on GitHub (Karwatowska, 2025).

### Study Selection

Studies meeting all of the following criteria were included in the review:

• Only included human participants.
• Included at least one clearly defined measure of parenting practices and at least one clearly defined measure of disruptive behavior.
• Included a measure of parenting that was assessed either before or concurrently with the outcome.
• Published in English, although the study could have been conducted in any country.
• Published after January 1980.
• Used a quasi-experimental method (see definitions below).

Studies meeting any of the following criteria were excluded:

• The study was a case report, clinical trial, systematic review, meta-analysis, or thesis.
• The study used populations selected on physical health problems (e.g., cancer, seizures, surgery, low gestational age).
• The study used populations selected on other diagnosed developmental disorders (e.g., language disorders, learning disorders, motor disorders, autism spectrum disorders) or mental health diagnoses (e.g., schizophrenia, depression, bipolar).

### Measures

Further definitions of the key terms used in the review are given in the Supplemental Tables S3–S5.

#### Quasi-Experimental Studies

We broadly defined quasi-experimental studies as those that use observational data (in contrast to RCTs) to estimate population-level causal effects either by adopting (a) methods that rely on an instrument (e.g., regression discontinuity, Mendelian randomization, difference-in-difference approaches) or (b) confounder-control methods (e.g., extensions to regression-based methods, propensity score matching). Definitions and potential limitations of the different quasi-experimental studies included in this review are available in the Supplemental Table S3.

#### Positive and Negative Parenting Practices

We defined *positive parenting practices* as being warm, sensitive, or child-centered (e.g., use of praise or interest in offspring’s hobbies) and *negative parenting practices* as being harsh or insensitive (e.g., shouting, threatening behavior). We did not include physical discipline, abuse, or violence, as these do not represent “normative” parenting practices. We treated positive and negative parenting practices as separate constructs, as they are thought to have unique influences on offspring disruptive behavior (Hipwell et al., 2008; Oliver et al., 2014; Pettit et al., 1997).

#### DBD Symptoms

We defined the outcome either by symptoms (e.g., conduct problems [CP], externalizing problems) or clinical diagnoses (e.g., CD, ODD, psychopathy, antisocial personality disorder) associated with disruptive behavior, which we refer to broadly as DBD symptoms. Further definitions are available in the Supplemental Table S5.

### Search Strategy

We searched Embase, APA PsycInfo, and MEDLINE for peer-reviewed studies written in English and published from January 1980 to April 2024. Search terms are reported in full in the Supplemental Table S6 and included terms relating to DBDs, parenting practices, and quasi-experimental methods. Two authors (LK and FS) independently screened the titles and abstracts of all articles retrieved from the searches. The full texts of all potentially eligible studies were also reviewed by two authors (LK and FS or BLDS).

### Data Extraction

After the full-text screen, two authors (LK and JRB) independently extracted data from all eligible studies, including information on sample size, confounder adjustment, and effect sizes. The original study authors were contacted when this information was either missing or incomplete. When multiple effect sizes were available, the most conservative estimate (i.e., with the greatest degree of control for confounding) was extracted.

### Risk of Bias

We adapted the Newcastle–Ottawa scale (Wells et al., 2000) to include questions relevant to quasi-experimental studies. Additional/adapted questions included control for environmental and genetic confounders (Supplemental Table S7, Questions 5 and 6), whether the exposure and outcome were reported by different informants (Question 8), and whether the exposure and outcome were assessed longitudinally (Question 9). An overall score was derived by summing the scores across all items (highest possible score = 10), and the 33rd and 66th percentiles were used to categorize the studies into one of three categories used in the original Newcastle–Ottawa scale: “very high risk of bias” (score below 5.5), “high risk of bias” (score between 5.5 and 7), or “high quality” (score above 7). For studies that reported multiple effect estimates in different categories (e.g., high quality and high risk of bias), we gave the study an overall rating that corresponded to the highest category (e.g., high quality). One author (LK) coded study quality, and any questions were discussed with two members of the team (BLDS and J-BP).

### Effect Size Transformation, Interpretation, and Significance

Most studies measured parenting practices and DBD symptoms on a continuous scale. If the effect parameters were not already standardized (i.e., reported as [Pearson’s correlations] *r*), these were transformed into Pearson’s correlations using the formulae reported in Supplemental Table S8. Therefore, the results from the meta-analyses represent the association between a 1 *SD* difference in a standardized parenting practices score and corresponding changes in a standardized offspring DBD score.

For negative parenting practices measures, a positive effect size (*r*) indicates that higher levels of negative parenting (e.g., more harsh or inconsistent discipline) are associated with more DBD symptoms. A negative effect size suggests that negative parenting is associated with lower levels of DBD symptoms. For positive parenting practices measures, a positive effect size indicates that higher levels of positive parenting (e.g., more warm and affectionate parenting practices) are associated with more DBD symptoms, while a negative effect size suggests an association with lower levels of DBD symptoms.

If standard errors of the reported parameters were not available, they were calculated using the sample sizes and reported *p* values.

### Multilevel Random-Effects Model

All analyses were conducted in R (4.1.0) using the *metafor* (Version 4.3-7; Viechtbauer, 2010) package. As most studies (*k* = 36; 80%) reported estimates for multiple measures of parenting practices and many studies (*k* = 28; 62%) used data from the same data sources (i.e., the same cohort), we fitted three-level linear random-effects models (Assink & Wibbelink, 2016) with the reported effect estimate nested within study nested within cohorts (see Supplemental Figure S1), which resulted in an overall “pooled” *r*.

To evaluate possible publication bias, we created funnel plots to check for asymmetry in the distribution of estimates according to their precision and conducted various additional analyses, including Egger’s test of heterogeneity (Rodgers & Pustejovsky, 2021) and leave-one-out analyses to recalculate the Egger’s test when certain effect estimates were excluded (Viechtbauer & Cheung, 2010).

We also examined potential heterogeneity using the Cochrane *Q*, *I*^2^, and τ^2^ statistics. We interpreted an *I*^2^ of more than 50% as an indication of moderate heterogeneity (Higgins et al., 2019). To further investigate possible sources of heterogeneity, we conducted another set of leave-one-out analyses where we recalculated the *Q*, *I*^2^, and τ^2^ statistics to see if statistical inferences changed when certain effect estimates were excluded.

### Moderator Analyses

We performed moderator analyses using a priori factors to check whether the pooled estimates differed according to participant characteristics and/or study features. The significance of between-group heterogeneity was assessed by the Wald test. Pooled estimates were calculated only if there were at least three effect sizes in each category/level and if there was no strong evidence of between-study heterogeneity.

### Population Attributable Impact

We estimated the impact of intervening on parenting, which we call the “population attributable impact,” as the number of cases of DBDs that could be prevented if an effective parenting intervention were available. Assuming the population prevalence of clinically relevant symptoms of DBDs is 5.7% (Polanczyk et al., 2015), and this corresponds to the top 2.5% “tail” of the distribution of a standardized DBD score (normally distributed with a mean = 0 and standard deviation [*SD*] = 1), we derived the DBD score value above which a diagnosis would be recorded (denoted by *z*). We then used our estimated pooled meta-analytic effect (assuming causality) to estimate the change in the mean DBD score (*z*) resulting from a 0.33 *SD* change in parenting practices, which corresponds to the effect size observed in parenting interventions (see Jeong et al., 2021). We then recalculated the area of the tail that would be greater than our new *z*. The difference between the two *z* values was interpreted as the number of individuals that would have previously exhibited clinical levels of DBD symptoms who would no longer reach the clinical threshold after the hypothetical intervention (see Supplemental Figure S2 for a visualization).

### Protocol Deviations

Although the original protocol outlined a meta-analysis of *all* risk factors for DBDs (Karwatowska et al., 2020, 2024), we identified a large number of eligible studies (number of studies [*k*] = 181) in our initial searches. As a result, we chose to focus this review on a subset of studies specifically examining parenting practices. Additionally, we conducted post hoc analyses on one potential moderator, maternal versus paternal parenting practices, as this was relevant to the exposure considered in this article and not to the broader meta-analysis on all risk factors. These decisions represent deviations from the original study protocol.

## Results

### Search Results

The study selection procedures are summarized in Supplemental Figure S3. Details of studies following full-text screening, along with the rationale for exclusion, are presented in Supplemental Table S9. We identified 45 studies that examined data from 28 distinct cohorts. The total analytic sample was 38,591 individuals (48.1% female), the mean age at which parenting practices were assessed was 10.37 years, and the mean age at which DBD symptoms were assessed was 11.62 years. Further information on the included studies appears in Table 1.[Table tbl1]

### Descriptive Analyses

The 45 studies included in the meta-analyses assessed nine types of positive parenting practices and 14 types of negative parenting practices (Table 2). They used a total of seven different quasi-experimental methods, including the adoption design, discordant monozygotic twin design, discordant sibling design, extended children of twins design, in-vitro fertilization design, propensity score matching analyses, and within-person fixed effects analyses (Table 3).[Table tbl2][Table tbl3]

From the 45 studies, we extracted 155 adjusted effect sizes (number of individuals [*n*] = 38,591), including 35 effect sizes for positive parenting measures (*k* = 17; *n* = 21,100; Supplemental Table S9) and 120 effect sizes for negative parenting measures (*k* = 38; *n* = 35,201; Supplemental Table S10). Across all studies, most of the parenting measures focused on maternal parenting practices. While 13 studies (28.9%) assessed maternal parenting practices only, none solely assessed paternal parenting practices. Fourteen studies (31.1%) included separate and 18 studies (40.0%) combined measures of maternal and paternal behavior.

In terms of offspring characteristics, most studies (*k* = 38; 69.1%) included mixed-sex samples. Four studies (7.3%) included male-only samples, and three (5.5%) included female-only samples. In almost half of the studies (*k* = 22; 48.9%), the majority ancestry was White; in four studies (8.9%), it was Asian; in one study (2.2%), it was Hispanic; and in one study (2.2%), it was African American. Ancestry was not reported in two out of five studies (*k* = 17; 37.8%).

Most studies (*k* = 34; 73.8%) were longitudinal with repeated measures available on participants over time. One in five studies (*k* = 9; 20.0%) did not account for any covariates. Of the studies that adjusted for at least one covariate, the most common were offspring sex (*k* = 21; 20.0%) and offspring age (*k* = 18; 17.1%).

### Main Meta-Analytic Results

**Figure 1 fig1:**
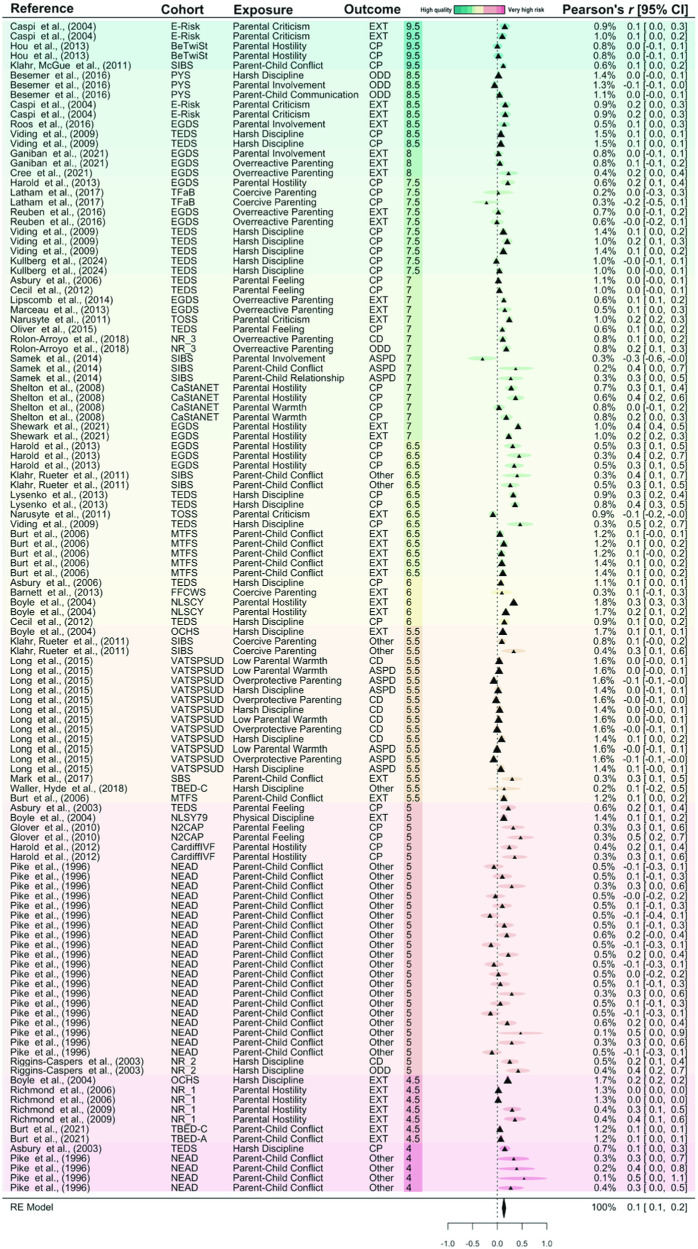
Forest Plot of the Effect of Negative Parenting Practices on Offspring Disruptive Behavior Disorder Symptoms *Note*. Results are ordered by risk of bias score, with the color scheme indicating study quality: green representing studies scoring higher on the adapted Newcastle–Ottawa scale (“high quality”) and red representing studies scoring lower (“very high risk of bias”). The percentages shown in the column to the left of Pearson’s *r* [95% CIs] represent the weights assigned to each estimate in the meta-analysis. BeTwiSt = Beijing Twin Study; CaStANET = Cardiff Study of All Wales and North West of England Twins; CardiffIVF = Cardiff in-vitro fertilization Study; E-Risk = Environmental Risk Longitudinal Twin Study; EGDS = Early Growth and Development Study; FFCWS = Fragile Families and Child Wellbeing Study; MTFS = Minnesota Twin Family Study; N2CAP = Northeast–Northwest Collaborative Adoption Projects; NEAD = Nonshared Environment and Adolescent Development project; NLSCY = National Longitudinal Survey of Children and Youth; NLSY79 = National Longitudinal Study of Youth; NR_1 = Not Reported 1; NR_2 = Not Reported 2; NR_3 = Not Reported 3; OCHS = Ontario Child Health Study; PYS = Pittsburgh Youth Study; SBS = Sisters and Brothers Study; SIBS = Sibling Interaction and Behavior Study; TBED-A = Twin Study of Behavioral and Emotional Development in Adolescents; TBED-C = Twin Study of Behavioral and Emotional Development in Children; TEDS = Twins Early Development Study; TFaB = The Twins, Family and Behavior; TOSS = Twin and Offspring Study in Sweden and from the Twin Study of Child and Adolescent Development both from the Swedish Twin Registry; VATSPSUD = Virginia Adult Twin Study of Psychiatric and Substance Use Disorders; Outcomes: ASPD = antisocial personality disorder; CP = conduct problems; EXT = externalizing symptoms; ODD = oppositional defiant disorder; *r* = Pearson’s *r* correlation; 95% CI = 95% confidence intervals; RE = random effects. See the online article for the color version of this figure.

**Figure 2 fig2:**
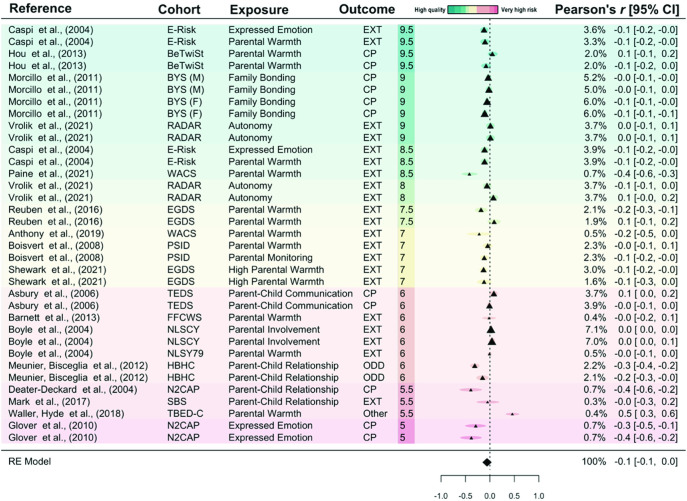
Forest Plot of the Effect of Positive Parenting Practices on Offspring Disruptive Behavior Disorder Symptoms *Note*. Results are ordered by risk of bias score, with the color scheme indicating study quality: green representing studies scoring higher on the adapted Newcastle–Ottawa scale (“high quality”) and red representing studies scoring lower (“very high risk of bias”). The percentages shown in the column to the left of Pearson’s *r* [95% CIs] represent the weights assigned to each estimate in the meta-analysis. BYS (F) = Boricua Youth Study—Females; BYS (M) = Boricua Youth Study—Males; BeTwiSt = Beijing Twin Study; E-Risk = Environmental Risk Longitudinal Twin Study; EGDS = Early Growth and Development Study; FFCWS = Fragile Families and Child Wellbeing Study; HBHC = Healthy Babies Healthy Children; N2CAP = Northeast–Northwest Collaborative Adoption Projects; NLSCY = National Longitudinal Survey of Children and Youth; NLSY79 = National Longitudinal Study of Youth; PSID = Panel Study of Income Dynamics—Child Development Supplement; RADAR = Research on Adolescent Development and Relationships; SBS = Sisters and Brothers Study; TBED-C = Twin Study of Behavioral and Emotional Development in Children; TEDS = Twins Early Development Study; WACS = Wales Adoption Cohort Study; ASPD = antisocial personality disorder; CP = conduct problems; EXT = externalizing symptoms; ODD = oppositional defiant disorder; *r* = Pearson’s *r* correlation; 95% CI = 95% confidence intervals; RE = random effects. See the online article for the color version of this figure.

The multilevel random-effects model for negative parenting found a moderate effect on offspring DBD symptoms (pooled Pearson’s *r* = 0.13; 95% confidence interval, CI [0.10, 0.17]; 95% prediction interval, PI [−0.08, 0.35]; *n* = 35,201). The results suggest that an increase in negative parenting practices is associated with an increase in offspring DBD symptoms. There was low effect heterogeneity (*I*^2^ = 21.19; τ^2^ < .0001). As shown in Figure 1 and the moderator analyses (below), the reported estimates seem to vary by study quality (green to red = high quality to very high risk of bias; see Supplemental Table S11 for a descriptive summary of the studies by risk of bias category). The association between negative parenting practices and offspring DBD symptoms was more consistent in the high-quality studies (those coded in green) than in studies with high risk of bias (those coded in red). The meta-analysis of positive parenting practices found no association with DBD symptoms and greater variability across studies (*r* = −0.06; 95% CI [−0.14, 0.02]; 95% PI [−0.40, 0.28]; *n* = 21,100; *I*^2^ = 42.20%; τ^2^ = .0264; Figure 2).[Fig fig1][Fig fig2]

### Sensitivity Analyses

#### Publication Bias

To assess publication bias (i.e., whether the studies included in the meta-analyses that had smaller sample sizes preferentially reported estimates in the expected direction), we visually examined funnel plots and conducted Egger’s test for asymmetry. Publication bias is suspected if the funnel plots are asymmetrical, supported by a *p* value of the Egger’s test below the significance threshold of .05. For positive parenting measures, there was no indication of publication bias (*p* = .583; Supplemental Figure S4A). The funnel plot for negative parenting was asymmetrical, and Egger’s test was significant (*p* < .001; Supplemental Figure S4B). While this may suggest the presence of publication bias, the results from Egger’s test should be interpreted with caution as significant results can arise from other confounding factors.

To understand whether publication bias was driven by any particular studies, effect sizes, or study features, we reran Egger’s test for heterogeneity after removing each effect size, study, cohort, quasi-experimental type, or risk of bias category from the analyses in turn. We then compared the results from these Egger’s tests to the original results to assess whether the *p* value became larger, which would suggest that publication bias was weakened when those selected effect sizes were left out. The resulting *p* values suggested that no individual effect size, study, or cohort was driving the publication bias in the studies reporting negative parenting measures (see Supplemental Figures S5–S7). Publication bias was slightly weakened when the studies categorized as very high risk of bias were removed (*k* = 11; number of effect sizes [ES] = 54; Supplemental Figure S8A), but not when those categorized as high risk of bias (*k* = 5; ES = 54; Supplemental Figure S8B) or high quality (*k* = 22; ES = 50; Supplemental Figure S8C) were removed.

#### Leave-One-Out Analyses

Leave-one-out analyses indicated that the overall pooled estimate for negative parenting was not unduly influenced by individual effect sizes, studies, or cohorts. The meta-analytic effect size (*r*) ranged from 0.13 to 0.14 after omitting each of the 120 effect sizes, 38 studies, and 23 cohorts in turn (see Supplemental Figures S5–S7).

### Meta-Analysis Results for the Highest Quality Studies

As there was evidence of publication bias in very high-risk-of-bias studies, we excluded effect estimates from these studies and reran our meta-analysis. This produced our most conservative pooled estimate (*r* = 0.13; 95% CI [0.09, 0.17]; 95% PI [−0.09, 0.34]; *k* = 27; ES = 68; *n* = 30,677).

### Moderator Analyses

To identify potential sources of heterogeneity in the association between parenting practices and offspring disruptive behavior, we ran moderator analyses defined by a set of prespecified variables, including participant (e.g., sex, age at outcome) and study features (e.g., type of DBD outcome, type of quasi-experimental method used, time between exposure and outcome assessment, whether the exposure and outcomes were reported by the same informant, data quality, and maternal vs. paternal parenting; see Table 4). The moderator analyses were only run for negative parenting measures as the meta-analytic results for positive parenting were not significant, and there were an insufficient number of estimates available for moderator analyses (see Supplemental Figures S10–S13).[Table tbl4]

In terms of participant characteristics, the results suggested that the association between negative parenting practices and DBD symptoms did not differ depending on the percentage of females in the sample nor offspring age at either exposure or outcome assessment, after controlling for time between assessments (all nonsignificant, *p*_moderator_ > .05; Table 4).

Regarding study features, the results suggested that the effect of negative parenting practices on offspring disruptive behavior was similar regardless of the DBD outcome, including CP, CD, and antisocial personality disorder; the amount of time between exposure and the outcome assessments; or whether mother’s or father’s parenting was assessed (all nonsignificant, *p*_moderator_ > .05; Table 4).

Although we were not able to include all quasi-experimental methods in the moderator analyses due to small numbers of effect sizes, there was evidence that the magnitude of the effect differed depending on the quasi-experimental method used in the study (*p*_moderator_ = .027; Supplemental Figure S10). Further analyses suggested that adoption studies (*r* = 0.19; 95% CI [0.12, 0.25]; *k* = 13; ES = 26; *n* = 1,468) reported the largest effects, followed by discordant sibling studies (*r* = 0.17; 95% CI [0.09, 0.26]; *k* = 7; ES = 15; *n* = 22,362), discordant twin studies (*r* = 0.08; 95% CI [0.05, 0.12]; *k* = 14; ES = 70; *n* = 13,271), and finally within-person fixed effect studies (*r* = 0.07; 95% CI [−0.06, 0.20]; *k* = 2; ES = 5; *n* = 661).

There was also evidence that the association between negative parenting practices and offspring disruptive behavior was influenced by whether the exposure and outcome were rated by the same informant (*p*_moderator_ < .001; Supplemental Figure S11). The pooled effect was smaller when the exposure and outcome were rated by different people (*r* = 0.09; 95% CI [0.04, 0.13]; *k* = 21; ES = 54; *n* = 14,303) compared to when exposure and outcome were reported by the same informant (*r* = 0.17; 95% CI [0.13, 0.22]; *k* = 23; ES = 65; *n* = 30,280).

The analyses of study quality suggested that the degree of risk of bias in a study was associated with the pooled estimates for negative parenting (*p*_moderator_ = .024; Supplemental Figure S12). Studies judged to be very high risk or high risk of bias reported the largest effects (*r* = 0.15; 95% CI [0.09, 0.22]; *k* = 11; ES = 52; *n* = 8,792 and *r* = 0.19; 95% CI [0.10, 0.29]; *k* = 5; ES = 18; *n* = 23,063, respectively), and studies judged to be high quality reported the smallest effects (*r* = 0.11; 95% CI [0.06, 0.15]; *k* = 22; ES = 50; *n* = 14,318).

### Calculation of the Population Attributable Impact of Negative Parenting Practices

To estimate the impact of intervening on negative parenting, we calculated the “population attributable impact” of negative parenting, that is, the number of individuals that might no longer exhibit clinically relevant DBD symptoms if the mean score of negative parenting could be reduced. Using our most conservative estimate (i.e., excluding studies judged to be very high risk of bias; *r* = 0.126), we calculated that an effective hypothetical intervention would lead to a 4% reduction in the prevalence of clinically relevant DBD symptoms worldwide, the equivalent of approximately 4.5 million school-aged children worldwide no longer exhibiting clinical levels of DBD symptoms (see Supplemental Figure S1).

## Discussion

This study is the first to quantitatively synthesize quasi-experimental evidence on the effect of parenting practices on offspring DBD symptoms. To be included, studies had to have used a quasi-experimental method, that is, methods that can estimate population-level causal effects from observational data by either (a) using an instrument or (b) applying confounder-control techniques. The analyses included 45 studies using data from 28 distinct cohorts with a total of 38,591 participants.

The findings suggest that negative, but not positive, parenting practices have a small causal effect on offspring DBD symptoms. Our most conservative meta-analytic effect indicated that a one-unit increase in a standardized negative parenting practices score (e.g., equivalent to an increase of 0.58 points on the negativity subscale of the Iowa Family Interaction Rating Scales; Williamson et al., 2011) was associated with a 0.13 *SD* increase in standardized DBD score (e.g., equivalent of an increase of 0.17 points on the Conduct Problems subscale of the Strength and Difficulties Questionnaire; Mieloo et al., 2012).

The present study complements previous meta-analyses on experimental (i.e., RCTs) and “*non*-quasi-experimental” (i.e., correlational) studies (Cooke et al., 2022; Mingebach et al., 2018; Pinquart, 2017; Rothbaum & Weisz, 1994). Quasi-experimental studies can address some of the limitations of these other research designs. For example, many “*non*-quasi-experimental” studies do not adequately adjust for confounding (e.g., genetic or environmental confounders) or address reverse causality (i.e., parent- vs. child-driven effects; Jaffee et al., 2012). In comparison, quasi-experimental studies can account for shared genetics (e.g., family-based studies) and environmental confounders (e.g., baseline characteristics via fixed effects). Experimental studies often use relatively small clinical or at-risk samples, limiting generalizability. In contrast, quasi-experimental studies frequently use large samples from population-based cohort studies (Bärnighausen, Tugwell, et al., 2017). In this way, quasi-experimental studies allow us to study normative types of parenting practices and subclinical DBD symptoms, which can complement the evidence provided by experimental studies.

The present study included 45 studies, allowing us to identify potential sources of heterogeneity in the association between negative parenting practices and DBD symptoms. We observed four key findings. First, our results did not differ depending on offspring sex, offspring age, type of DBD symptom, or whether mother’s or father’s parenting was assessed. Although DBDs are more prevalent in boys than girls (Polanczyk et al., 2015), our results are consistent with previous studies in showing that this sex difference does not arise because negative parenting practices are more strongly associated with boys’ DBDs than girls’ DBDs (Lysenko et al., 2013; Pinquart, 2017). Similarly, the effect of parenting did not vary depending on the age of offspring at either exposure or outcome assessment, nor the time between exposure and outcome assessments. This is consistent with research suggesting that the effects of parenting interventions are similar across a wide range of ages (Gardner et al., 2019). The effect of paternal parenting is much less researched than maternal parenting, and therefore, our findings of no difference between maternal and paternal parenting address a key gap in the literature (Jeong et al., 2016, 2021). Finally, the effect of parenting practices did not vary according to the different outcomes (e.g., CP, ODD, externalizing symptoms), suggesting that preventative parenting interventions are likely to have a similar impact on different types of DBDs.

Second, our findings indicate that the estimated effect of negative parenting on disruptive behavior varied according to the type of quasi-experimental method used. Different quasi-experimental methods account for specific types of confounding and can help us understand more fully the association between parenting practices and DBDs. Among the four types of quasi-experimental methods included in the moderator analyses, larger effect sizes were observed for adoption and discordant sibling designs than for discordant twin and within-person fixed effects designs. The designs that reported the largest effects are those that provide the least control for confounding. For example, adoption studies do not automatically control for genetic confounding that arises from evocative gene–environment correlations or environmental confounders such as the prenatal environment (Thapar & Rice, 2021). In comparison, designs that yielded smaller pooled estimates, such as discordant twin studies, offer more control for genetic and shared environmental confounding (McAdams et al., 2021). For instance, fixed effects studies adjust for all time-invariant unmeasured confounding, both genetic and environmental, as each individual acts as their own control (Gunasekara et al., 2014). However, because only two studies in our sample used fixed effects, firm conclusions about this design cannot be drawn.

In line with prior work (Jaffee et al., 2012), our moderator analyses on the type of quasi-experimental method suggest that some of the effects of negative parenting on DBD symptoms reported in previous research are likely to reflect genetic and environmental confounding. Our findings make it clear that no single quasi-experimental method can address all confounders (Goetghebeur et al., 2020; Lawlor et al., 2016; Munafò & Davey Smith, 2018). This underscores the importance of triangulating across different methods and developing novel quasi-experimental approaches, in particular designs such as *g*-methods that have not yet been implemented.

Third, our results highlight the potential impact of shared method variance (Podsakoff et al., 2003). An equal number of studies relied on discordant versus concordant raters. The results from the moderator analyses suggested that reported effects were nearly twice as large when the informants were the same compared to when they were different. This is consistent with previous meta-analyses on other mental health measures (Francis et al., 2023; Schoeler et al., 2018). In nonquasi-experimental studies, reports of parenting practices and offspring DBDs tend to come from a single source, which likely inflates estimates of association. This may explain why previous meta-analyses of nonquasi-experimental studies report larger effect sizes compared to the pooled effect we report from quasi-experimental evidence.

Fourth, the higher the quality of the study, the smaller the reported effects. The study quality was deemed higher with better control for confounders, when different informants were included for the exposure and outcome and when the study included observational measures that are not prone to recall bias. In addition, higher quality studies were also more likely to be longitudinal and control for preexisting levels of offspring DBDs. This reduces the likelihood of reverse causation (i.e., child-driven effects) whereby children who have more DBD symptoms evoke more negative parenting behavior (as shown by bidirectional associations in family-based cross-lagged models; Zvara et al., 2018). Taken together, our findings suggest that future research on the impact of parenting on DBDs must strive to account for genetic and environmental confounding and child-driven effects by triangulating across different study designs and analyses (e.g., discordant twin design, fixed effects analysis).

Although RCTs of parenting interventions cannot be directly compared to quasi-experimental studies of parenting practices, it is important to discuss the reasons that our estimates are lower than those reported in previous meta-analyses of RCTs (Mingebach et al., 2018). First, RCTs do not only target parenting practices, but they also influence other potential risk factors for offspring DBDs, such as parental relationship quality and parental psychopathology (Jeong et al., 2021; Weber et al., 2019). The estimates included in the current meta-analysis measured the effect of positive and negative parenting practices, with many studies controlling for other variables, such as parental symptoms of depression and marital conflict.

Second, RCTs often use at-risk or clinical samples, and intervention effects tend to be stronger when offspring DBD symptoms are more severe (Menting et al., 2013). For example, in a meta-analysis of RCTs, the magnitude of effects increased as the “level of prevention” increased from universal (i.e., community samples; *r* = −0.104), selected (i.e., families with higher levels of risk factors for offspring DBDs; *r* = −0.134), indicated (i.e., families with emerging offspring DBDs; *r* = −0.265), and finally treatment prevention programs (i.e., families [self-] referred to outpatient clinics; *r* = −0.326; Leijten et al., 2019). Consequently, the smaller effects identified in the present study may reflect the characteristics of the samples included, which were predominantly community-based samples, where DBD symptoms are typically less severe than in clinical populations.

Finally, positive parenting had a small and nonsignificant effect on offspring DBD symptoms (pooled *r* = −0.06; 95% CI [−0.15, 0.03]). This result differs from RCTs, which include positive parenting practices as one of the key components (Leijten et al., 2019, 2022). Although our analyses for positive parenting were less powered than for negative parenting (positive parenting standard error [*SE*] = 0.0427, negative parenting *SE* = 0.0180), the pooled estimate was derived from 18 studies including 21,100 individuals. Furthermore, although nonsignificant, the results were in the expected direction, with higher levels of positive parenting practices related to fewer DBD symptoms. Emphasizing positive parenting practices may be particularly important in targeted prevention programs for families with emerging or current DBD symptoms, whereas universal prevention programs may benefit from reducing negative parenting practices. It is also likely that the therapeutic parenting support offered by parenting programs is not directly comparable to parenting practices as they naturally occur in the community. Interventions represent a case of “what can be,” rather than “what is.” They may enable parents to be substantially more consistent and targeted in their behavior than is typically observed in the community. This could, in part, contribute to larger parenting effects in RCTs compared with quasi-experimental studies.

### Implications for Prevention and Intervention Strategies for DBDs

Although the present study did not directly examine parenting interventions, we believe that the findings may provide insight for current interventions, especially universal prevention efforts. Fathers are underrepresented in parenting interventions; our results suggest that changing fathers’ parenting should have as much of a beneficial effect on preventing or reducing offspring DBD symptoms as changing mothers’ parenting (Lundahl et al., 2008; Panter-Brick et al., 2014). Our findings also suggest that in nonclinical samples, interventions that focus solely on promoting positive parenting practices may be less effective than those that also focus on reducing negative parenting practices.

Although the present study provides evidence consistent with a causal effect of negative parenting practices on DBDs, this effect is small in magnitude. This finding strongly suggests that there are many other causal influences for the development of DBDs. Candidate risk factors include peer deviance, parental psychopathology, and social disadvantage (Jaffee et al., 2012). Future research should quantitatively synthesize the quasi-experimental evidence for these factors to better understand how multiple causal influences come together to influence both risk and resilience.

Finally, even a small causal effect can have a substantial public health impact (Carey et al., 2023). We estimate that a 0.33 *SD* reduction of negative parenting practices, an effect size commonly observed in universal parenting interventions (Jeong et al., 2021), could result in approximately 4.5 million school-aged children worldwide no longer meeting clinical thresholds for DBD symptoms. Given the long-term adverse consequences of DBDs (Fairchild et al., 2019), preventing even a small fraction of the population from developing these symptoms is expected to have large downstream benefits for individuals and society (Burt et al., 2018; Funder & Ozer, 2019). In addition, the benefits of effective prevention programs could be exponential across generations, as more children are exposed to models of parenting that do not rely on negative practices.

### Limitations

While this quantitative review of quasi-experimental evidence of the association between parenting practices and offspring DBDs is novel, it is characterized by several limitations. First, we included a wide range of quasi-experimental methods, which may have introduced additional between-study heterogeneity due to variation in target populations and methodologies. To address this, we conducted sensitivity checks, including leave-one-out and moderator analyses, to identify potential sources of heterogeneity and adjust our analyses accordingly. Second, both parenting practices and DBD symptoms were primarily assessed via questionnaires, which can imprecisely capture the intended constructs and are vulnerable to recall bias. To mitigate this imprecision, we only included studies that used well-validated measures of exposure and outcome. In addition, the estimates from questionnaire-based measures were similar to those from observational measures and semistructured interviews, such as the Five-Minute Speech Sample (Gottschalk & Gleser, 1979). Third, we were unable to examine whether the findings were moderated by participant ancestry, due to limited reporting across studies. Even where data were reported, the majority of participants were of White ancestry, limiting the generalizability of the findings. Future research must find ways to improve diversity in research participation, and studies should provide information on the ancestry of their samples. This is essential for building evidence that informs equitable practice (Wellcome, 2021). Fourth, although quasi-experimental studies can more effectively control for potential confounders than observational studies, no single design rules out all unmeasured confounding. Triangulating across quasi-experimental methods with differing assumptions and sources of bias can increase confidence in findings (Goetghebeur et al., 2020; Lawlor et al., 2016; Munafò & Davey Smith, 2018). Fifth, due to limited resources, we were unable to include non-English language studies in our search, which may have led to the exclusion of relevant research conducted in other languages. This presents a potential limitation, as it could introduce cultural or regional biases. Recent advancements in translation technologies are likely to make the inclusion of non-English studies more feasible in future reviews. Finally, our reliance on published literature may increase the risk of publication bias, as studies with null or negative findings are less likely to appear in peer-reviewed journals. Future research syntheses would benefit from incorporating both non-English and unpublished studies to improve the representativeness of the evidence base.

### Conclusions

This meta-analysis of quasi-experimental evidence suggests that negative parenting has a small, harmful effect on DBDs. Interventions that target negative parenting practices could prevent approximately 4.5 million clinical cases of DBDs worldwide and substantially reduce the considerable economic, health, and social burden of DBDs. Future research using quasi-experimental designs will be valuable in identifying other modifiable causes of DBDs, which, along with reducing negative parenting practices, could be incorporated into preventative interventions for DBDs.

## Supplementary Material

10.1037/bul0000495.supp

## Figures and Tables

**Table 1 tbl1:** Selected Characteristics of Quasi-Experimental Studies of Parenting Practices and Offspring Disruptive Behavior Disorder Symptoms

Reference	Cohort	Country	Ind	Obs	ES	Method	Sex	Design	Bias	Positive	Negative
Anthony et al. (2019)	Wales Adoption Cohort Study	U.K.	62	62	1	Adopt	Mixed	Long	7.0	High parental warmth	
Asbury et al. (2003)	Twins Early Development Study	U.K.	4,268	2,134	2	Twin	Mixed	Cross	5.0		Harsh discipline; negative parental feeling
Asbury et al. (2006)	Twins Early Development Study	U.K.	4,090	2,045	3	Twin	Mixed (estimates available for males and females separately)	Long	7.0	Effective parent–child communication	Negative parental feeling; harsh discipline
Barnett and Scaramella (2013)	Fragile Families and Child Wellbeing Study	USA	274	137	2	Sib	Mixed (estimates available for males and females separately)	Long	6.0	High parental warmth	Coercive parenting
Besemer et al. (2016)	Pittsburgh Youth Study	USA	499	499	3	FE	Males	Long	8.5		Harsh discipline; low parental involvement; poor parent–child communication
Boisvert and Wright (2008)	Panel Study of Income Dynamics—Child Development Supplement Study	USA	578	289	2	Sib	Mixed (estimates available for males and females separately)	Long	7.0	High parental warmth; high parental monitoring	
Boyle et al. (2004)	Ontario Child Health Study and National Longitudinal Survey of Children and Youth and National Longitudinal Survey of Youth	Canada	7,392	3,696	4	Sib	Mixed	Cross	6.0	High parental involvement; high parental warmth	Harsh discipline; parental hostility
Burt et al. (2006)	Minnesota Twin Family Study	USA	901	901	1	Twin	Mixed (estimates available for males and females separately)	Long	6.5		High parent–child conflict
Burt et al. (2021)	Twin Study of Behavioral and Emotional Development in Children and Twin Study of Behavioral and Emotional Development in Adolescents	USA	480	240	1	Twin	Mixed	Cross	4.5		High parent–child conflict
Caspi et al. (2004)	Environmental Risk Longitudinal Twin Study	U.K.	1,212	606	3	Twin	Mixed	Long	9.5	Positive parental feeling; high parental warmth	Parental criticism
Cecil et al. (2012)	Twins Early Development Study	U.K.	5,184	2,592	2	Twin	Mixed	Long	7.0		Harsh discipline; negative parental feeling
Cree et al. (2021)	Early Growth and Development Study	USA	337	337	1	Adopt	Mixed	Long	8.0		Overreactive parenting
Deater-Deckard and Petrill (2004)	Northeast–Northwest Collaborative Adoption Projects Study	USA	224	224	1	Adopt	Mixed	Long	5.5	Positive parent–child relationship	
Ganiban et al. (2021)	Early Growth and Development Study	USA	361	361	2	Adopt	Mixed	Long	8.0		Low parental involvement; overreactive parenting
Glover et al. (2010)	Northeast–Northwest Collaborative Adoption Projects Study	USA	85	85	2	Adopt	Mixed	Cross	5.0	Positive parental feeling	Negative parental feeling
Harold et al. (2012)	Cardiff in-vitro fertilization (CardiffIVF) study	U.K./USA	207	207	1	IVF	Mixed	Cross	5.0		Parental hostility
Harold et al. (2013)	Northeast–Northwest Collaborative Adoption Projects Study	USA	218	218	1	Adopt	Mixed	Cross	7.5		Parental hostility
Hou et al. (2013)	Beijing Twin Study	China	690	345	2	Twin	Mixed	Long	9.5	High parental warmth	Parental hostility
Klahr, McGue, et al. (2011)	Sibling Interaction and Behavior Study	USA	672	405	1	Adopt	Mixed	Long	9.5		High parent–child conflict
Klahr, Rueter, et al. (2011)	Sibling Interaction and Behavior Study	USA	396	396	2	Adopt	Mixed	Cross	6.5		High parent–child conflict; coercive parenting
Kullberg et al. (2024)	Twins Early Development Study	U.K.	5,698	2,849	1	Twin	Mixed	Long	7.5		Harsh discipline
Latham et al. (2017)	The Twins, Family and Behavior Study	U.K.	212	212	1	Sib	Mixed	Long	7.5		Coercive parenting
Lipscomb et al. (2014)	Early Growth and Development Study	USA	233	233	1	Adopt	Mixed	Long	7.0		Overreactive parenting
Long et al. (2015)	Virginia Adult Twin Study of Psychiatric and Substance Use Disorders	USA	2,606	1,303	4	Twin	Mixed	Cross	5.5		Low parental warmth; overreactive parenting; overprotective parenting; harsh discipline
Lysenko et al. (2013)	Twins Early Development Study	U.K.	9,096	4,548	1	Sib	Separate male and female samples	Long	6.5		Harsh discipline
Marceau et al. (2013)	Early Growth and Development Study	USA	561	561	1	Adopt	Mixed	Long	7.0		Overreactive parenting
Mark and Pike (2017)	Sisters and Brothers Study	U.K.	156	78	2	Sib	Mixed	Long	5.5	Positive parent–child relationship	High parent–child conflict
Meunier et al. (2012)	Healthy Babies Healthy Children Study	Canada	809	599	1	Sib	Mixed	Cross	6.0	Positive parent–child relationship	
Morcillo et al. (2011)	Boricua Youth Study	USA/Puerto Rico	653	653	1	PSM	Separate male and female samples	Long	9.0	High family bonding	
Narusyte et al. (2011)	Twin and Offspring Study in Sweden and Twin Study of Child and Adolescent Development	Sweden	3,540	3,540	1	CoT	Mixed	Long	7.0		Parental criticism
Oliver (2015)	Twins Early Development Study	U.K.	6,308	3,154	1	Twin	Mixed	Long	7.0		Negative parental feeling
Paine et al. (2021)	Wales Adoption Cohort Study	U.K.	96	96	1	Adopt	Mixed	Long	8.5	High parental warmth	
Pike et al. (1996)	Nonshared Environment and Adolescent Development Study	USA	186	93	1	Twin	Mixed	Cross	5.0		High parent–child conflict
Reuben et al. (2016)	Early Growth and Development Study	USA	225	225	2	Adopt	Mixed	Long	7.5	High parental warmth	Overreactive parenting
Richmond and Stocker (2006)	Not reported	Not reported	186	93	1	Sib	Mixed	Long	4.5		High parent–child conflict
Richmond and Stocker (2009)	Not reported	Not reported	228	114	1	Sib	Mixed	Long	4.5		High parent–child conflict
Riggins-Caspers et al. (2003)	Not reported	Not reported	150	150	1	Adopt	Mixed	Long	5.0		Harsh discipline
Rolon-Arroyo et al. (2018)	Not reported	USA	162	162	1	FE	Mixed	Long	7.0		Overreactive parenting
Roos et al. (2016)	Early Growth and Development Study	USA	293	293	1	Adopt	Mixed	Long	8.5		Low parental involvement
Samek et al. (2014)	Sibling Interaction and Behavior Study	USA	533	533	3	Adopt	Mixed	Long	7.0		Low parental involvement; high parent–child conflict; negative parent–child relationship
Shelton et al. (2008)	Cardiff Study of All Wales and North West of United Kingdom Twins	U.K.	462	217	2	Twin	Mixed	Long	7.0		High parental hostility; low parental warmth
Shewark et al. (2021)	Early Growth and Development Study	USA	561	561	2	Adopt	Mixed	Long	7.0	High parental warmth	Parental hostility
Viding et al. (2009)	Twins Early Development Study	U.K.	4,056	2,028	1	Twin	Mixed	Cross	8.5		Harsh discipline
Vrolijk et al. (2020)	Research on Adolescent Development And Relationships Study	The Netherlands	497	497	1	CL	Mixed	Long	9.0	High autonomy support	
Waller et al. (2018)	Michigan State University Twin Registry Study	USA	374	187	2	Twin	Mixed	Cross	5.5	High parental warmth	Harsh discipline
*Note*. Ind = number of individuals; Obs = number of observations; ES = number of effect sizes; Methods: Adopt = adoption study; CL = cross-lagged panel model; CoT = children of twins study; Sib = discordant sibling study; Twin = discordant twin study; IVF = in-vitro fertilization study; PSM = propensity score matching; FE = within-individual fixed effects; Design: Long = longitudinal; Cross = cross-sectional.											

**Table 2 tbl2:** Descriptive Summary of the Positive and Negative Parenting Practices Measured in the Included Studies

Measure	*k* (%)	ES (%)
Positive parenting measures		
High versus low parental warmth	10 (50%)	15 (43%)
Positive versus negative parent–child relationship	3 (15%)	4 (11%)
Positive versus negative parental feeling	2 (10%)	3 (9%)
High versus low autonomy support	1 (5%)	4 (11%)
High versus low family bonding	1 (5%)	4 (11%)
Effective versus poor parent–child communication	1 (5%)	2 (6%)
High versus low parental involvement	1 (5%)	2 (6%)
High versus low parental monitoring	1 (5%)	1 (3%)
Total	17	35
Negative parenting measures		
High versus low harsh discipline	10 (19%)	23 (19%)
High versus low parent–child conflict	9 (17%)	41 (34%)
High versus low parental hostility	7 (14%)	15 (13%)
Overreactive versus calm parenting	7 (14%)	10 (8%)
Negative versus positive parental feeling	5 (10%)	6 (5%)
Low versus high parental involvement	4 (8%)	4 (3%)
High versus low coercive parenting	3 (6%)	5 (4%)
High versus low parental criticism	2 (4%)	6 (5%)
Low versus high parental warmth	2 (4%)	6 (5%)
High versus low overprotective parenting	1 (2%)	2 (2%)
Negative versus positive parent–child relationship	1 (2%)	1 (1%)
Poor versus effective parent–child relationship	1 (2%)	1 (1%)
Total	38	120
*Note*. Some studies reported estimates for both positive and negative parenting practices. *k* = number of studies; ES = number of effect sizes.		

**Table 3 tbl3:** Descriptive Summary of the Participant Characteristics and Study Features of the Included Studies

Characteristic	*k*	%
Mean percentage female		48.1
Mean percentage of mothers		79.3
Mean percentage of fathers		19.9
Majority ancestry^a^		
White	22	48.9
Asian	4	8.9
Hispanic	1	2.2
African American	1	2.2
Not reported	17	37.8
Year of publication		
1995–1999	1	2.2
2000–2004	5	11.1
2005–2009	7	15.6
2010–2014	15	33.3
2015–2019	10	22.2
2020–2024	7	15.6
Geographical region^b^		
USA	24	51.1
U.K.	14	29.8
Canada	2	4.3
China	1	2.1
Sweden	1	2.1
Puerto Rico	1	2.1
The Netherlands	1	2.1
Not reported	3	6.4
Cohort^b^		
Early Growth and Development Study	8	16.7
Twins Early Development Study	7	14.6
Sibling Interaction and Behavior Study	3	6.2
Twin Study of Behavioral and Emotional Development in Children	3	6.2
Northeast–Northwest Collaborative Adoption Projects	2	4.2
Wales Adoption Cohort Study	2	4.2
Beijing Twin Study	1	2.1
Boricua Youth Study	1	2.1
Cardiff in-vitro fertilization study	1	2.1
Cardiff Study of All Wales and North West of England Twins	1	2.1
Environmental Risk Longitudinal Twin Study	1	2.1
Fragile Families and Child Wellbeing Study	1	2.1
Healthy Babies Healthy Children	1	2.1
Minnesota Twin Family Study	1	2.1
National Longitudinal Study of Youth	1	2.1
National Longitudinal Survey of Children and Youth	1	2.1
Nonshared Environment and Adolescent Development project	1	2.1
Ontario Child Health Study	1	2.1
Panel Study of Income Dynamics—Child Development Supplement study	1	2.1
Pittsburgh Youth Study	1	2.1
Research on Adolescent Development And Relationships	1	2.1
Sisters and Brothers Study	1	2.1
The Twins, Family and Behavior	1	2.1
Twin Study of Behavioral and Emotional Development in Adolescents	1	2.1
Twin and Offspring Study in Sweden and from the Twin Study of Child and Adolescent Development both from the Swedish Twin Registry	1	2.1
Virginia Adult Twin Study of Psychiatric and Substance Use Disorders	1	2.1
Not reported	4	8.3
Quasi-experimental method^b^		
Adoption study	16	35.6
Discordant twin study	15	33.3
Discordant sibling study	9	20
Within-person fixed effects	2	4.4
Extended children of twins study	1	2.2
In-vitro fertilization study	1	2.2
Propensity score matching	1	2.2
Study design^b^		
Longitudinal	34	73.9
Cross-sectional	12	26.1
Informants for the exposure and outcome^b^		
Concordant	26	50.0
Discordant	26	50.0
Number of covariates in analyses		
0	9	20.0
1	6	13.3
2	14	31.1
3	5	11.1
4	4	8.9
5	3	6.7
6	3	6.7
7	1	2.2
Type of covariates^b^		
Child sex	21	20.0
Child age	18	17.1
Prior disruptive behavior disorder	8	7.6
Adoption factors	8	7.6
Marital status/quality	7	6.7
Other factors	7	6.7
Socioeconomic factors	6	5.7
Prior parenting	6	5.7
Obstetric complications	6	5.7
Ethnicity	4	3.8
Other parenting factors	3	2.9
Parental psychopathology	3	2.9
In utero exposure to toxins	3	2.9
Home environment	2	1.9
Interactions between variables	1	1.0
Birth order	1	1.0
Parent age	1	1.0
*Note*. *k* = number of studies; % = percentage.		
^a^ Calculated from the total number of cohorts. ^b^ Calculated from the total number of effect sizes.		

**Table 4 tbl4:** Meta-Analytic Associations Between Negative Parenting Practices and Offspring Disruptive Behavior Disorder Symptoms for the Variables Included in the Moderator Analyses

Term	*k*	ES	Ind	*r*	95% CI	
					*LL*	*UL*
Offspring sex						
Intercept	33	91	34,641	0.132	0.077	0.187
Increasing % female	33	91	34,641	0.000	−0.001	0.001
Age at outcome assessment						
Intercept	37	118	35,201	0.198	0.131	0.264
Increasing age	37	118	35,201	−0.004	−0.009	0.000
Time between assessments	37	118	35,201	−0.010	−0.024	0.005
Age at exposure assessment						
Intercept	37	118	35,201	0.198	0.131	0.264
Increasing age	37	118	35,201	−0.004	−0.009	0.000
Time between assessments	37	118	35,201	−0.014	−0.029	0.001
Time between assessments						
Intercept	37	118	35,201	0.152	0.106	0.197
Increasing time	37	118	35,201	−0.012	−0.027	0.003
Type of DBD outcome						
Conduct problems	14	34	11,642	0.146	0.086	0.205
Antisocial personality disorder	2	9	3,139	0.050	−0.075	0.174
Conduct disorder	3	8	2,918	0.084	−0.034	0.202
Externalizing behavior	16	35	20,174	0.135	0.078	0.193
Oppositional defiant disorder	3	5	811	0.131	−0.002	0.264
Other DBD	3	29	956	0.133	−0.002	0.268
Type of quasi-experimental method						
Discordant twin study	14	70	13,271	0.082	0.03	0.135
Adoption study	13	26	1,468	0.185	0.121	0.25
Discordant sibling study	7	15	22,362	0.174	0.091	0.257
Within-person fixed effects	2	5	661	0.068	−0.063	0.199
Informant for exposure and outcome						
Concordant	23	66	30,280	0.173	0.131	0.215
Discordant	21	54	14,303	0.087	0.043	0.131
Data quality						
High quality	22	50	14,318	0.105	0.056	0.153
High risk	5	18	23,063	0.192	0.099	0.286
Very high risk	11	52	8,792	0.154	0.085	0.223
Maternal versus paternal parenting						
Combined	14	25	8,117	0.113	0.054	0.172
Maternal	25	67	33,953	0.140	0.096	0.184
Paternal	12	28	8,278	0.151	0.094	0.208
*Note*. *k* = number of studies; ES = number of effect sizes; Ind = number of individuals; *r* = Pearson’s *r* correlation; CI = confidence interval; *LL* = lower limit; *UL* = upper limit; DBD = disruptive behavior disorder.						

## References

[ref1] *Anthony R. E., Paine A. L., & Shelton K. H. (2019). Adverse childhood experiences of children adopted from care: The importance of adoptive parental warmth for future child adjustment. International Journal of Environmental Research and Public Health, 16(12), Article 2212. 10.3390/ijerph16122212 31234480 PMC6617038

[ref2] *Asbury K., Dunn J. F., Pike A., & Plomin R. (2003). Nonshared environmental influences on individual differences in early behavioral development: A monozygotic twin differences study. Child Development, 74(3), 933–943. 10.1111/1467-8624.00577 12795399

[ref3] *Asbury K., Dunn J. F., & Plomin R. (2006). Birthweight-discordance and differences in early parenting relate to monozygotic twin differences in behaviour problems and academic achievement at age 7. Developmental Science, 9(2), F22–F31. 10.1111/j.1467-7687.2006.00469.x 16472310

[ref4] Assink M., & Wibbelink C. J. M. (2016). Fitting three-level meta-analytic models in R: A step-by-step tutorial. The Quantitative Methods for Psychology, 12(3), 154–174. 10.20982/tqmp.12.3.p154

[ref5] Bandura A., & Walters R. H. (1963). Social learning and personality development. Holt, Rinehart & Winston.

[ref6] *Barnett M. A., & Scaramella L. V. (2013). Mothers’ parenting and child sex differences in behavior problems among African American preschoolers. Journal of Family Psychology, 27(5), 773–783. 10.1037/a0033792 23937420 PMC3981992

[ref7] Bärnighausen T., Røttingen J.-A., Rockers P., Shemilt I., & Tugwell P. (2017). Quasi-experimental study designs series-paper 1: Introduction: Two historical lineages. Journal of Clinical Epidemiology, 89, 4–11. 10.1016/j.jclinepi.2017.02.020 28694121

[ref8] Bärnighausen T., Tugwell P., Røttingen J.-A., Shemilt I., Rockers P., Geldsetzer P., Lavis J., Grimshaw J., Daniels K., Brown A., Bor J., Tanner J., Rashidian A., Barreto M., Vollmer S., & Atun R. (2017). Quasi-experimental study designs series-paper 4: Uses and value. Journal of Clinical Epidemiology, 89, 21–29. 10.1016/j.jclinepi.2017.03.012 28365303

[ref9] *Besemer S., Loeber R., Hinshaw S. P., & Pardini D. A. (2016). Bidirectional associations between externalizing behavior problems and maladaptive parenting within parent-son dyads across childhood. Journal of Abnormal Child Psychology, 44(7), 1387–1398. 10.1007/s10802-015-0124-6 26780209 PMC5790196

[ref10] *Boisvert D., & Wright J. P. (2008). Nonshared environmental influences on sibling differences in externalizing problem behavior. Criminal Justice and Behavior, 35(7), 863–878. 10.1177/0093854808318495

[ref11] *Boyle M. H., Jenkins J. M., Georgiades K., Cairney J., Duku E., & Racine Y. (2004). Differential-maternal parenting behavior: Estimating within- and between-family effects on children. Child Development, 75(5), 1457–1476. 10.1111/j.1467-8624.2004.00751.x 15369525

[ref12] *Burt S. A., Clark D. A., Gershoff E. T., Klump K. L., & Hyde L. W. (2021). Twin differences in harsh parenting predict youth’s antisocial behavior. Psychological Science, 32(3), 395–409. 10.1177/0956797620968532 33577745 PMC8726584

[ref13] Burt S. A., Hyde L. W., Frick P. J., Jaffee S. R., Shaw D. S., & Tremblay R. (2018). Commentary: Childhood conduct problems are a public health crisis and require resources: A commentary on Rivenbark et al. Journal of Child Psychology and Psychiatry, 59(6), 711–713. 10.1111/jcpp.12930 29808490

[ref14] *Burt S. A., McGue M., Iacono W. G., & Krueger R. F. (2006). Differential parent–child relationships and adolescent externalizing symptoms: Cross-lagged analyses within a monozygotic twin differences design. Developmental Psychology, 42(6), 1289–1298. 10.1037/0012-1649.42.6.1289 17087561 PMC2365490

[ref15] Carey E. G., Ridler I., Ford T. J., & Stringaris A. (2023). Editorial perspective: When is a ‘small effect’ actually large and impactful? Journal of Child Psychology and Psychiatry, 64(11), 1643–1647. 10.1111/jcpp.13817 37226639

[ref16] *Caspi A., Moffitt T. E., Morgan J., Rutter M., Taylor A., Arseneault L., Tully L., Jacobs C., Kim-Cohen J., & Polo-Tomas M. (2004). Maternal expressed emotion predicts children’s antisocial behavior problems: Using monozygotic-twin differences to identify environmental effects on behavioral development. Developmental Psychology, 40(2), 149–161. 10.1037/0012-1649.40.2.149 14979757

[ref17] *Cecil C. A. M., Barker E. D., Jaffee S. R., & Viding E. (2012). Association between maladaptive parenting and child self-control over time: Cross-lagged study using a monozygotic twin difference design. The British Journal of Psychiatry, 201(4), 291–297. 10.1192/bjp.bp.111.107581 22918964

[ref18] Cooke J. E., Deneault A. A., Devereux C., Eirich R., Fearon R. M. P., & Madigan S. (2022). Parental sensitivity and child behavioral problems: A meta-analytic review. Child Development, 93(5), 1231–1248. 10.1111/cdev.13764 35357693

[ref19] *Cree R. A., Liu C., Gueorguieva R., Neiderhiser J. M., Leve L. D., Connell C. M., Shaw D. S., Natsuaki M. N., Ganiban J. M., Beekman C., Smith M. V., & Reiss D. (2021). Using an adoption design to test genetically based differences in risk for child behavior problems in response to home environmental influences. Development and Psychopathology, 33(4), 1229–1247. 10.1017/S0954579420000450 32654671 PMC8983395

[ref20] *Deater-Deckard K., & Petrill S. A. (2004). Parent–child dyadic mutuality and child behavior problems: An investigation of gene–environment processes. Journal of Child Psychology and Psychiatry, 45(6), 1171–1179. 10.1111/j.1469-7610.2004.00309.x 15257673

[ref21] De Los Reyes A., Henry D. B., Tolan P. H., & Wakschlag L. S. (2009). Linking informant discrepancies to observed variations in young children’s disruptive behavior. Journal of Abnormal Child Psychology, 37(5), 637–652. 10.1007/s10802-009-9307-3 19247829 PMC3734944

[ref22] Eyberg S. M., Nelson M. M., & Boggs S. R. (2008). Evidence-based psychosocial treatments for children and adolescents with disruptive behavior. Journal of Clinical Child and Adolescent Psychology, 37(1), 215–237. 10.1080/15374410701820117 18444059

[ref23] Fairchild G., Hawes D. J., Frick P. J., Copeland W. E., Odgers C. L., Franke B., Freitag C. M., & De Brito S. A. (2019). Conduct disorder. Nature Reviews Disease Primers, 5(1), Article 43. 10.1038/s41572-019-0095-y 31249310

[ref24] Francis E. R., Tsaligopoulou A., Stock S. E., Pingault J., & Baldwin J. R. (2023). Subjective and objective experiences of childhood adversity: A meta-analysis of their agreement and relationships with psychopathology. Journal of Child Psychology and Psychiatry, 64(8), 1185–1199. 10.1111/jcpp.13803 37186463 PMC10617978

[ref25] Funder D. C., & Ozer D. J. (2019). Evaluating effect size in psychological research: Sense and nonsense. Advances in Methods and Practices in Psychological Science, 2(2), 156–168. 10.1177/2515245919847202

[ref26] *Ganiban J. M., Liu C., Zappaterra L., An S., Natsuaki M. N., Neiderhiser J. M., Reiss D., Shaw D. S., & Leve L. D. (2021). Gene × Environment interactions in the development of preschool effortful control, and its implications for childhood externalizing behavior. Behavior Genetics, 51(5), 448–462. 10.1007/s10519-021-10073-9 34160711 PMC8915202

[ref27] Gardner F., Leijten P., Melendez-Torres G. J., Landau S., Harris V., Mann J., Beecham J., Hutchings J., & Scott S. (2019). The earlier the better? Individual participant data and traditional meta-analysis of age effects of parenting interventions. Child Development, 90(1), 7–19. 10.1111/cdev.13138 30216433

[ref28] *Glover M. B., Mullineaux P. Y., Deater-Deckard K., & Petrill S. A. (2010). Parents’ feelings towards their adoptive and non-adoptive children. Infant and Child Development, 19(3), 238–251. 10.1002/icd.664 21088705 PMC2982262

[ref29] Goetghebeur E., le Cessie S., De Stavola B., Moodie E. E., Waernbaum I., & the “on behalf of” the Topic Group Causal Inference (TG7) of the STRATOS Initiative. (2020). Formulating causal questions and principled statistical answers. Statistics in Medicine, 39(30), 4922–4948. 10.1002/sim.8741 32964526 PMC7756489

[ref30] Gottschalk L. A., & Gleser G. C. (1979). The measurement of psychological states through the content analysis of verbal behavior. University of California Press.

[ref31] Gunasekara F. I., Richardson K., Carter K., & Blakely T. (2014). Fixed effects analysis of repeated measures data. International Journal of Epidemiology, 43(1), 264–269. 10.1093/ije/dyt221 24366487

[ref32] *Harold G. T., Elam K. K., Lewis G., Rice F., & Thapar A. (2012). Interparental conflict, parent psychopathology, hostile parenting, and child antisocial behavior: Examining the role of maternal versus paternal influences using a novel genetically sensitive research design. Development and Psychopathology, 24(4), 1283–1295. 10.1017/S0954579412000703 23062297

[ref33] *Harold G. T., Leve L. D., Elam K. K., Thapar A., Neiderhiser J. M., Natsuaki M. N., Shaw D. S., & Reiss D. (2013). The nature of nurture: Disentangling passive genotype-environment correlation from family relationship influences on children’s externalizing problems. Journal of Family Psychology, 27(1), 12–21. 10.1037/a0031190 23421830 PMC3576129

[ref34] Hernán M. A., & Robins J. M. (2016). Using big data to emulate a target trial when a randomized trial is not available. American Journal of Epidemiology, 183(8), 758–764. 10.1093/aje/kwv254 26994063 PMC4832051

[ref35] Higgins J. P., Thomas J., Chandler J., Cumpston M., Li T., Page M. J., & Welch V. A. (2019). Cochrane handbook for systematic reviews of interventions. Wiley. 10.1002/9781119536604 PMC10284251 31643080

[ref36] Hipwell A., Keenan K., Kasza K., Loeber R., Stouthamer-Loeber M., & Bean T. (2008). Reciprocal influences between girls’ conduct problems and depression, and parental punishment and warmth: A six year prospective analysis. Journal of Abnormal Child Psychology, 36(5), 663–677. 10.1007/s10802-007-9206-4 18172753 PMC2572202

[ref37] *Hou J., Chen Z., Natsuaki M. N., Li X., Yang X., Zhang J., & Zhang J. (2013). A longitudinal investigation of the associations among parenting, deviant peer affiliation, and externalizing behaviors: A monozygotic twin differences design. Twin Research and Human Genetics, 16(3), 698–706. 10.1017/thg.2013.24 23659853

[ref38] Jaffee S. R., Strait L. B., & Odgers C. L. (2012). From correlates to causes: Can quasi-experimental studies and statistical innovations bring us closer to identifying the causes of antisocial behavior? Psychological Bulletin, 138(2), 272–295. 10.1037/a0026020 22023141 PMC3268012

[ref39] Jeong J., Franchett E. E., Ramos de Oliveira C. V., Rehmani K., & Yousafzai A. K. (2021). Parenting interventions to promote early child development in the first three years of life: A global systematic review and meta-analysis. PLOS Medicine, 18(5), Article e1003602. 10.1371/journal.pmed.1003602 33970913 PMC8109838

[ref40] Jeong J., McCoy D. C., Yousafzai A. K., Salhi C., & Fink G. (2016). Paternal stimulation and early child development in low- and middle-income countries. Pediatrics, 138(4), Article e20161357. 10.1542/peds.2016-1357 27600319

[ref41] Karwatowska L. (2025). Positive and negative parenting practices and offspring disruptive behavior: A meta-analytic review of quasi-experimental evidence [Data set]. GitHub. https://github.com/lk1373190/parenting_dbd_qe_meta.git 10.1037/bul0000495 PMC12720486 41428512

[ref42] Karwatowska L., Russell S., Solmi F., De Stavola B. L., Jaffee S., Pingault J.-B., & Viding E. (2020). Risk factors for disruptive behaviours: Protocol for a systematic review and meta-analysis of quasi-experimental evidence. BMJ Open, 10(9), Article e038258. 10.1136/bmjopen-2020-038258 PMC7482491 32907905

[ref43] Karwatowska L., Russell S., Stavola B. D., Jaffee S., Pingault J.-B., & Viding E. (2024). Risk factors for disruptive behaviours: A systematic review and meta-analysis of quasi-experimental evidence. PROSPERO. https://www.crd.york.ac.uk/PROSPERO/view/CRD42020169313 10.1136/bmjopen-2020-038258 PMC7482491 32907905

[ref44] *Klahr A. M., McGue M., Iacono W. G., & Burt S. A. (2011). The association between parent–child conflict and adolescent conduct problems over time: Results from a longitudinal adoption study. Journal of Abnormal Psychology, 120(1), 46–56. 10.1037/a0021350 21038930 PMC3035729

[ref45] *Klahr A. M., Rueter M. A., McGue M., Iacono W. G., & Alexandra Burt S. (2011). The relationship between parent–child conflict and adolescent antisocial behavior: Confirming shared environmental mediation. Journal of Abnormal Child Psychology, 39(5), 683–694. 10.1007/s10802-011-9505-7 21484334 PMC3102125

[ref46] *Kullberg M. J., Van Schie C. C., Allegrini A. G., Ahmadzadeh Y., Wechsler D. L., Elzinga B. M., & McAdams T. A. (2024). Comparing findings from the random-intercept cross-lagged panel model and the monozygotic twin difference cross-lagged panel model: Maladaptive parenting and offspring emotional and behavioural problems. JCPP Advances, 4(1), Article e12203. 10.1002/jcv2.12203 38486957 PMC10933702

[ref47] *Latham R. M., Mark K. M., & Oliver B. R. (2017). A harsh parenting team? Maternal reports of coparenting and coercive parenting interact in association with children’s disruptive behaviour. Journal of Child Psychology and Psychiatry, 58(5), 603–611. 10.1111/jcpp.12665 27917470

[ref48] Lawlor D. A., Tilling K., & Davey Smith G. (2016). Triangulation in aetiological epidemiology. International Journal of Epidemiology, 45(6), 1866–1886. 10.1093/ije/dyw314 28108528 PMC5841843

[ref49] Leijten P., Gardner F., Melendez-Torres G. J., van Aar J., Hutchings J., Schulz S., Knerr W., & Overbeek G. (2019). Meta-analyses: Key parenting program components for disruptive child behavior. Journal of the American Academy of Child & Adolescent Psychiatry, 58(2), 180–190. 10.1016/j.jaac.2018.07.900 30738545

[ref50] Leijten P., Melendez-Torres G. J., & Gardner F. (2022). Research review: The most effective parenting program content for disruptive child behavior—A network meta-analysis. Journal of Child Psychology and Psychiatry, 63(2), 132–142. 10.1111/jcpp.13483 34240409

[ref51] *Lipscomb S. T., Laurent H., Neiderhiser J. M., Shaw D. S., Natsuaki M. N., Reiss D., & Leve L. D. (2014). Genetic vulnerability interacts with parenting and early care education to predict increasing externalizing behavior. International Journal of Behavioral Development, 38(1), 70–80. 10.1177/0165025413508708 25067867 PMC4109820

[ref52] Lipsey M. W., & Wilson D. B. (2001). Practical meta-analysis. Sage Publications.

[ref53] *Long E. C., Aggen S. H., Gardner C., & Kendler K. S. (2015). Differential parenting and risk for psychopathology: A monozygotic twin difference approach. Social Psychiatry and Psychiatric Epidemiology, 50(10), 1569–1576. 10.1007/s00127-015-1065-7 25940788 PMC4591114

[ref54] Lundahl B. W., Tollefson D., Risser H., & Lovejoy M. C. (2008). A meta-analysis of father involvement in parent training. Research on Social Work Practice, 18(2), 97–106. 10.1177/1049731507309828

[ref55] *Lysenko L. J., Barker E. D., & Jaffee S. R. (2013). Sex differences in the relationship between harsh discipline and conduct problems. Social Development, 22(1), 197–214. 10.1111/sode.12002

[ref56] *Marceau K., Hajal N., Leve L. D., Reiss D., Shaw D. S., Ganiban J. M., Mayes L. C., & Neiderhiser J. M. (2013). Measurement and associations of pregnancy risk factors with genetic influences, postnatal environmental influences, and toddler behavior. International Journal of Behavioral Development, 37(4), 366–375. 10.1177/0165025413489378 24839336 PMC4018759

[ref57] *Mark K. M., & Pike A. (2017). Links between marital quality, the mother–child relationship and child behavior. International Journal of Behavioral Development, 41(2), 285–294. 10.1177/0165025416635281

[ref58] McAdams T. A., Rijsdijk F. V., Zavos H. M. S., & Pingault J.-B. (2021). Twins and causal inference: Leveraging nature’s experiment. Cold Spring Harbor Perspectives in Medicine, 11(6), Article a039552. 10.1101/cshperspect.a039552 32900702 PMC8168524

[ref59] Menting A. T. A., Orobio de Castro B., & Matthys W. (2013). Effectiveness of the incredible years parent training to modify disruptive and prosocial child behavior: A meta-analytic review. Clinical Psychology Review, 33(8), 901–913. 10.1016/j.cpr.2013.07.006 23994367

[ref60] *Meunier J. C., Bisceglia R., & Jenkins J. M. (2012). Differential parenting and children’s behavioral problems: Curvilinear associations and mother–father combined effects. Developmental Psychology, 48(4), 987–1002. 10.1037/a0026321 22122474

[ref61] Mieloo C., Raat H., van Oort F., Bevaart F., Vogel I., Donker M., & Jansen W. (2012). Validity and reliability of the strengths and difficulties questionnaire in 5–6 year olds: Differences by gender or by parental education? PLOS ONE, 7(5), Article e36805. 10.1371/journal.pone.0036805 22629332 PMC3356337

[ref62] Mingebach T., Kamp-Becker I., Christiansen H., & Weber L. (2018). Meta-meta-analysis on the effectiveness of parent-based interventions for the treatment of child externalizing behavior problems. PLOS ONE, 13(9), Article e0202855. 10.1371/journal.pone.0202855 30256794 PMC6157840

[ref63] Moffitt T. E., Caspi A., Rutter M., & Silva P. A. (2001). Sex differences in antisocial behaviour: Conduct disorder, delinquency, and violence in the Dunedin longitudinal study. Cambridge University Press. 10.1017/CBO9780511490057

[ref64] *Morcillo C., Duarte C. S., Shen S., Blanco C., Canino G., & Bird H. R. (2011). Parental familism and antisocial behaviors: Development, gender, and potential mechanisms. Journal of the American Academy of Child & Adolescent Psychiatry, 50(5), 471–479. 10.1016/j.jaac.2011.01.014 21515196 PMC4391499

[ref65] Munafò M. R., & Davey Smith G. (2018). Robust research needs many lines of evidence. Nature, 553(7689), 399–401. 10.1038/d41586-018-01023-3 32094809

[ref66] *Narusyte J., Neiderhiser J. M., Andershed A.-K., D’Onofrio B. M., Reiss D., Spotts E., Ganiban J., & Lichtenstein P. (2011). Parental criticism and externalizing behavior problems in adolescents: The role of environment and genotype-environment correlation. Journal of Abnormal Psychology, 120(2), 365–376. 10.1037/a0021815 21280930 PMC3093313

[ref67] *Oliver B. R. (2015). Unpacking externalising problems: Negative parenting associations for conduct problems and irritability. BJPsych Open, 1(1), 42–47. 10.1192/bjpo.bp.115.000125 26435845 PMC4589139

[ref68] Oliver B. R., Trzaskowski M., & Plomin R. (2014). Genetics of parenting: The power of the dark side. Developmental Psychology, 50(4), 1233–1240. 10.1037/a0035388 24364831 PMC3977675

[ref69] *Paine A. L., Perra O., Anthony R., & Shelton K. H. (2021). Charting the trajectories of adopted children’s emotional and behavioral problems: The impact of early adversity and postadoptive parental warmth. Development and Psychopathology, 33(3), 922–936. 10.1017/S0954579420000231 32366341 PMC8374623

[ref70] Panter-Brick C., Burgess A., Eggerman M., McAllister F., Pruett K., & Leckman J. F. (2014). Practitioner review: Engaging fathers—Recommendations for a game change in parenting interventions based on a systematic review of the global evidence. Journal of Child Psychology and Psychiatry, 55(11), 1187–1212. 10.1111/jcpp.12280 24980187 PMC4277854

[ref71] Patterson G. R. (1982). Coercive family process. Castalia Publishing.

[ref72] Pettit G. S., Bates J. E., & Dodge K. A. (1997). Supportive parenting, ecological context, and children’s adjustment: A seven-year longitudinal study. Child Development, 68(5), 908–923. 10.2307/1132041 29106716

[ref73] *Pike A., Reiss D., Hetherington E. M., & Plomin R. (1996). Using MZ differences in the search for nonshared environmental effects. Journal of Child Psychology and Psychiatry, 37(6), 695–704. 10.1111/j.1469-7610.1996.tb01461.x 8894950

[ref74] Pilling S., Gould N., Whittington C., Taylor C., Scott S., & the Guideline Development Group. (2013). Recognition, intervention, and management of antisocial behaviour and conduct disorders in children and young people: Summary of NICE-SCIE guidance. British Medical Journal, 346, Article f1298. 10.1136/bmj.f1298 23535256

[ref75] Pingault J.-B., Richmond R., & Davey Smith G. (2022). Causal inference with genetic data: Past, present, and future. Cold Spring Harbor Perspectives in Medicine, 12(3), Article a041271. 10.1101/cshperspect.a041271 34580080 PMC8886738

[ref76] Pinquart M. (2017). Associations of parenting dimensions and styles with externalizing problems of children and adolescents: An updated meta-analysis. Developmental Psychology, 53(5), 873–932. 10.1037/dev0000295 28459276

[ref77] Podsakoff P. M., MacKenzie S. B., Lee J.-Y., & Podsakoff N. P. (2003). Common method biases in behavioral research: A critical review of the literature and recommended remedies. Journal of Applied Psychology, 88(5), 879–903. 10.1037/0021-9010.88.5.879 14516251

[ref78] Polanczyk G. V., Salum G. A., Sugaya L. S., Caye A., & Rohde L. A. (2015). Annual research review: A meta-analysis of the worldwide prevalence of mental disorders in children and adolescents. Journal of Child Psychology and Psychiatry, 56(3), 345–365. 10.1111/jcpp.12381 25649325

[ref79] *Reuben J. D., Shaw D. S., Neiderhiser J. M., Natsuaki M. N., Reiss D., & Leve L. D. (2016). Warm parenting and effortful control in toddlerhood: Independent and interactive predictors of school-age externalizing behavior. Journal of Abnormal Child Psychology, 44(6), 1083–1096. 10.1007/s10802-015-0096-6 26496906 PMC5097859

[ref80] *Richmond M. K., & Stocker C. M. (2006). Associations between family cohesion and adolescent siblings’ externalizing behavior. Journal of Family Psychology, 20(4), 663–669. 10.1037/0893-3200.20.4.663 17176202

[ref81] *Richmond M. K., & Stocker C. M. (2009). Associations between siblings’ differential family experiences and differences in psychological adjustment. International Journal of Developmental Science, 3(2), 98–114. 10.3233/DEV-2009-3202

[ref82] *Riggins-Caspers K. M., Cadoret R. J., Knutson J. F., & Langbehn D. (2003). Biology-environment interaction and evocative biology-environment correlation: Contributions of harsh discipline and parental psychopathology to problem adolescent behaviors. Behavior Genetics, 33(3), 205–220. 10.1023/A:1023434206261 12837013

[ref83] Rissanen E., Kuvaja-Köllner V., Elonheimo H., Sillanmäki L., Sourander A., & Kankaanpää E. (2022). The long-term cost of childhood conduct problems: Finnish nationwide 1981 birth cohort study. Journal of Child Psychology and Psychiatry, 63(6), 683–692. 10.1111/jcpp.13506 34402045

[ref84] Rivenbark J. G., Odgers C. L., Caspi A., Harrington H., Hogan S., Houts R. M., Poulton R., & Moffitt T. E. (2018). The high societal costs of childhood conduct problems: Evidence from administrative records up to age 38 in a longitudinal birth cohort. Journal of Child Psychology and Psychiatry, 59(6), 703–710. 10.1111/jcpp.12850 29197100 PMC5975095

[ref85] Rodgers M. A., & Pustejovsky J. E. (2021). Evaluating meta-analytic methods to detect selective reporting in the presence of dependent effect sizes. Psychological Methods, 26(2), 141–160. 10.1037/met0000300 32673040

[ref86] Rodrigues M., Sokolovic N., Madigan S., Luo Y., Silva V., Misra S., & Jenkins J. (2021). Paternal sensitivity and children’s cognitive and socioemotional outcomes: A meta-analytic review. Child Development, 92(2), 554–577. 10.1111/cdev.13545 33511634

[ref87] *Rolon-Arroyo B., Arnold D. H., Breaux R. P., & Harvey E. A. (2018). Reciprocal relations between parenting behaviors and conduct disorder symptoms in preschool children. Child Psychiatry and Human Development, 49(5), 786–799. 10.1007/s10578-018-0794-8 29468356 PMC6497406

[ref88] *Roos L. E., Fisher P. A., Shaw D. S., Kim H. K., Neiderhiser J. M., Reiss D., Natsuaki M. N., & Leve L. D. (2016). Inherited and environmental influences on a childhood co-occurring symptom phenotype: Evidence from an adoption study. Development and Psychopathology, 28(1), 111–125. 10.1017/S0954579415000322 25851306 PMC4598247

[ref89] Rothbaum F., & Weisz J. R. (1994). Parental caregiving and child externalizing behavior in nonclinical samples: A meta-analysis. Psychological Bulletin, 116(1), 55–74. 10.1037/0033-2909.116.1.55 8078975

[ref90] *Samek D. R., Keyes M. A., Hicks B. M., Bailey J., McGue M., & Iacono W. G. (2014). General and specific predictors of nicotine and alcohol dependence in early adulthood: Genetic and environmental influences. Journal of Studies on Alcohol and Drugs, 75(4), 623–634. 10.15288/jsad.2014.75.623 24988261 PMC4108603

[ref91] Schoeler T., Duncan L., Cecil C. M., Ploubidis G. B., & Pingault J.-B. (2018). Quasi-experimental evidence on short- and long-term consequences of bullying victimization: A meta-analysis. Psychological Bulletin, 144(12), 1229–1246. 10.1037/bul0000171 30475016

[ref92] Scott J. K., Nelson J. A., & Dix T. (2018). Interdependence among mothers, fathers, and children from early to middle childhood: Parents’ sensitivity and children’s externalizing behavior. Developmental Psychology, 54(8), 1528–1541. 10.1037/dev0000525 29927264

[ref93] Shamseer L., Moher D., Clarke M., Ghersi D., Liberati A., Petticrew M., Shekelle P., Stewart L. A., & the PRISMA-P Group. (2015). Preferred reporting items for systematic review and meta-analysis protocols (PRISMA-P) 2015: Elaboration and explanation. British Medical Journal, 349, Article g7647. 10.1136/bmj.g7647 25555855

[ref94] *Shelton K. H., Harold G. T., Fowler T. A., Rice F. J., Neale M. C., Thapar A., & van den Bree M. B. M. (2008). Parent–child relations, conduct problems and cigarette use in adolescence: Examining the role of genetic and environmental factors on patterns of behavior. Journal of Youth and Adolescence, 37(10), 1216–1228. 10.1007/s10964-007-9254-7

[ref95] *Shewark E. A., Ramos A. M., Liu C., Ganiban J. M., Fosco G., Shaw D. S., Reiss D., Natsuaki M. N., Leve L. D., & Neiderhiser J. M. (2021). The role of child negative emotionality in parenting and child adjustment: Gene–environment interplay. Journal of Child Psychology and Psychiatry, 62(12), 1453–1461. 10.1111/jcpp.13420 33821495 PMC8492791

[ref96] Stroup D. F., Berlin J. A., Morton S. C., Olkin I., Williamson G. D., Rennie D., Moher D., Becker B. J., Sipe T. A., & Thacker S. B. (2000). Meta-analysis of observational studies in epidemiology: A proposal for reporting. meta-analysis of observational studies in epidemiology (MOOSE) group. Journal of the American Medical Association, 283(15), 2008–2012. 10.1001/jama.283.15.2008 10789670

[ref97] Thapar A., & Rice F. (2021). Family-based designs that disentangle inherited factors from pre- and postnatal environmental exposures: In vitro fertilization, discordant sibling pairs, maternal versus paternal comparisons, and adoption designs. Cold Spring Harbor Perspectives in Medicine, 11(3), Article a038877. 10.1101/cshperspect.a038877 32152247 PMC7919395

[ref98] *Viding E., Fontaine N. M. G., Oliver B. R., & Plomin R. (2009). Negative parental discipline, conduct problems and callous-unemotional traits: Monozygotic twin differences study. The British Journal of Psychiatry, 195(5), 414–419. 10.1192/bjp.bp.108.061192 19880931

[ref99] Viechtbauer W. (2010). Conducting meta-analyses in *R* with the metafor package. Journal of Statistical Software, 36(3), 1–48. 10.18637/jss.v036.i03

[ref100] Viechtbauer W., & Cheung M. W.-L. (2010). Outlier and influence diagnostics for meta-analysis. Research Synthesis Methods, 1(2), 112–125. 10.1002/jrsm.11 26061377

[ref101] *Vrolijk P., Van Lissa C. J., Branje S. J. T., Meeus W. H. J., & Keizer R. (2020). Longitudinal linkages between father and mother autonomy support and adolescent problem behaviors: Between-family differences and within-family effects. Journal of Youth and Adolescence, 49(11), 2372–2387. 10.1007/s10964-020-01309-8 32876868 PMC7538400

[ref102] Wachs T. D., Georgieff M., Cusick S., & McEwen B. S. (2014). Issues in the timing of integrated early interventions: Contributions from nutrition, neuroscience, and psychological research. Annals of the New York Academy of Sciences, 1308(1), 89–106. 10.1111/nyas.12314 24354763 PMC4075015

[ref103] *Waller R., Hyde L. W., Klump K. L., & Burt S. A. (2018). Parenting is an environmental predictor of callous-unemotional traits and aggression: A monozygotic twin differences study. Journal of the American Academy of Child & Adolescent Psychiatry, 57(12), 955–963. 10.1016/j.jaac.2018.07.882 30522741 PMC6296820

[ref104] Weber L., Kamp-Becker I., Christiansen H., & Mingebach T. (2019). Treatment of child externalizing behavior problems: A comprehensive review and meta-meta-analysis on effects of parent-based interventions on parental characteristics. European Child & Adolescent Psychiatry, 28(8), 1025–1036. 10.1007/s00787-018-1175-3 29948228

[ref105] Wellcome. (2021). Clinical trials policy—Grant funding. https://wellcome.org/grant-funding/guidance/clinical-trials-policy

[ref106] Wells G. A., Shea B., O’Connell D., Peterson J., Welch V., Losos M., & Tugwell P. (2000). The Newcastle-Ottawa Scale (NOS) for assessing the quality of nonrandomised studies in meta-analyses. Oxford University Press.

[ref107] Williamson H. C., Bradbury T. N., Trail T. E., & Karney B. R. (2011). Factor analysis of the Iowa Family Interaction Rating Scales. Journal of Family Psychology, 25(6), 993–999. 10.1037/a0025903 21988081 PMC7673103

[ref108] Zvara B. J., Sheppard K. W., & Cox M. (2018). Bidirectional effects between parenting sensitivity and child behavior: A cross-lagged analysis across middle childhood and adolescence. Journal of Family Psychology, 32(4), 484–495. 10.1037/fam0000372 29697996 PMC7466908

