# Supplementary material for: Positive and Negative Parenting Practices and Offspring Disruptive Behavior: A Meta-Analytic Review of Quasi-Experimental Evidence
Source: Psychol Bull. 2025 Nov;151(11):1363–81. doi: 10.1037/bul0000495 (PMC12720486; doi:10.1037/bul0000495)
Supplement: Supplementary file 1 [file bul0000495_Supplemental_Materials.docx]

**Supplementary Material**

**Positive and negative parenting and offspring disruptive behaviour: A meta-analytic review of quasi-experimental evidence.**

Table of Contents

[Tables 2](#_Toc204957400)

[Reporting Guidelines 2](#_Toc204957401)

[**Table S1.** Preferred Reporting Items for Systematic reviews and Meta-Analyses (PRISMA) reporting checklist. 2](#_Toc204957402)

[**Table S2.** Meta-Analyses of Observational Studies in Epidemiology (MOOSE) reporting checklist. 5](#_Toc204957403)

[Definitions of key terms 7](#_Toc204957404)

[**Table S3.** A summary of the key quasi-experimental study types used in the meta-analyses. 7](#_Toc204957405)

[**Table S4.** A summary of the key features commonly associated with quasi-experimental studies. 11](#_Toc204957406)

[**Table S5.** A summary of the disruptive behaviour disorder symptoms considered in the review. 12](#_Toc204957407)

[Search strategy 13](#_Toc204957408)

[**Table S6.** Strategy used in the database searches. 13](#_Toc204957409)

[Risk of Bias Assessment 15](#_Toc204957410)

[**Table S7.** The original Newcastle-Ottowa Scale for Cohort Studies (right) and the adapted version (left) that we used in the current study. Changes are shown in blue. 15](#_Toc204957411)

[Effect size conversion 17](#_Toc204957412)

[**Table S8.** Formulae used to convert original study data into Pearson’s r and SE. 17](#_Toc204957413)

[Results 18](#_Toc204957414)

[**Table S9.** Studies Excluded After Full-Text Screening and Reasons for Exclusion 18](#_Toc204957415)

[**Table S10.** Summary of all the effect sizes included in the meta-analyses of positive parenting practices. 49](#_Toc204957416)

[**Table S11.** Summary of all the effect sizes included in the meta-analyses of negative parenting practices. 52](#_Toc204957417)

[**Table S12.** Descriptive summary of the participant characteristics and study features of the studies included in the meta-analysis by risk of bias category. 61](#_Toc204957418)

[Figures 63](#_Toc204957419)

[**Figure S1.** A simplified representation of the data structure for the meta-analysis 63](#_Toc204957420)

[**Figure S2.** Population attributable impact of negative parenting. 64](#_Toc204957421)

[**Figure S3.** PRISMA flow diagram of search results 65](#_Toc204957422)

[**Figure S4.** Funnel plots for studies reporting effect estimates for positive parenting (2A; k [number of studies]= 17; ES [number of effect sizes] = 35) and those reporting effect estimates for negative parenting (2B; k = 38; ES = 122). The different colours represent different cohorts. 66](#_Toc204957423)

[**Figure S5.** Leave-one-out sensitivity analyses assessing whether any effect size had undue influence on the meta-analytic results for positive parenting (left) or negative parenting (right) 67](#_Toc204957424)

[**Figure S6.** Leave-one-out sensitivity analyses assessing whether any study had undue influence on the meta-analytic results for positive parenting (left) or negative parenting (right) 68](#_Toc204957425)

[**Figure S7.** Leave-one-out sensitivity analyses assessing whether any cohort had undue influence on the meta-analytic results for positive parenting (left) or negative parenting (right). 69](#_Toc204957426)

[**Figure S8.** Funnel plots for studies reporting effect estimates for negative parenting practices in studies categorised as very high-risk (S4A; k [number of studies] =11 ; ES [number of effect sizes] = 52), high-risk (S4B; k = 5 ; = 18) and high-quality (S4C, k = 22; ES =50 ). The different colours represent different cohorts. 70](#_Toc204957427)

[**Figure S9.** Forest plot of the disruptive behaviour disorder outcome subgroup analyses. 71](#_Toc204957428)

[**Figure S10.** Forest plot of the quasi-experimental study type subgroup analyses. 72](#_Toc204957429)

[**Figure S11.** Forest plot of the informant type subgroup analyses. 73](#_Toc204957430)

[**Figure S12.** Forest plot of the study quality subgroup analyses. 74](#_Toc204957431)

[**Figure S13.** Forest plot of the maternal versus paternal parenting practices subgroup analyses. 75](#_Toc204957432)

# Tables

## Reporting Guidelines

### **Table S1.** Preferred Reporting Items for Systematic reviews and Meta-Analyses (PRISMA) reporting checklist.

| **Section and Topic** | **Item #** | **Checklist item** | **Location where item is reported** |
| --- | --- | --- | --- |
| **TITLE** | | |  |
| Title | 1 | Identify the report as a systematic review. | 1 |
| **ABSTRACT** | | |  |
| Abstract | 2 | See the PRISMA 2020 for Abstracts checklist. | 2 |
| **INTRODUCTION** | | |  |
| Rationale | 3 | Describe the rationale for the review in the context of existing knowledge. | 4 |
| Objectives | 4 | Provide an explicit statement of the objective(s) or question(s) the review addresses. | 6 |
| **METHODS** | | |  |
| Eligibility criteria | 5 | Specify the inclusion and exclusion criteria for the review and how studies were grouped for the syntheses. | 9 |
| Information sources | 6 | Specify all databases, registers, websites, organisations, reference lists and other sources searched or consulted to identify studies. Specify the date when each source was last searched or consulted. | 11 |
| Search strategy | 7 | Present the full search strategies for all databases, registers and websites, including any filters and limits used. | Table S4 |
| Selection process | 8 | Specify the methods used to decide whether a study met the inclusion criteria of the review, including how many reviewers screened each record and each report retrieved, whether they worked independently, and if applicable, details of automation tools used in the process. | 11 |
| Data collection process | 9 | Specify the methods used to collect data from reports, including how many reviewers collected data from each report, whether they worked independently, any processes for obtaining or confirming data from study investigators, and if applicable, details of automation tools used in the process. | 11 |
| Data items | 10a | List and define all outcomes for which data were sought. Specify whether all results that were compatible with each outcome domain in each study were sought (e.g. for all measures, time points, analyses), and if not, the methods used to decide which results to collect. | 11 |
|  | 10b | List and define all other variables for which data were sought (e.g. participant and intervention characteristics, funding sources). Describe any assumptions made about any missing or unclear information. | 10 |
| Study risk of bias assessment | 11 | Specify the methods used to assess risk of bias in the included studies, including details of the tool(s) used, how many reviewers assessed each study and whether they worked independently, and if applicable, details of automation tools used in the process. | 12 |
| Effect measures | 12 | Specify for each outcome the effect measure(s) (e.g. risk ratio, mean difference) used in the synthesis or presentation of results. | 12 |
| Synthesis methods | 13a | Describe the processes used to decide which studies were eligible for each synthesis (e.g. tabulating the study intervention characteristics and comparing against the planned groups for each synthesis (item #5)). | NA |
|  | 13b | Describe any methods required to prepare the data for presentation or synthesis, such as handling of missing summary statistics, or data conversions. | Table S6 |
|  | 13c | Describe any methods used to tabulate or visually display results of individual studies and syntheses. | NA |
|  | 13d | Describe any methods used to synthesize results and provide a rationale for the choice(s). If meta-analysis was performed, describe the model(s), method(s) to identify the presence and extent of statistical heterogeneity, and software package(s) used. | 12 |
|  | 13e | Describe any methods used to explore possible causes of heterogeneity among study results (e.g. subgroup analysis, meta-regression). | 14 |
|  | 13f | Describe any sensitivity analyses conducted to assess robustness of the synthesized results. | 13 |
| Reporting bias assessment | 14 | Describe any methods used to assess risk of bias due to missing results in a synthesis (arising from reporting biases). | 12 |
| Certainty assessment | 15 | Describe any methods used to assess certainty (or confidence) in the body of evidence for an outcome. | NA |
| **RESULTS** | | |  |
| Study selection | 16a | Describe the results of the search and selection process, from the number of records identified in the search to the number of studies included in the review, ideally using a flow diagram. | Figure S1 |
|  | 16b | Cite studies that might appear to meet the inclusion criteria, but which were excluded, and explain why they were excluded. | NA |
| Study characteristics | 17 | Cite each included study and present its characteristics. | Table 1 |
| Risk of bias in studies | 18 | Present assessments of risk of bias for each included study. | Table 1 |
| Results of individual studies | 19 | For all outcomes, present, for each study: (a) summary statistics for each group (where appropriate) and (b) an effect estimate and its precision (e.g. confidence/credible interval), ideally using structured tables or plots. | Figures 1 and 2 |
| Results of syntheses | 20a | For each synthesis, briefly summarise the characteristics and risk of bias among contributing studies. | Table 3 |
|  | 20b | Present results of all statistical syntheses conducted. If meta-analysis was done, present for each the summary estimate and its precision (e.g. confidence/credible interval) and measures of statistical heterogeneity. If comparing groups, describe the direction of the effect. | 24 -31 |
|  | 20c | Present results of all investigations of possible causes of heterogeneity among study results. | 26 - 31 |
|  | 20d | Present results of all sensitivity analyses conducted to assess the robustness of the synthesized results. | 26 - 31 |
| Reporting biases | 21 | Present assessments of risk of bias due to missing results (arising from reporting biases) for each synthesis assessed. | Figures S3 and S4 |
| Certainty of evidence | 22 | Present assessments of certainty (or confidence) in the body of evidence for each outcome assessed. | NA |
| **DISCUSSION** | | |  |
| Discussion | 23a | Provide a general interpretation of the results in the context of other evidence. | 33 - 41 |
|  | 23b | Discuss any limitations of the evidence included in the review. | 40 - 41 |
|  | 23c | Discuss any limitations of the review processes used. | 40 - 41 |
|  | 23d | Discuss implications of the results for practice, policy, and future research. | 39 |
| **OTHER INFORMATION** | | |  |
| Registration and protocol | 24a | Provide registration information for the review, including register name and registration number, or state that the review was not registered. | 9 |
|  | 24b | Indicate where the review protocol can be accessed, or state that a protocol was not prepared. | 9 |
|  | 24c | Describe and explain any amendments to information provided at registration or in the protocol. | 9 |
| Support | 25 | Describe sources of financial or non-financial support for the review, and the role of the funders or sponsors in the review. | 42 |
| Competing interests | 26 | Declare any competing interests of review authors. | 42 |
| Availability of data, code and other materials | 27 | Report which of the following are publicly available and where they can be found: template data collection forms; data extracted from included studies; data used for all analyses; analytic code; any other materials used in the review. | 9 |

### **Table S2.** Meta-Analyses of Observational Studies in Epidemiology (MOOSE) reporting checklist.

| **Recommendation** | **Pg. no.** |
| --- | --- |
| **Reporting background should include** | |
| Problem definition | 6 |
| Hypothesis statement | 6 |
| Description of study outcome(s) | 11 |
| Type of exposure or intervention used | 10 |
| Type of study designs used | 10 |
| Study population | 9-10 |
| **Reporting of search strategy should include** | |
| Qualifications of searchers (e.g. librarians and investigators) | 11 |
| Search strategy, including time period included in the synthesis and keywords | 11 |
| Effort to include all available studies, including contact with authors | 11 |
| Databases and registries searched | 11 |
| Search software used, name and version, including special features | 11 |
| Use of hand searching (e.g. reference lists of obtained articles) | NA |
| List of citations located and those excluded including justification | NA |
| Method of addressing articles published in languages other than English | NA (only English language included) |
| Method of handling abstracts and unpublished studies | NA (only published studies included) |
| Description of any contact with authors | 43 |
| **Reporting methods should include** | |
| Description of relevance or appropriateness of studies assembled for assessing the hypothesis to be tested | 10; Table S3 |
| Rationale for the selection and coding of data | 9 – 10 |
| Documentation of how data were classified and coded (eg, multiple raters, blinding, and interrater reliability) | 11 |
| Assessment of confounding | 13 |
| Assessment of study quality, including blinding of quality assessors; stratification or regression on possible predictors of study results | 12 |
| Assessment of heterogeneity | 13-14 |
| Description of statistical methods (eg, complete description of fixed or random effects models, justification of whether the chosen models account for predictors of study results, dose-response models, or cumulative meta-analysis) in sufficient detail to be replicated | 11 |
| Provision of appropriate tables and graphics | Figures 1-3 |
| **Reporting of results should include** | |
| Graphic summarizing individual study estimates and overall estimate | Figures 2 and 3 |
| Table giving descriptive information for each study included | Table 1 |
| Results of sensitivity testing (eg, subgroup analysis) | Table 4 |
| Indication of statistical uncertainty of findings | 25 – 32 |
| **Reporting of discussion should include** | |
| Quantitative assessment of bias (eg, publication bias) | 27 - 29 |
| Justification for exclusion (eg, exclusion of non–English-language citations) | NA |
| Assessment of quality of included studies | Figures 2 and 3 |
| **Reporting of conclusions should include** | |
| Consideration of alternative explanations for observed results | 38 - 39 |
| Generalization of the conclusions (ie, appropriate for the data presented and within the domain of the literature review) | 42 |
| Guidelines for future research | 40 |
| Disclosure of funding source | 42 |

## Definitions of key terms

### **Table S3.** A summary of the key quasi-experimental study types used in the meta-analyses.

| **Term** | **Definition** | **Potential limitations** |
| --- | --- | --- |
| Adoption study | Adoption separates genetically related parents and children and places children in a different rearing environment. Adoption studies compare associations between exposures and outcomes in parents and their children that are genetically related (not adopted) and genetically unrelated (adopted) to them. [1] | - Does not control for environmental confounding, unless specified in the analyses. - Does not control for genetic confounding arising from evocative gene-environment correlations. - Assumes birth and adoptive parents have not been matched for characteristics that may influence child outcomes (e.g. SES). - Limited generalisability of findings. |
| Difference in difference study / controlled before and after study | Difference-in-differences designs, also known as controlled before and after studies, are a type of fixed effect study. The difference before and after the exposure in the exposed group is compared to the same period of time in the non-exposed group. The exposure effect is the difference between these differences. If an exposure has a harmful effect, the outcome will occur more rapidly in individuals that receive the exposure than in individuals that do not receive the exposure. [7] | - Does not control for time-variant confounders. - Does not rule out reverse causation (i.e. that offspring disruptive behaviour influences parenting practices) - Requires individual variation in the exposure over time. |
| Fixed effects | Applied to longitudinal data with repeated measures, fixed effects methods model within-individual changes over time (i.e. variation in an individual’s exposures and outcomes), as opposed to between-individual changes (i.e. variation across individuals), to remove time-invariant confounding, with each individual acting as their own control. Difference-in-difference, controlled before-and-after, experience sample and ecological momentary assessment are all examples of fixed effect analyses [3]. | - Does not control for time-variant confounders. - Does not rule out reverse causation (i.e. that offspring disruptive behaviour influences parenting practices) - Requires individual variation in the exposure over time. |
| Instrumental variable analysis | Analyses that use variables that are associated with an exposure of interest (assumption 1), do not share any common causes with the outcome (assumption 2) and affect the outcome only through the exposure (assumption 3), also known as instrumental variables. These variables can be any traits that meet the three instrumental variable assumptions, for example genetic variants (e.g. Mendelian randomisation). [5] | - Instrumental variable estimation with small sample sizes is imprecise. - Bias is introduced in large sample sizes when assumptions are slightly violated. - Less useful for cases of strong confounding. - Adequate instruments may be hard to find for most exposures. |
| Interrupted time series analysis | Interrupted time series methods use observational data collected over equally spaced intervals before and after an intervention, that is exogenous to the time series, e.g. a “natural experiment” in the real word setting. The effect of the intervention is evaluated by examining whether the data pattern (e.g. the level and slope) observed post-intervention is different to that observed pre-intervention. [6] | - Limited generalisability of findings. - Requires data that is measured at equally spaced intervals over time and at time points before and after the implementation of an intervention.   It is assumed that the outcome of interest would remain unchanged in the absence of the intervention. Therefore, any changes that could have affected the outcome in parallel (i.e. time trends) can lead to inaccurate results. |
| In vitro fertilization study | In vitro fertilization can either use either parental gametes (genetically related) or donor gametes (genetically unrelated) for fertilization. Similar to adoption studies, in vitro fertilisation studies compare associations between exposures and outcomes in parents and their children that are genetically related and genetically unrelated. [1] | - Limited generalisability of findings. - Small sample sizes. |
| Natural experiment | Natural experiments use natural randomly occurring circumstances (e.g. lottery win, policy or law change) as an exposure, which is assigned “as random”. [2] True natural experiments are very unusual therefore they are usually described as “quasi natural experiments” or observational studies with exogenous exposures. | - May be difficult to disentangle the effects of the exposure from other co-occurring risk factors, leading to a possibility of the risk factor being misidentified. - The exposure may differ to more typical experiences. - Limited generalisability of findings. - No/limited available data for many exposures and outcomes. - Choice of adequate control group(s) can be challenging. |
| Regression discontinuity analysis | If treatment allocation is based on whether a patient scores below or above a predetermined cut-off value, as opposed to randomisation, then the intervention will be randomly assigned for patients close to the threshold. [4] See also “Sharp/fuzzy design” below. | - Sensitive to misspecification. - Choice of bandwidth around the cut-off is challenging. - Treatment assignment based on more than one indicator can be problematic. |
| Discordant sibling study | On average, siblings share 50% of their segregated genetic material. Similar to twin studies, some sibling studies compare outcomes in exposed versus non-exposed siblings. [1] | - As siblings share 50% of their segregated genetic material sibling comparison designs do not fully account for genetic confounding. - Does not control for individual-level (or non-shared) confounding, unless specified in the analyses. - Parental exposures that do not vary between siblings cannot be studied. - Carryover effects (e.g. birth order) may be present. |
| Discordant twin study | On average, dizygotic twin pairs share 50% of their segregated genes compared to monozygotic twin pairs which share 100% of their genetic material. A twin that is non-exposed to a risk factor represents a natural match to their exposed co-twin. Therefore, some twin studies compare outcomes in exposed versus non-exposed pair members. [1] | - Does not control for individual-level (or non-shared) confounding, unless specified in te analyses. - Parental exposures that do not vary between siblings cannot be studied. |
| Children of twins design | The children of twins design builds upon the discordant twin study by examining whether the association between the exposure and outcome is stronger among MZ twin parents and their children or parent-child or aunt/uncle-child pairs. This design capitalises on the fact that the child is as genetically related to their parent’s twin as they are to their own parent, but they do not share the same rearing environment. | - Does not control for confounding from the nuclear family environment unless multiple children-of-twins are included. - Does not control for individual-level confounding by design. - Dyadic parental effects (e.g. divorce) cannot be studied. |
| Propensity score | A propensity score is the probability of being exposed conditional on the confounders. It has the advantage of reducing, a potentially large, number of confounders into a scalar that contains all information that is relevant for the exposure assignment in relation to the outcome. The procedure to create a propensity score includes (1) estimating a propensity score reflecting the risk of the exposure based on measured background characteristics (i.e., confounders), (2) matching exposed to non-exposed individuals with a similar propensity score in order to reach an acceptable balance of confounders across groups, before (3) estimating the association between the exposure and the outcome within matched pairs. The propensity score is used as an additional covariate in outcome regression, or as a stratifying or matching variable. Any association between the exposure and outcome should be independent of measured confounding factors included in the propensity score. Also see “Inverse probability weighting” and “Matching study”. | - Only controls for unmeasured confounders to the extent they are associated with measured confounder. - Measurement error in the confounder results in imperfect adjustment. - Can be sensitive to the matching approach implemented, with a trade-off between inexact matching (which can lead to residual confounding) and incomplete matching (which can limit generalisability and statistical power). |
| Inverse probability weighting | Inverse probability weights derived from propensity scores also remove confounding by recreating a pseudo-sample where there is no confounding. Similar to propensity score matching, inverse probability weighting involves (1) estimating a propensity score reflecting the risk of exposure to maltreatment based on measured background characteristic, and (2) estimating the association between maltreatment and mental health, after weighting for the inverse of the propensity score. Any association between maltreatment and mental health should be independent of measured confounding factors included in the propensity score.  Also see “Propensity score” and “Matching study”. | - Only controls for unmeasured confounders to the extent they are associated with measured confounder. - Measurement error in the confounder results in imperfect adjustment.   Can be sensitive to the matching approach implemented, with a trade-off between inexact matching (which can lead to residual confounding) and incomplete matching (which can limit generalisability and statistical power). |
| Matching study | Researchers can attempt to create a reasonable counterfactual by accounting for confounders via matching exposed and non-exposed participants on key variables. Propensity score matching approaches can be select appropriate matches (either to cases or non-cases or both) leading to different causal effects. Quasi-experimental designs are often combined with propensity score matching approaches. [2] See “Propensity score” | - Only controls for unmeasured confounders to the extent they are associated with measured confounder. - Measurement error in the confounder results in imperfect adjustment. - Can be sensitive to the matching approach implemented, with a trade-off between inexact matching (which can lead to residual confounding) and incomplete matching (which can limit generalisability and statistical power). |

### **Table S4.** A summary of the key features commonly associated with quasi-experimental studies.

| **Term** | **Definition** |
| --- | --- |
| Causal effect | An exposure has a causal effect on the outcome if the outcome differs when the exposure is present compared to when the exposure is absent, all other things being equal. [8] |
| Counterfactual framework | The comparison of hypothetical scenarios whereby the *same* individual is either exposed or unexposed to a risk factor. Also known as the potential outcomes framework. [8–10] |
| Doubly robust estimation | Doubly robust estimation combines two models: outcome regression and propensity score modelling. Individually these two methods lead to unbiased estimators of the causal effect only if the respective model is correctly specified; when they are combined, through doubly robust estimation, only one of the two models needs to be correctly specified to obtain an unbiased effect estimator. [11] |
| Heckit model/Heckman sample selection | Similar to selection/selectivity models, these are used to handle non-ignorable missing data. Heckit/Heckman selection models assume (a) a joint distribution for the missingness and outcome processes and (b) validity in the instrument. If these assumptions are met, these models can correct bias from non-randomly selected samples. |
| Potential outcome | The outcome that would occur had the exposure been set to a particular value; see also “Counterfactual framework”. |
| Selection/selectivity model | A model that deals with samples that are non-randomly selected, and therefore non-representative of the target population. For example, studies affected by non-ignorable missing data. |
| Sharp design/Fuzzy design | These are features of regression discontinuity designs.  A sharp discontinuity regression design exploits exogenous changes to the value of an exposure /intervention to estimate its causal effect on an outcome. These changes are usually triggered by overtaking a particular (sharp) threshold in a continuous endogenous variable. Since the comparison with the threshold may be affected by random error, individuals with values near the threshold can be viewed as being “as good as” randomly allocated to the exposure and analysed as if they were in an RCT.  In a fuzzy regression discontinuity design, the threshold does not need to be defined as a sharp discontinuity as long as the probability of exposure/intervention assignment differs among those near the threshold. |

### **Table S5.** A summary of the disruptive behaviour disorder symptoms considered in the review.

| **Term** | **Definition** |
| --- | --- |
| Disruptive behaviour disorders / externalising disorders | A range of repetitive and troublesome behaviours, such as lying, fighting and stealing. It can include one or more of the below behaviours and the term is sometimes used interchangeably with “externalising disorders”. |
| Antisocial personality disorder (DSM) / dissocial personality disorder (ICD) | A diagnosis which involves a life-long pattern of antisocial behaviour as well as irritability and remorselessness. By definition, a diagnosis of ASPD involves exhibiting conduct disorder in childhood. |
| Conduct disorders | A formal diagnosis whereby an individual displays repetitive and persistent patterns of antisocial, aggressive or defiant behaviour that amounts to significant and persistent violations of age-appropriate social expectations [12] and are diagnosable as defined by the DSM-5. |
| Conduct problems | An umbrella term to describe a range of repetitive and disruptive behaviours, such as lying, fighting and stealing that do not necessarily meet the threshold for diagnosis of conduct disorder. |
| Oppositional defiant disorder | A formal diagnosis whereby an individual exhibits defiant and disobedient behaviour towards others as opposed to conduct disorder, whereby behaviours violate the rights of others and/or societal expectations. |
| Psychopathy | Psychopathy is characterised by high levels of antisocial behaviour, low levels of anxiety and high levels of attention seeking. It is a specifier for antisocial personality disorder in the DSM-5. |

## Search strategy

### **Table S6.** Strategy used in the database searches.

| **SEARCH TERMS** | |
| --- | --- |
| **Database** | **MeSH Terms** |
| Ovid MEDLINE | **Quasi-experimental studies** – causality/; adoption/; child, adopted/; exp twins/; twin study/; propensity score/; siblings/; interrupted time series analysis/; mendelian randomization analysis/; ecological momentary assessment/; fertilization in vitro/; controlled before-after studies/; fuzzy logic/.  **Disruptive behaviour disorder symptoms** – conduct disorder/; "attention deficit and disruptive behavior disorders"/; antisocial personality disorder/; problem behavior/.  **Parenting practices** – None. |
| Ovid EMBASE | **Quasi-experimental studies** – causality/; causal attribution/; causal modelling/; quasi experimental study/; adopted child/; adoption/; twins/; twin study/; propensity score/; sibling/; sibling relation/; instrumental variable analysis/; time series analysis/; mendelian randomization analysis/; ecological momentary assessment/; in vitro fertilization/; exogenous variable/; fuzzy logic/; fuzzy system/; maximum likelihood method/.  **Disruptive behaviour disorder symptoms** – conduct disorder/; oppositional defiant disorder/; disruptive behavior/; antisocial personality disorder/; psychopathy/; problem behavior/.  **Parenting practices** – child parent relation/. |
| Ovid PsycINFO | **Quasi-experimental studies** – exp causality/; exp causal analysis/; exp quasi experimental methods/; adopted children/; “adoption (child)”/; adoptive parents/; twins/; exp siblings/; exp time series/; exp ecological momentary assessment/; reproductive technology/; counterfactual thinking/; fuzzy logic/; fuzzy set theory/; exp maximum likelihood/.  **Disruptive behaviour disorder symptoms** – exp conduct disorder/; exp oppositional defiant disorder/; exp disruptive behavior disorders/; antisocial personality disorder/; psychopathy/ behavior problems/; externalizing symptoms/.  Parenting practices - permissive parenting/; parenting/; childrearing practices/; parent child communication/; parent child relations/; parental involvement/; parenting skills/; parenting style/; authoritarian parenting/; authoritative parenting/. |
| Web of Science | **Quasi-experimental studies** – Not applicable.  **Disruptive behaviour disorder symptoms** – Not applicable. |
| **Free Text Search Terms** | |
| **Concept 1 -**  Quasi-experimental studies | 1. Quasi-experimental studies MeSH Terms 2. (causal*) 3. ((quasiexperiment*) or (quasi experiment*)) 4. (adopt*) 5. (fixed effect*) 6. (twin*) 7. (propensity score*) 8. (sibling*) 9. (regression discontinuity) 10. (instrumental variable*) 11. (interrupted time series) 12. (mendelian randomi?ation) 13. (matching stud*) 14. (experience sampl*) 15. (ecological momentary assessment*) 16. ((difference* in difference*) or (difference* stud*)) 17. (in vitro fertili?ation) 18. ((polygenic score*) or (polygenic risk score*)) 19. (exogenous varia*) 20. (natural experiment*) 21. (matched control*) 22. (counterfactual*) 23. (potential outcome*) 24. ((balancing adj3 covariate) or (imbalance adj3 covariate) or (balanced adj3 covariate) or (imbalanced adj3 covariate)) 25. (controlled before and after) or (controlled before after)) 26. (inverse probability weight*) 27. ((doubly robust regression*) or (doubly robust estimate*)) 28. ((selection model*) or (selectivity model*)) 29. ((heckit model*) or (heckman sample selection*)) 30. (selection correction*) 31. (two stage residual inclusion*) 32. ((sharp design*) or (fuzzy design*)) 33. (forcing variable*) 34. (full information maximum likelihood) 35. (natural control*) 36. 1 OR 2 OR 3 OR 4 OR 5 OR 6 OR 7 OR 8 OR 9 OR 10 OR 11 OR 12 OR 13 OR 14 OR 15 OR 16 OR 17 OR 18 OR 19 OR 20 OR 21 OR 22 OR 23 OR 24 OR 25 OR 26 OR 27 OR 28 OR 29 OR 30 OR 31 OR 32 OR 33 OR 34 OR 35 |
| **Concept 2 -**  Disruptive behaviour disorder symptoms | 1. Disruptive behaviour disorder MeSH Terms 2. (conduct problem*) 3. (conduct disorder*) 4. (oppositional defian*) 5. (disruptive behavior*) or (disruptive behaviour*) 6. (antisocial personalit*) or (anti social personalit*) 7. (dissocial personalit*) 8. ((psychopathic) or (psychopathy) or (psychopath) or (psychopaths)) 9. (problem behavior*) or (problem behaviour*) 10. (behavior* problem*) or (behaviour* problem*) 11. (externali?ing) 12. 37 OR 38 OR 39 OR 40 OR 41 OR 42 OR 43 OR 44 OR 45 OR 46 OR 47 |
| **Concept 3** – Parenting practices | 1. Parenting MeSH Terms 2. (parenting) 3. 49 OR 50 |
|  | 1. 36 AND 48 AND 51 2. Limit to English Language 3. Human not animal 4. Not review or meta-analysis or case-report 5. Limit to 1980 - Current |

## Risk of Bias Assessment

### **Table S7.** The original Newcastle-Ottowa Scale for Cohort Studies (right) and the adapted version (left) that we used in the current study. Changes are shown in blue.

| **Original Newcastle-Ottowa Scale** | **Adapted Newcastle-Ottowa Scale** |
| --- | --- |
| **Selection** | |
| 1) Representativeness of the exposed cohort  a) truly representative of the average _______________ (describe) in the community ¯  b) somewhat representative of the average ______________ in the community ¯  c) selected group of users eg nurses, volunteers  d) no description of the derivation of the cohort | 1) Representativeness of the exposed cohort  a) truly representative of the average cohort in the community (1)  b) somewhat representative of the average cohort in the community (0.5)  c) selected group of users eg nurses, volunteers (0)  d) no description of the derivation of the cohort (0) |
| 2) Selection of the non exposed cohort  a) drawn from the same community as the exposed cohort ¯  b) drawn from a different source  c) no description of the derivation of the non exposed cohort | 2) Selection of the non-exposed cohort  a) drawn from the same community as the exposed cohort (1)  b) drawn from a different source (0)  c) no description of the derivation of the non exposed cohort (0) |
| 3) Ascertainment of exposure  a) secure record (eg surgical records) ¯  b) structured interview ¯  c) written self report  d) no description | **3) Ascertainment of exposure**  **a) validated measure (1)**  **b) non-validated measure or no description (0)** |
| 4) Demonstration that outcome of interest was not present at start of study  a) yes ¯  b) no | 4) Demonstration that outcome of interest was not present **prior to exposure, or control for pre-existing outcome**  a) yes (1)  b) no (0) |
| **Comparability** | |
| 1) Comparability of cohorts on the basis of the design or analysis  a) study controls for _____________ (select the most important factor) ¯  b) study controls for any additional factor ¯ (This criteria could be modified to indicate specific  control for a second important factor.) | **1) Study accounts for the majority of environmental confounders, either by design or statistically accounting for wide range of measured variables**  **a) yes (e.g., QE study controlling for other factors) (1)**  **b) some but not all (e.g., QE study controlling for a few other factors) (0.5)**  **c) no (0)** |
|  | **2) Study fully accounts for genetic confounding**  **a) yes (e.g., MZ twin design) (1)**  **b) somewhat (e.g. DZ twin design, sibling design, or control for polygenic score or family history of outcome) (0.5)**  **c) no (0)** |
| **Outcome** | |
| 1) Assessment of outcome  a) independent blind assessment ¯  b) record linkage ¯  c) self report  d) no description | 1) Assessment of outcome  **a) validated measure (1)**  **b) non-validated measure or no description (0)** |
|  | **2) Exposure and outcome reported by different informants**  **a) yes (1)**  **b) no (0)** |
| 2) Was follow-up long enough for outcomes to occur  a) yes (select an adequate follow up period for outcome of interest) ¯  b) no | **9) Exposure and outcome were assessed at the same time**  **a) no (assessment was longitudinal – i.e., after exposure) (1)**  **b) yes – cross-sectional study / outcome assessed concurrently (0)** |
| 3) Adequacy of follow up of cohorts  a) complete follow up - all subjects accounted for ¯  b) subjects lost to follow up unlikely to introduce bias - small number lost - > ____ % (select an  adequate follow up, or description provided of those lost) ¯  c) follow up rate < ____% (select an adequate and no description of those lost  d) no statement | 10) Adequacy of follow up of cohorts  a) complete follow up - all subjects accounted for (1)  b) subjects lost to follow up unlikely to introduce bias - small number lost - > 70 % follow up, or method to account for attrition employed) (1)  c) follow up rate < 70% and no description of those lost  d) no statement (0) |

## Effect size conversion

### **Table S8.** Formulae used to convert original study data into Pearson’s r and SE.

| **Raw effect size type** | **Formulae** |
| --- | --- |
| 95% confidence intervals | $SE=\frac{uCI-lCI}{3.92}$ |
| Unstandardised beta | $SE=\frac{1-{beta}^{2}}{\sqrt{n-2}}$ |
| Unstandardised beta | $r=\frac{beta*{SD}^{EXP}}{{SD}^{OUT}}$ |

*Abbreviations: SE = standard error; uCI = upper 95% confidence interval; lCI = lower 95% confidence interval; SD^EXP^ = standard deviation of the exposure; SD^OUT^ = standard deviation of the outcome.*

## Results

### **Table S9.** Studies Excluded After Full-Text Screening and Reasons for Exclusion

| **Reference** | **Reason** |
| --- | --- |
| Accornero (2002) - Behavioral Outcome of Preschoolers Exposed Prenatally to Cocaine: Role of Maternal Behavioral Health | EXCLUDE on study design |
| Agrawal (2010) - The effects of maternal smoking during pregnancy on offspring outcomes | EXCLUDE on study design |
| Alati (2005) - Asthma and Internalizing Behavior Problems in Adolescence: A Longitudinal Study | EXCLUDE on study design |
| Alemany (2013) - Genetic origin of the relationship between parental negativity and behavior problems from early childhood to adolescence: A longitudinal genetically sensitive study | EXCLUDE on study design |
| Allen (2019) - Offspring Personality Mediates the Association between Maternal Depression and Childhood Psychopathology | EXCLUDE on study design |
| Alley (2013) - Parent–infant vocalisations at 12 months predict psychopathology at 7 years | EXCLUDE on study design |
| Amato (2014) - Estimating the Effects of Parental Divorce and Death With Fixed Effects Models | EXCLUDE on outcome |
| Anderson (2017) - Residential Mobility and Adolescent Achievement and Behavior: Understanding Timing and Extent of Mobility | EXCLUDE on risk factor |
| Ang (2014) - Gender as a Moderator of the Relationship Between Early Separation from Parents and Psychopathic Traits in a Sample of At-Risk Adolescents | EXCLUDE on study design |
| Arseneault (2006) - Bullying Victimization Uniquely Contributes to Adjustment Problems in Young Children: A Nationally Representative Cohort Study | EXCLUDE on study design |
| Atzaba-Poria (2004) - Do risk factors for problem behaviour act in a cumulativemanner? An examination of ethnic minority and majority children through an ecological perspective | EXCLUDE on study design |
| Averdijk (2018) - The long-term effects of out-of-home placement in late adolescence: A propensity score matching analysis among Swiss youths | EXCLUDE on risk factor |
| Bailey (2014) - General and substance-specific predictors of young adult nicotine dependence, alcohol use disorder, and problem behavior: Replication in two samples | EXCLUDE on study design |
| Balenzano (2021) - The relationship between preadoptive adversity and intercountry adoptees' adjustment: A mediating or moderating role of adoptive parenting? | EXCLUDE on outcome |
| Bao (2016) - Trajectories and the influencing factors of behavior problems in preschool children: a longitudinal study in Guangzhou, China | EXCLUDE on study design |
| Barker (2018) - Inflammation-related epigenetic risk and child and adolescent mental health: A prospective study from pregnancy to middle adolescence | EXCLUDE on study design |
| Barnes (2013) - Analyzing the origins of childhood externalizing behavioral problems | EXCLUDE on study design |
| Barnett (2015) - Child fear reactivity and sex as moderators of links between parenting and preschool behavior problems | EXCLUDE on study design |
| Basch (2019) - Adolescent-Reported Sleep/Wake Patterns in the Relationships Between Inhibitory Control and Internalizing and Externalizing Problems | EXCLUDE on study design |
| Bauer (2006) - Childhood Bullying Involvement and Exposure to Intimate Partner Violence | EXCLUDE on study design |
| Beaver (2015) - The Role of Parenting in the Prediction of Criminal Involvement: Findings From a Nationally Representative Sample of Youth and a Sample of Adopted Youth | EXCLUDE on outcome |
| Becker (2015) - Sleep problems predict comorbid externalizing behaviors and depression in young adolescents with attention‐deficit/ hyperactivity disorder | EXCLUDE on study design |
| Bekkhus (2018) - Re-examining the link between prenatal maternal anxiety and child emotional difficulties, using a sibling design | EXCLUDE on outcome |
| Benzies (2009) - Immediate and Sustained Effects of Parenting on Physical Aggression in Canadian Children Aged 6 Years and Younger | EXCLUDE on outcome |
| Berger (2005) - Maternity Leave, Early Maternal Employment and Child Health and Development in the US | EXCLUDE on risk factor |
| Bergmann (2016) - Maternal Weight Predicts Children’s Psychosocial Development via Parenting Stress and Emotional Availability | EXCLUDE on study design |
| Blazei (2008) - Father-Child Transmission of Antisocial Behavior: The Moderating Role of Father’s Presence in the Home | EXCLUDE on study design |
| Boeldt (2012) - The Association Between Positive Parenting and Externalizing Behaviour | EXCLUDE on study design |
| Bornovalova (2014) - Understanding the relative contributions of direct environmental effects and passive genotype–environment correlations in the association between familial risk factors and child disruptive behavior disorders | EXCLUDE on study design |
| Bornovalova, Blazei (2013) - Disentangling the Relative Contribution of Parental Antisociality and Family Discord to Child Disruptive Disorders | EXCLUDE on study design |
| Bornovalova, Hicks (2013) - Tests of a Direct Effect of Childhood Abuse on Adult Borderline Personality Disorder Traits: A Longitudinal Discordant Twin Design | EXCLUDE on outcome |
| Boutwell (2010) - Maternal Cigarette Smoking during Pregnancy and Offspring Externalizing Behavioral Problems: A Propensity Score Matching Analysis | EXCLUDE on risk factor |
| Boutwell (2011) - Prenatal exposure to cigarette smoke and childhood externalizing behavioral problems: a propensity score matching approach | EXCLUDE on risk factor |
| Boutwell (2012) - The developmental origins of externalizing behavioral problems: Parental disengagement and the role of gene–environment interplay | EXCLUDE on study design |
| Bradshaw (2020) - Longitudinal associations between parental incarceration and children's emotional and behavioural development: Results from a population cohort study | EXCLUDE on risk factor |
| Brammer (2018) - Parental ADHD and Depression: Time- Varying Prediction of Offspring Externalizing Psychopathology | EXCLUDE on study design |
| Brandlistuen (2013) - Prenatal paracetamol exposure and child neurodevelopment: a sibling-controlled cohort study | EXCLUDE on risk factor |
| Brandlistuen (2015) - Behavioural effects of fetal antidepressant exposure in a Norwegian cohort of discordant siblings | EXCLUDE on risk factor |
| Brandlistuen (2017) - Association of prenatal exposure to benzodiazepines and child internalizing problems: A sibling-controlled cohort study | EXCLUDE on risk factor |
| Branje (2004) - Perceived support in sibling relationships and adolescent adjustment | EXCLUDE on study design |
| Braza (2015) - Negative Maternal and Paternal Parenting Styles as Predictors of Children’s Behavioral Problems: Moderating Effects of the Child’s Sex | EXCLUDE on study design |
| Brendgen (2008) - Gene–environment interaction between peer victimization and child aggression | EXCLUDE on study design |
| Brensilver (2011) - Longitudinal Relations Between Depressive Symptoms and Externalizing Behavior in Adolescence: Moderating Effects of Maltreatment Experience and Gender | EXCLUDE on study design |
| Brion (2010) - Maternal Smoking and Child Psychological Problems: Disentangling Causal and Noncausal Effects | EXCLUDE on study design |
| Brion (2011) - Intrauterine Effects of Maternal Prepregnancy Overweight on Child Cognition and Behavior in 2 Cohorts | EXCLUDE on study design |
| Brody (2003) - Neighborhood Disadvantage Moderates Associations of Parenting and Older Sibling Problem Attitudes and Behavior With Conduct Disorders in African American Children | EXCLUDE on study design |
| Brody (2004) - Longitudinal links among parenting, self-presentations to peers, and the development of externalizing and internalizing symptoms in African American siblings | EXCLUDE on study design |
| Brody (2005) - Protective Longitudinal Paths Linking Child Competence to Behavioral Problems Among African American Siblings | EXCLUDE on study design |
| Bubonya (2019) - The Great Recession and Children’s Mental Health in Australia | EXCLUDE on risk factor |
| Buchanan (2009) - Are there Shared Environmental Influences on Adolescent behavior? Evidence from a Study of Adoptive Siblings | EXCLUDE on study design |
| Bullock (2002) - Adult Sibling Expressed Emotion and Fellow Sibling Deviance: A New Piece of the Family Process Puzzle | EXCLUDE on study design |
| Burt (2005) - How are parent–child conflict and childhood externalizing symptoms related over time? Results from a genetically informative cross-lagged study | EXCLUDE on study design |
| Burt (2006) - Parental Divorce and Adolescent Delinquency: Ruling Out the Impact of Common Genes | EXCLUDE on risk factor |
| Burt (2007) - Environmental Contributions to Adolescent Delinquency: A Fresh Look at the Shared Environment | EXCLUDE on study design |
| Burt (2008) - How are parent-child conflict and childhood externalizing symptoms related over time? Results, from a genetically informative cross-lagged study | EXCLUDE on risk factor |
| Burt (2009) - Nonshared environmental mediation of the association between deviant peer affiliation and adolescent externalizing behaviors over time: Results from a cross-lagged monozygotic twin differences design | EXCLUDE on risk factor |
| Button (2008) - Parental punitive discipline, negative life events and gene–environment interplay in the development of externalizing behavior | EXCLUDE on study design |
| Carone (2018) - Italian Gay Father Families Formed by Surrogacy: Parenting, Stigmatization, and Children’s Psychological Adjustment | EXCLUDE on study design |
| Cecil (2018) - DRD4 methylation as a potential biomarker for physical aggression: An epigenome-wide, cross-tissue investigation | EXCLUDE on risk factor |
| Chao (2017) - The Causal Role of Alcohol Use in Adolescent Externalizing and Internalizing Problems: A Mendelian Randomization Study | EXCLUDE on risk factor |
| Chastang (2015) - Postnatal Environmental Tobacco Smoke Exposure Related to Behavioral Problems in Children | EXCLUDE on study design |
| Chatterji (2014) - Fetal Growth and Neurobehavioral Outcomes in Childhood | EXCLUDE on study design |
| Chen (2012) - Hostile Attributional Bias, Negative Emotional Responding, and Aggression in Adults: Moderating Effects of Gender and Impulsivity | EXCLUDE on study design |
| Chen (2019) - Associations between early life parent-child separation and shortened T telomere length and psychopathological outcomes during adolescence | EXCLUDE on study design |
| Cheung (2011) - Understanding contextual effects on externalizing behaviors in children in out-of-home care: Influence of workers and foster families | EXCLUDE on study design |
| Chien (2012) - The Younger Siblings of Childbearing Adolescents: Parenting Influences on Their Academic and Social-Emotional Adjustment | EXCLUDE on outcome |
| Choi (2019) - Maternal depression in the intergenerational transmission of childhood maltreatment and its sequelae: Testing postpartum effects in a longitudinal birth cohort | EXCLUDE on study design |
| Clark (2017) - Personality in the age of industry: Structure, heritability, and correlates of personality in middle childhood from the perspective of parents, teachers, and children | EXCLUDE on study design |
| Cluver (2009) - Parental illness, caregiving factors and psychological distress among children orphaned by acquired immune deficiency syndrome (AIDS) in South Africa | EXCLUDE on study design |
| Cole (2016) - The Trauma of Commercial Sexual Exploitation of Youth: A Comparison of CSE Victims to Sexual Abuse Victims in a Clinical Sample | EXCLUDE on risk factor |
| Coley (2013) - Timing, Extent, and Type of Child Care and Children’s Behavioral Functioning in Kindergarten | EXCLUDE on study design |
| Collishaw (2012) - Do Historical Changes in Parent–Child Relationships Explain Increases in Youth Conduct Problems? | EXCLUDE on study design |
| Compton (2003) - The contribution of parents and siblings to antisocial and depressive behavior in adolescents: A double jeopardy coercion model | EXCLUDE on study design |
| Coohey (2013) - Victimization, Parenting, and Externalizing Behavior Among Latino and White Adolescents | EXCLUDE on study design |
| Copp (2018) - Parental Incarceration and Child Well- being: Conceptual and Practical Concerns Regarding the Use of Propensity Scores | EXCLUDE on risk factor |
| Crea (2008) - Behavioral Outcomes for Substance-Exposed Adopted Children: Fourteen Years Postadoption | EXCLUDE on study design |
| Crea (2018) - Externalizing behaviors among adopted children: A longitudinal comparison of preadoptive childhood sexual abuse and other forms of maltreatment | EXCLUDE on study design |
| Crosby (2010) - A Tale of Two Methods: Comparing Regression and Instrumental Variables Estimates of the Effects of Preschool Child Care Type on the Subsequent Externalizing Behavior of Children in Low-Income Families | EXCLUDE on risk factor |
| D'Onofrio (2006) - A Genetically Informed Study of the Processes Underlying the Association Between Parental Marital Instability and Offspring Adjustment | EXCLUDE on outcome |
| D'Onofrio (2008) - Smoking during pregnancy and offspring externalizing problems: An exploration of genetic and environmental confounds | EXCLUDE on risk factor |
| D'Onofrio (2009a) - Maternal age at childbirth and offspring disruptive behaviors: Testing the causal hypothesis | EXCLUDE on risk factor |
| D'Onofrio (2009b) - A quasi-experimental analysis of the association between family income and offspring conduct problems | EXCLUDE on risk factor |
| D’Onofrio et al (2005) - A genetically informed study of marital instability and its association with offspring psychopathology | EXCLUDE on risk factor |
| D'Onofrio, Slutske (2007) - Intergenerational transmission of childhood conduct problems: A children of twins study | EXCLUDE on risk factor |
| D'Onofrio, Van Hulle (2007) - Causal Inferences Regarding Prenatal Alcohol Exposure and Childhood Externalizing Problems | EXCLUDE on risk factor |
| Dearing (2015) - Age of Entry Into Early Childhood Education and Care as a Predictor of Aggression: Faint and Fading Associations for Young Norwegian Children | EXCLUDE on outcome |
| Deater-Deckard (1996) - Within Family Variability in Parental Negativity and Control | EXCLUDE on study design |
| Deater-Deckard (1999) - An Adoption Study of the Etiology of Teacher and Parent Reports of Externalizing Behavior Problems in Middle Childhood | EXCLUDE on study design |
| Deater-Deckard (2002) - Sibling Relationships and Social-emotional Adjustment in Different Family Contexts | EXCLUDE on study design |
| Deater-Deckard (2006) - Maternal Warmth Moderates the Link Between Physical Punishment and Child Externalizing Problems: A Parent – Offspring Behavior Genetic Analysis | EXCLUDE on study design |
| Deater-Deckard (2009) - Conduct problems, IQ, and household chaos: a longitudinal multi-informant study | EXCLUDE on study design |
| Defoe (2013) - Siblings versus parents and friends: longitudinal linkages to adolescent externalizing problems | EXCLUDE on study design |
| Derks (2019) - Testing Bidirectional Associations Between Childhood Aggression and BMI: Results from Three Cohorts | EXCLUDE on study design |
| Derringer (2010) - Harsh Discipline, Childhood Sexual Assault, and MAOA Genotype: An Investigation of Main and Interactive Effects on Diverse Clinical Externalizing Outcomes | EXCLUDE on study design |
| Disney (2008) - Strengthening the Case: Prenatal Alcohol Exposure Is Associated With Increased Risk for Conduct Disorder | EXCLUDE on study design |
| Doi (2018) - Relationship Between Leaving Children at Home Alone and Their Mental Health: Results From the A-CHILD Study in Japan | EXCLUDE on risk factor |
| Doku (2009) - Parental HIV/AIDS status and death, and children's psychological wellbeing | EXCLUDE on study design |
| Dolan (2016) - Testing Causal Effects of Maternal Smoking During Pregnancy on Offspring’s Externalizing and Internalizing Behavior | EXCLUDE on study design |
| Dubois-Comtois (2015) - Behavior problems of children in foster care: Associations with foster mothers’ representations, commitment, and the quality of mother–child interaction | EXCLUDE on study design |
| Dumka (2010) - Parenting Self-Efficacy and Parenting Practices Over Time in Mexican American Families | EXCLUDE on study design |
| Dunifon (2003) - Maternal work behavior under welfare reform: how does the transition from welfare to work affect child development? | EXCLUDE on risk factor |
| Eaves (2010) - The mediating effect of parental neglect on adolescent and young adult anti-sociality: A longitudinal study of twins and their parents | EXCLUDE on study design |
| Edwards (2018) - The influence of child care on the behavior problems of children of teenage mothers | EXCLUDE on risk factor |
| Eisenberg (2010) - Relations among maternal socialization, effortful control, and maladjustment in early childhood | EXCLUDE on study design |
| Ekblad (2017) - Maternal Smoking During Pregnancy and the Risk of Psychiatric Morbidity in Singleton Sibling Pairs | EXCLUDE on risk factor |
| Ekblad (2019) - Disruptive Behavior in Siblings Discordant for Exposure to Maternal Smoking During Pregnancy: A Multi-rater Approach | EXCLUDE on risk factor |
| El Marroun (2019) - Preconception and prenatal cannabis use and the risk of behavioural and emotional problems in the offspring; a multi-informant prospective longitudinal study | EXCLUDE on study design |
| Elam (2014) - Adoptive Parent Hostility and Children’s Peer Behavior Problems: Examining the Role of Genetically Informed Child Attributes on Adoptive Parent Behavior | EXCLUDE on study design |
| Ellingson (2014) - A Sibling-Comparison Study of Smoking During Pregnancy and Childhood Psychological Traits | EXCLUDE on risk factor |
| Ellis (2012) - Smoking during pregnancy and psychiatric disorders in preschoolers | EXCLUDE on risk factor |
| Emery (2011) - Controlling for Selection Effects in the Relationship Between Child Behavior Problems and Exposure to Intimate Partner Violence | EXCLUDE on risk factor |
| Enns (2002) - Parental bonding and adult psychopathology: results from the US National Comorbidity Survey | EXCLUDE on study design |
| Estabrook (2016) - Separating Family-Level and Direct Exposure Effects of Smoking During Pregnancy on Offspring Externalizing Symptoms: Bridging the Behavior Genetic and Behavior Teratologic Divide | EXCLUDE on risk factor |
| Factor (2014) - Emotional Impulsivity in Children with ADHD Associated with Comorbid—Not ADHD—Symptomatology | EXCLUDE on outcome |
| Farr (2019) - Longitudinal Associations Between Coparenting and Child Adjustment Among Lesbian, Gay, and Heterosexual Adoptive Parent Families | EXCLUDE on study design |
| Feinberg (2007) - Parenting and Adolescent Antisocial Behavior and Depression Evidence of Genotype􏱚Parenting Environment Interaction | EXCLUDE on study design |
| Fergusson (1993) - The effect of maternal depression on maternal ratings of child behaviour | EXCLUDE on study design |
| Fergusson (1995) - The Adolescent Outcomes of Adoption: A 16 Year Longitudinal Study | EXCLUDE on risk factor |
| Fergusson (2003) - Deviant Peer Affiliations and Depression: Confounding or Causation? | EXCLUDE on study design |
| Finet (2021) - Adopted Children's Behavioral Adjustment Over Time: Pre-Adoption Experiences and Adoptive Parenting | EXCLUDE on study design |
| Fite (2006) - Childhood behavior problems and peer selection and socialization: Risk for adolescent alcohol use | EXCLUDE on study design |
| Fitzsimons (2019) - Father departure and children's mental health: How does timing matter? | EXCLUDE on risk factor |
| Flom (2020) - Longitudinal Links Between Callous-Unemotional Behaviors and Parenting in Early Childhood: A Genetically Informed Design | EXCLUDE on study design |
| Flouri (2018) - Maternal depressive symptoms in childhood and risky behaviours in early adolescence | EXCLUDE on study design |
| Flouri (2019) - Stressful life events, inflammation and emotional and behavioural problems in children: A population-based study | EXCLUDE on study design |
| Foley (2001) - Parental Concordance and Comorbidity for Psychiatric Disorder and Associate Risks for Current Psychiatric Symptoms and Disorders in a Community Sample of Juvenile Twins | EXCLUDE on study design |
| Gagnon-Oosterwaal (2012) - Pre-Adoption Adversity and Self-Reported Behavior Problems in 7 Year-Old International Adoptees | EXCLUDE on risk factor |
| Galboda-Liyanage (2003) - Mother ^ child joint activity and behaviour problems of pre-school children | EXCLUDE on study design |
| Gamble (2011) - Adolescent Sibling Relationship Quality and Adjustment: Sibling Trustworthiness and Modeling, as Factors Directly and Indirectly Influencing These Associationssode_5 | EXCLUDE on study design |
| Garcia (2000) - Destructive Sibling Conflict and the Development of Conduct Problems in Young Boys | EXCLUDE on study design |
| Gass (2007) - Are sibling relationships protective? A longitudinal study | EXCLUDE on study design |
| Gaysina, Fergusson (2013) - Maternal Smoking During Pregnancy and Offspring Conduct Problems Evidence From 3 Independent Genetically Sensitive Research Designs | EXCLUDE on risk factor |
| Ge (1996) - The developmental interface between nature and nurture: A mutual influence model of child antisocial behavior and parent behaviors | EXCLUDE on study design |
| Gershoff (2018) - Strengthening Causal Estimates for Links Between Spanking and Children’s Externalizing Behavior Problems | EXCLUDE on risk factor |
| Gest (1993) - Parenting quality, adversity, and conduct problems in adolescence: Testing process-oriented models of resilience | EXCLUDE on study design |
| Gibson (2018) - An Individual Growth Model Analysis of Childhood Spanking on Change in Externalizing Behaviors During Adolescence: A Comparison of Whites and African Americans Over a 12-Year Period | EXCLUDE on study design |
| Girard (2018) - Breastfeeding and externalising problems: a quasi‐experimental design with a national cohort | EXCLUDE on risk factor |
| Girard (2019) - Breastfeeding and behavioural problems: Propensity score matching with a national cohort of infants in Chile | EXCLUDE on risk factor |
| Gjerde (2017) - Maternal perinatal and concurrent depressive symptoms and child behavior problems: a sibling comparison study | EXCLUDE on risk factor |
| Gjerde (2020) - Maternal Perinatal and Concurrent Anxiety and Mental Health Problems in Early Childhood: A Sibling-Comparison Study | EXCLUDE on risk factor |
| Gjone (1995) - Parental Ratings of Behaviour Problems: A Twin and General Population Comparison | EXCLUDE on study design |
| Goldberg (2014) - Parents’ Relationship Quality and Children’s Behavior in Stable Married and Cohabiting Families | EXCLUDE on risk factor |
| Goldman (2011) - Direct and modifying influences of selected risk factors on children's pre-adoption functioning and post-adoption adjustment | EXCLUDE on study design |
| Golombok (2014) - Adoptive Gay Father Families: Parent–Child Relationships and Children’s Psychological Adjustment | EXCLUDE on study design |
| Goodnight (2012) - A Quasi-Experimental Analysis of the Influence of Neighborhood Disadvantage on Child and Adolescent Conduct Problems | EXCLUDE on study design |
| Grabow (2017) - Using an adoption–biological family design to examine associations between maternal trauma, maternal depressive symptoms, and child internalizing and externalizing behaviors | EXCLUDE on risk factor |
| Grotevant (2006) - Antisocial Behavior of Adoptees and Nonadoptees: Prediction from Early History and Adolescent Relationships | EXCLUDE on study design |
| Grotevant (2011) - Post-adoption contact, adoption communicative openness, and satisfaction with contact as predictors of externalizing behavior in adolescence and emerging adulthood | EXCLUDE on risk factor |
| Haber (2005) - Paternal Alcoholism and Offspring Conduct Disorder: Evidence for the ‘Common Genes’ Hypothesis | EXCLUDE on risk factor |
| Haber (2010) - Effect of paternal alcohol and drug dependence on offspring conduct disorder: Gene-environment interplay | EXCLUDE on risk factor |
| Hagekull (1992) - Prevalence of problematic behaviors in four-year-olds | EXCLUDE on study design |
| Hails (2019) - Interaction between adoptive mothers’ and fathers’ depressive symptoms in risk for children’s emerging problem behavior | EXCLUDE on risk factor |
| Halligan (2013) - The longitudinal development of emotion regulation capacities in children at risk for externalizing disorders | EXCLUDE on study design |
| Hannigan (2018) - Maternal prenatal depressive symptoms and risk for early-life psychopathology in offspring: genetic analyses in the Norwegian Mother and Child Birth Cohort Study | EXCLUDE on risk factor |
| Harding (2015) - Increases in Maternal Education and Low-Income Children’s Cognitive and Behavioral Outcomes | EXCLUDE on risk factor |
| Harold (1997) - Marital conflict and adolescent distress: the role of adolescent awareness | EXCLUDE on study design |
| Harold (2011) - Familial transmission of depression and antisocial behavior symptoms: disentangling the contribution of inherited and environmental factors and testing the mediating role of parenting | EXCLUDE on risk factor |
| Harris (2018) - Prenatal triptan exposure and neurodevelopmental outcomes in 5-year-old children: Follow-up from the Norwegian Mother and Child Cohort Study | EXCLUDE on risk factor |
| Harris-Waller (2016) - A comparison of parenting stress and children’s internalising, externalising and attachment-related behaviour difficulties in UK adoptive and non-adoptive families | EXCLUDE on risk factor |
| Hein (2017) - Psychological and Sociocultural Adaptation of Children Adopted from Russia and their Associations with Pre-Adoption Risk Factors and Parenting | EXCLUDE on risk factor |
| Herbst (2016) - The Impact of Child-Care Subsidies on Child Development: Evidence from Geographic Variation in the Distance to Social Service Agencies | EXCLUDE on risk factor |
| Hicks (2009) - Environmental Adversity and Increasing Genetic Risk for Externalizing Disorders | EXCLUDE on study design |
| Hicks (2013) - Genetic and Environmental Influences on the Familial Transmission of Externalizing Disorders in Adoptive and Twin Offspring | EXCLUDE on study design |
| Hinton (2019) - White matter microstructure correlates of general and specific second-order factors of psychopathology | EXCLUDE on study design |
| Hipwell (2016) - Effects of Adolescent Childbearing on Maternal Depression and Problem Behaviors: A Prospective, Population-Based Study Using Risk-Set Propensity Scores | EXCLUDE on risk factor |
| Hong (2011) - Personality Vulnerabilities to Psychopathology: Relations Between Trait Structure and Affective-Cognitive Processes | EXCLUDE on study design |
| Horn (2013) - Accounting for the Physical and Mental Health Benefits of Entry Into Marriage: A Genetically Informed Study of Selection and Causation | EXCLUDE on study design |
| Humphrey (2017) - Spatio-temporal neighborhood impacts on internalizing and externalizing behaviors in U.S. elementary school children: Effect modification by child and family socio-demographics | EXCLUDE on risk factor |
| Hussong (2007) - Externalizing Symptoms Among Children of Alcoholic Parents: Entry Points for an Antisocial Pathway to Alcoholism | EXCLUDE on study design |
| Ichikawa (2017) - It takes a village: Fixed-effects analysis of neighborhood collective efficacy and children's development | EXCLUDE on risk factor |
| Irons (2007) - Mendelian randomization: A novel test of the gateway hypothesis and models of gene–environment interplay | EXCLUDE on study design |
| Jackson (2016) - Breastfeeding duration and offspring conduct problems: The moderating role of genetic risk | EXCLUDE on study design |
| Jackson (2018) - Does TV viewing during toddlerhood predict social difficulties and conduct problems? | EXCLUDE on risk factor |
| Jaffee (2003) - Life with (or without) father: The benefits of living with two biological parents depend on the father's antisocial behavior | EXCLUDE on study design |
| Jaffee (2007) - Individual, family, and neighborhood factors distinguish resilient from non-resilient maltreated children: A cumulative stressors model | EXCLUDE on study design |
| Jaffee (2011) - Effects of Nonmaternal Care in the First 3 Years on Children’s Academic Skills and Behavioral Functioning in Childhood and Early Adolescence: A Sibling Comparison Study | EXCLUDE on risk factor |
| Jaffee (2012) - Chaotic Homes and Children’s Disruptive Behavior: A Longitudinal Cross-Lagged Twin Study | EXCLUDE on study design |
| Jansen (2018) - Polygenic scores for schizophrenia and educational attainment are associated with behavioural problems in early childhood in the general population | EXCLUDE on study design |
| Janssens (2017) - Adolescent externalizing behaviour, psychological control, and peer rejection: Transactional links and dopaminergic moderation | EXCLUDE on study design |
| Jeannin (2015) - Associations Between Direct and Indirect Perceptions of Parental Differential Treatment and Child Socio-Emotional Adaptation | EXCLUDE on study design |
| Jimenez-Etcheverria (2021) - Psychological adjustment, attachment difficulties, and perceptions of family relationships in adopted and institution-reared children: The case of Chile | EXCLUDE on risk factor |
| Jonson-Reid (2010) - The Effects of Child Maltreatment and Inherited Liability on Antisocial Development: An Official Records Study | EXCLUDE on study design |
| Kandel (1994) - Impact of Maternal Drug Use and Life Experiences on Preadolescent Children Born to Teenage Mothers | EXCLUDE on study design |
| Kaufman (2017) - Children’s Emotion Regulation Difficulties Mediate the Association Between Maternal Borderline and Antisocial Symptoms and Youth Behavior Problems Over 1 Year | EXCLUDE on study design |
| Keijsers (2016) - Parental monitoring and adolescent problem behaviors: How much do we really know? | EXCLUDE on publication type |
| Kendler (2008) - A genetically informative developmental study of the relationship between conduct disorder and peer deviance in males | EXCLUDE on study design |
| Kendler (2014) - A Swedish national adoption study of criminality | EXCLUDE on outcome |
| Kendler (2017) - The joint impact of cognitive performance in adolescence and familial cognitive aptitude on risk for major psychiatric disorders: a delineation of four potential pathways to illness | EXCLUDE on outcome |
| Kendler (2018) - Familial transmission of externalizing syndromes in extended Swedish families | EXCLUDE on outcome |
| Kendler (2020) - The causal nature of the association between resting pulse in late adolescence and risk for internalizing and externalizing disorders: a co-relative analysis in a national male Swedish sample | EXCLUDE on outcome |
| Kerr (2013) - Influences of Biological and Adoptive Mothers’ Depression and Antisocial Behavior on Adoptees’ Early Behavior Trajectories | EXCLUDE on risk factor |
| Keyes, Legrand (2008) - Parent Smoking and Adolescent Problem Behavior: An Adoption Study of General and Specific Effects | EXCLUDE on risk factor |
| Keyes, Sharma (2008) - The Mental Health of US Adolescents Adopted in Infancy | EXCLUDE on risk factor |
| Kim (1999) - Associations among Family Relationships, Antisocial Peers, and Adolescents' Externalizing Behaviors: Gender and Family Type Differences | EXCLUDE on study design |
| Kim (2020) - Paternal Influence on the Developmental Pathways of Maternal Parenting Stress, Home Learning Stimulation, and Children's Social Skills in the US and Korea: A Moderated Mediation Model | EXCLUDE on outcome |
| Kim-Cohen (2006) - The Caregiving Environments Provided to Children by Depressed Mothers With or Without an Antisocial History | EXCLUDE on study design |
| King (2009) - Parental alcohol dependence and the transmission of adolescent behavioral disinhibition: a study of adoptive and non-adoptive families | EXCLUDE on risk factor |
| King (2018) - Food insecurity and child behavior problems in fragile families | EXCLUDE on risk factor |
| Kinner (2007) - Do paternal arrest and imprisonment lead to child behaviour problems and substance use? A longitudinal analysis | EXCLUDE on study design |
| Kirk (2014) - The Acute and Enduring Consequences of Exposure to Violence on Youth Mental Health and Aggression | EXCLUDE on study design |
| Klahr (2014) - The etiology of the association between child antisocial behavior and maternal negativity varies across aggressive and non-aggressive rule-breaking forms of antisocial behavior | EXCLUDE on study design |
| Knoblach (2020) - The Association Between Genetic Predisposition and Parental Socialization: An Examination of Gene– Environment Correlations Using an Adoption-Based Design | EXCLUDE on outcome |
| Knopik (2009) - Genetic and environmental influences on externalizing behavior and alcohol problems in adolescence: A female twin study | EXCLUDE on study design |
| Knudsen (2015) - Maternal heavy alcohol use and toddler behavior problems: a fixed effects regression analysis | EXCLUDE on risk factor |
| Kollins (2009) - Effects of Postnatal Parental Smoking on Parent and Teacher Ratings of ADHD and Oppositional Symptoms | EXCLUDE on outcome |
| Koss (2020) - Early adversity and children's regulatory deficits: Does postadoption parenting facilitate recovery in postinstitutionalized children? | EXCLUDE on outcome |
| Kowal (2002) - Children’s Perceptions of the Fairness of Parental Preferential Treatment and Their Socioemotional Well-Being | EXCLUDE on study design |
| Ksinan (2020) - The Associations of Polygenic Scores for Risky Behaviors and Parenting Behaviors with Adolescent Externalizing Problems | EXCLUDE on study design |
| Kuijpers (2015) - Child Self-report to Identify Internalizing and Externalizing Problems and the Influence of Maternal Mental Health | EXCLUDE on study design |
| Kuja-Halkola (2014) - Maternal Smoking During Pregnancy and Adverse Outcomes in Offspring: Genetic and Environmental Sources of Covariance | EXCLUDE on outcome |
| Kuja-Halkola (2015) - Codevelopment of ADHD and externalizing behavior from childhood to adulthood | EXCLUDE on study design |
| Lahey (2009) - Are Oppositional-Defiant and Hyperactive–Inattentive Symptoms Developmental Precursors to Conduct Problems in Late Childhood?: Genetic and Environmental Links | EXCLUDE on study design |
| Lansford (2006) - Trajectories of internalizing, externalizing, and grades for children who have and have not experienced their parents' divorce or separation | EXCLUDE on study design |
| Larsson (2000) - Parents’ perception of mental development and behavioural problems in 8 to 9-year-old children | EXCLUDE on study design |
| Larsson, Viding, Plomin (2008) - Callous–Unemotional Traits and Antisocial Behavior Genetic, Environmental, and Early Parenting Characteristics | EXCLUDE on study design |
| Larsson, Viding, Rijsdijk, Plomin (2008) - Relationships Between Parental Negativity and Childhood Antisocial Behavior over Time: A Bidirectional Effects Model in a Longitudinal Genetically Informative Design | EXCLUDE on study design |
| Laurent (2014) - Stress System Development From Age 4.5 to 6: Family Environment Predictors and Adjustment Implications of HPA Activity Stability Versus Change | EXCLUDE on study design |
| Lee (2017) - Corporal Punishment and Child Aggression: Ethnic-Level Family Cohesion as a Moderator | EXCLUDE on study design |
| Lee (2018) - Out-of-School Time and Behaviors During Adolescence | EXCLUDE on risk factor |
| Leve (2009) - Structured Parenting of Toddlers at High Versus Low Genetic Risk: Two Pathways to Child Problems | EXCLUDE on timing of measures |
| Levy (1996) - Twin-sibling differences in parental reports of ADHD, speech, reading and behavioural problems | EXCLUDE on risk factor |
| Li (2007) - Risk and protective factors for urban African-American youth | EXCLUDE on study design |
| Li (2014) - Preadoption adversity, MAOA, and behavioral adjustment in internationally adopted Chinese girls | EXCLUDE on study design |
| Li (2019) - Assessing phenotypic and polygenic models of ADHD to identify mechanisms of risk for longitudinal trajectories of externalizing behaviors | EXCLUDE on study design |
| Lien (2016) - Perfluoroalkyl substances in cord blood and attention deficit/ hyperactivity disorder symptoms in seven-year-old children | EXCLUDE on study design |
| Linares (2006) - Mental Health Outcomes of Cocaine-Exposed Children at 6 Years of Age | EXCLUDE on study design |
| Liu (2018) - Developmental Patterns of Anger from Infancy to Middle Childhood Predict Problem Behaviors at Age 8 | EXCLUDE on study design |
| Loehlin (2010) - Parent-child closeness studied in adoptive families | EXCLUDE on study design |
| Long (2018) - The role of parent and offspring sex on risk for externalizing psychopathology in offspring with parental alcohol use disorder: A national Swedish study | EXCLUDE on outcome |
| Lund (2019) - Is the association between maternal alcohol consumption in pregnancy and pre-school child behavioural and emotional problems causal? Multiple approaches for controlling unmeasured confounding | EXCLUDE on risk factor |
| Luo (2020) - Parent–Child Discrepancies in Perceived Parental Favoritism: Associations with Children’s Internalizing and Externalizing Problems in Chinese Families | EXCLUDE on risk factor |
| Ma (2018) - Associations of neighborhood disorganization and maternal spanking with children’s aggression: A fixed-effects regression analysis | EXCLUDE on risk factor |
| Ma (2020) - Does community violence exposure moderate the associations between maternal spanking and early child behavior problems? | EXCLUDE on risk factor |
| Mabbe (2020) - Is Autonomy-Supportive Parenting Beneficial Only to Adolescents With an Autonomous Personality? Two Meanings of Goodness of Fit | EXCLUDE on outcome |
| MacKenzie (2011) - A cumulative ecological–transactional risk model of child maltreatment and behavioral outcomes: Reconceptualizing early maltreatment report as risk factor | EXCLUDE on study design |
| Madrid-Valero (2020) - Association between symptoms of sleep apnea and problem behaviors in young adult twins and siblings | EXCLUDE on study design |
| Maes (2007) - Genetic and Cultural Transmission of Antisocial Behavior: An Extended Twin Parent Model | EXCLUDE on study design |
| Mahoney (2003) - Marital and Severe Parent-to-Adolescent Physical Aggression in Clinic-Referred Families: Mother and Adolescent Reports on Co-occurrence and Links to Child Behavior Problems | EXCLUDE on study design |
| Malanchini (2019) - Aggressive behaviour in childhood and adolescence: the role of smoking during pregnancy, evidence from four twin cohorts in the EU-ACTION consortium | EXCLUDE on study design |
| Marceau (2013a) - Gene–Environment Correlation Underlying the Association Between Parental Negativity and Adolescent Externalizing Problems | EXCLUDE on study design |
| Marceau (2015a) - Combined influences of genes, prenatal environment, cortisol, and parenting on the development of children’s internalizing vs. externalizing problems | EXCLUDE on study design |
| Marceau (2015b) - Parental Knowledge is an Environmental Influence on Adolescent Externalizing | EXCLUDE on study design |
| Marceau (2019) - Parenting and Prenatal Risk as Moderators of Genetic Influences on Conduct Problems During Middle Childhood | EXCLUDE on study design |
| Markowitz (2016) - Father Absence and Adolescent Depression and Delinquency: A Comparison of Siblings Approach | EXCLUDE on outcome |
| Marmorstein (2009) - Alcohol and illicit drug dependence among parents: associations with offspring externalizing disorders | EXCLUDE on study design |
| Marmorstein, Iacono (2004) - Major depression and conduct disorder in youth: associations with parental psychopathology and parent-child conflict | EXCLUDE on study design |
| Marmorstein, Malone (2004) - Psychiatric Disorders Among Offspring of Depressed Mothers: Associations With Paternal Psychopathology | EXCLUDE on study design |
| McAdams (2015) - The relationship between parental depressive symptoms and offspring psychopathology: evidence from a children-of-twins study and an adoption study | EXCLUDE on risk factor |
| McCartney (2010) - Testing a Series of Causal Propositions Relating Time in Child Care to Children’s Externalizing Behavior | EXCLUDE on study design |
| McClowry (1994) - The Effects of Child Temperament, Maternal Characteristics, and Family Circumstances on the Maladjustment of School-Age Children | EXCLUDE on study design |
| McCrory (2012) - Prenatal Exposure to Maternal Smoking and Childhood Behavioural Problems: A Quasi-experimental Approach | EXCLUDE on risk factor |
| McCutcheon (2013) - Parent, sibling and peer associations with subtypes of psychiatric and substance use disorder comorbidity in offspring | EXCLUDE on study design |
| McGee (2000) - A longitudinal study of cannabis use and mental health from adolescence to early adulthood | EXCLUDE on study design |
| McGuire (1995) - Maternal differential treatment of siblings and children's behavioral problems: A longitudinal study | EXCLUDE on study design |
| McLaughlin (2011) - Caregiver and self-report of mental health symptoms in 9-year old children with prenatal cocaine exposure | EXCLUDE on study design |
| Melancon (2019) - Parenting Stress of Adoptive Mothers, Mother–Child Conflict, and Behavior Problems During Adolescence Among International Adoptees | EXCLUDE on study design |
| Mesman (2009) - Predicting Growth Curves of Early Childhood Externalizing Problems: Differential Susceptibility of Children with Difficult Temperament | EXCLUDE on study design |
| Messerlian (2017) - Paternal and maternal preconception urinary phthalate metabolite concentrations and child behavior | EXCLUDE on study design |
| Meunier (2011) - Externalizing behavior trajectories: The role of parenting, sibling relationships and child personality | EXCLUDE on study design |
| Meunier (2013) - Multilevel Mediation: Cumulative Contextual Risk, Maternal Differential Treatment, and Children’s Behavior Within Families | EXCLUDE on study design |
| Meunier, Roskam (2012) - Parental differential treatment, child’s externalizing behavior and sibling relationships: Bridging links with child’s perception of favoritism and personality, and parents’ self-efficacy | EXCLUDE on study design |
| Meunier, Wade (2012) - Mothers’ differential parenting and children’s behavioural outcomes: Exploring the moderating role of family and social context | EXCLUDE on study design |
| Miller (2009) - Outcomes of children adopted from Eastern Europe | EXCLUDE on study design |
| Min (2018) - Developmental trajectories of externalizing behavior from ages 4 to 12: Prenatal cocaine exposure and adolescent correlates | EXCLUDE on study design |
| Min, Minnes, Lang (2014) - Externalizing behavior and substance use related problems at 15 years in prenatally cocaine exposed adolescents | EXCLUDE on study design |
| Min, Minnes, Yoon (2014) - Self-Reported Adolescent Behavioral Adjustment: Effects of Prenatal Cocaine Exposure | EXCLUDE on study design |
| Minnes (2010) - The effects of prenatal cocaine exposure on problem behavior in children 4–10 years | EXCLUDE on study design |
| Moffitt (1987) - Parental mental disorder and offspring criminal behaviour: an adoption study | EXCLUDE on outcome |
| Mollegaard (2020) - The effect of birth weight on behavioral problems in early adolescence: New evidence from monozygotic twins | EXCLUDE on risk factor |
| Morgan (2010) - A Propensity Score Matching Analysis of the Effects of Special Education Services | EXCLUDE on outcome |
| Moroney (2017) - Externalizing Outcomes of Youth with and without ADHD: Time-Varying Prediction by Parental ADHD and Mediated Effects | EXCLUDE on study design |
| Moss (1995) - Prepubertal sons of substance abusers: influences of parental and familial substance abuse on behavioural disposition, iq and school achievement | EXCLUDE on study design |
| Mostafa (2018) - The Impact of Complex Family Structure on Child Well-being: Evidence From Siblings | EXCLUDE on risk factor |
| Mullola (2021) - Early childhood psychosocial family risks and cumulative dopaminergic sensitizing score: Links to behavior problems in US 9-year-olds | EXCLUDE on study design |
| Muniz (2019) - The effects of adverse childhood experiences on internalising versus externalising outcomes | EXCLUDE on study design |
| Nadeem (2017) - Long-Term Effects of Pre-Placement Risk Factors on Children’s Psychological Symptoms and Parenting Stress Among Families Adopting Children From Foster Care | EXCLUDE on study design |
| Narusyte (2007) - Aggression as a mediator of genetic contributions to the association between negative parent–child relationships and adolescent antisocial behavior | EXCLUDE on study design |
| Natsuaki (2009) - Aggressive Behavior between Siblings and the Development of Externalizing Problems: Evidence from a Genetically Sensitive Study | EXCLUDE on study design |
| Nawa (2021) - Oxytocin Response Following Playful Mother-Child Interaction in Survivors of the Great East Japan Earthquake | EXCLUDE on study design |
| Neiderhiser (1999) - Relationships Between Parenting and Adolescent Adjustment Over Time: Genetic and Environmental Contributions | EXCLUDE on study design |
| Neiderhiser (2016) - Estimating the Roles of Genetic Risk, Perinatal Risk, and Marital Hostility on Early Childhood Adjustment: Medical Records and Self-Reports | EXCLUDE on risk factor |
| Nulman (2015) - Neurodevelopment of Children Prenatally Exposed to Selective Reuptake Inhibitor Antidepressants: Toronto Sibling Study | EXCLUDE on risk factor |
| O'Connor (1998) - Genotype-environment correlations in late childhood and early adolescence: Antisocial behavioral problems and coercive parenting | EXCLUDE on timing of measures |
| Obsuth (2017) - A Non-bipartite Propensity Score Analysis of the Effects of Teacher–Student Relationships on Adolescent Problem and Prosocial Behavior | EXCLUDE on risk factor |
| Odgers (2012) - Supportive parenting mediates neighborhood socioeconomic disparities in children’s antisocial behavior from ages 5 to 12 | EXCLUDE on study design |
| Odgers (2017) - Violence exposure is associated with adolescents’ same- and next-day mental health symptoms | EXCLUDE on study design |
| Odudu (2020) - Associations Between Domain Differentiated Sibling Conflict and Adolescent Problem Behavior | EXCLUDE on study design |
| Oliver (2008) - Nonshared environmental influences on teacher-reported behaviour problems: monozygotic twin differences in perceptions of the classroom | EXCLUDE on risk factor |
| Oliver (2018) - Mother-Child Positivity and Negativity: Family-Wide and Child-Specific Main Effects and Interactions Predict Child Adjustment | EXCLUDE on study design |
| Olson (2013) - Deconstructing the externalizing spectrum: Growth patterns of overt aggression, covert aggression, oppositional behavior, impulsivity/inattention, and emotion dysregulation between school entry and early adolescence | EXCLUDE on study design |
| Olson (2020) - Development of Externalizing Symptoms Across the Toddler Period: The Critical Role of Older Siblings | EXCLUDE on study design |
| Orlebeke (1997) - Increase in child behaviour problems resulting from maternal smoking during pregnancy | EXCLUDE on study design |
| Orlebeke (1998) - Frequency of Parental Report of Problem Behavior in Children Decreases with Increasing Maternal Age at Delivery | EXCLUDE on study design |
| Orlebeke (1999) - Child behavior problems increased by maternal smoking during pregnancy | EXCLUDE on study design |
| Orri (2019) - Early childhood child care and disruptive behavior problems during adolescence: a 17-year population-based propensity score study | EXCLUDE on risk factor |
| Padilla (2020) - Infant temperament, parenting and behavior problems: Variation by parental education and income | EXCLUDE on study design |
| Palmer (2007) - Childhood Experiences of Parenting and Causal Attributions for Criminal Behavior Among Young Offenders and Non-Offenders | EXCLUDE on study design |
| Palmer (2016) - Effects of Maternal Smoking during Pregnancy on Offspring Externalizing Problems: Contextual Effects in a Sample of Female Twins | EXCLUDE on risk factor |
| Paradis (2011) - Maternal smoking during pregnancy and criminal offending among adult offspring | EXCLUDE on outcome |
| Paradis (2017) - Maternal smoking during pregnancy and offspring antisocial behaviour: findings from a longitudinal investigation of discordant siblings | EXCLUDE on risk factor |
| Parker (2019) - Maternal acetaminophen use during pregnancy and childhood behavioural problems: Discrepancies between mother‐ and teacher‐reported outcomes | EXCLUDE on study design |
| Pemberton (2010) - Influence of parental depressive symptoms on adopted toddler behaviors: An emerging developmental cascade of genetic and environmental effects | EXCLUDE on risk factor |
| Perry (2018) - Concurrent child history and contextual predictors of children's internalizing and externalizing behavior problems in foster care | EXCLUDE on study design |
| Petkovsek (2014) - Prenatal smoking and genetic risk: Examining the childhood origins of externalizing behavioral problems | EXCLUDE on study design |
| Pike (2017) - Child Behavior and Sibling Relationship Quality: A Cross-Lagged Analysis | EXCLUDE on study design |
| Powers (2016) - Restrictive educational placements increase adolescent risks for students with early-starting conduct problems | EXCLUDE on risk factor |
| Price (2013) - Predictors of Externalizing Behavior Problems in Early Elementary-Aged Children: The Role of Family and Home Environments | EXCLUDE on study design |
| Rajyaguru (2019) - Disciplinary Parenting Practice and Child Mental Health: Evidence From the UK Millennium Cohort Study | EXCLUDE on study design |
| Ramanathan (2017) - Familial transient financial difficulties during infancy and long-term developmental concerns | EXCLUDE on risk factor |
| Ramchandani (2008) - The effects of pre- and postnatal depression in fathers: A natural experiment comparing the effects of exposure to depression on offspring | EXCLUDE on study design |
| Raudino (2013) - The intergenerational transmission of conduct problems | EXCLUDE on study design |
| Reiss (1995) - Genetic Questions for Environmental Studies Differential Parenting and Psychopathology in Adolescence | EXCLUDE on study design |
| Renner (2012) - Single Types of Family Violence Victimization and Externalizing Behaviors Among Children and Adolescents | EXCLUDE on study design |
| Rhee (2018) - The Association Between Toddlerhood Self-Control and Later Externalizing Problems | EXCLUDE on study design |
| Ribeiro (2019) - Peer Effects on Aggressive Behavior in Norwegian Child Care Centers | EXCLUDE on outcome |
| Richmond (2003) - Siblings’ Differential Experiences of Marital Conflict and Differences in Psychological Adjustment | EXCLUDE on risk factor |
| Richmond (2005) - Longitudinal Associations Between Sibling Relationship Quality, Parental Differential Treatment, and Children’s Adjustment | EXCLUDE on study design |
| Richmond (2008) - Longitudinal Associations Between Parents’ Hostility and Siblings’ Externalizing Behavior in the Context of Marital Discord | EXCLUDE on study design |
| Rivenbark (2019) - Perceived Social Status and Mental Health Among Young Adolescents: Evidence From Census Data to Cellphones | EXCLUDE on study design |
| Rivenbark (2020) - Adolescents’ perceptions of family social status correlate with health and life chances: A twin difference longitudinal cohort study | EXCLUDE on risk factor |
| Robbers (2011) - Pre-divorce problems in 3-year-olds: a prospective study in boys and girls | EXCLUDE on study design |
| Rodrigues (2017) - Behavioral Risk Assessment From Newborn to Preschool: The Value of Older Siblings | EXCLUDE on study design |
| Roisman (2012) - A Behavior-Genetic Study of the Legacy of Early Caregiving Experiences: Academic Skills, Social Competence, and Externalizing Behavior in Kindergarten | EXCLUDE on study design |
| Rolan (2018) - Individual and Sibling Characteristics: Parental Differential Treatment and Adolescent Externalizing Behaviors | EXCLUDE on study design |
| Rooney (2003) - Small for Gestational Age as a Predictor of Behavioral and Learning Problems in Twins | EXCLUDE on outcome |
| Roza (2009) - Maternal smoking during pregnancy and child behaviour problems: the Generation R Study | EXCLUDE on study design |
| Salvatore, Aliev (2015) - Polygenic risk for externalizing disorders: Gene-by-development and gene-by-environment effects in adolescents and young adults | EXCLUDE on study design |
| Salvatore, Meyers (2015) - Intergenerational Continuity in Parents’ and Adolescents’ Externalizing Problems: The Role of Life Events and Their Interaction With GABRA2 | EXCLUDE on study design |
| Samek (2013) - Gene-Environment Interplay between Parent-Child Relationship Problems and Externalizing Disorders in Adolescence and Young Adulthood | EXCLUDE on study design |
| Samek (2016) - Antisocial Peer Affiliation and Externalizing Disorders in the Transition From Adolescence to Young Adulthood: Selection Versus Socialization Effects | EXCLUDE on study design |
| Schaefer (2018) - Adolescent Victimization and Early-Adult Psychopathology: Approaching Causal Inference Using a Longitudinal Twin Study to Rule Out Noncausal Explanations | EXCLUDE on outcome |
| Schenck-Fontaine (2020) - Associations Between Perceived Material Deprivation, Parents’ Discipline Practices, and Children’s Behavior Problems: An International Perspective | EXCLUDE on study design |
| Schermerhorn (2011) - A Genetically Informed Study of Associations Between Family Functioning and Child Psychosocial Adjustment | EXCLUDE on risk factor |
| Schmitz (1999) - Temperament and Problem Behaviour during Early Childhood | EXCLUDE on study design |
| Scott (2014) - Early parental physical punishment and emotional and behavioural outcomes in preschool children | EXCLUDE on study design |
| Sehmi (2020) - Infant domestic adoption: outcomes at mid- | EXCLUDE on study design |
| Shapiro (2010) - Natural Experiment in Deviant Peer Exposure and Youth Recidivism | EXCLUDE on outcome |
| Shaw (2019) - Trajectories and Predictors of Children’s Early-Starting Conduct Problems: Child, Family, Genetic, and Intervention Effects | EXCLUDE on study design |
| Shek (2019) - Reciprocal Relationships Between Moral Competence and Externalizing Behavior in Junior Secondary Students: A Longitudinal Study in Hong Kong | EXCLUDE on study design |
| Shortt (2010) - Maternal emotion coaching, adolescent anger regulation, and siblings’ externalizing symptoms | EXCLUDE on study design |
| Sidor (2017) - The link between infant regulatory problems, temperament traits, maternal depressive symptoms and children’s psychopathological symptoms at age three: a longitudinal study in a German at-risk sample | EXCLUDE on study design |
| Silberg (2004) - Analysing the contributions of genes and parent–child interaction to childhood behavioural and emotional problems: a model for the children of twins | EXCLUDE on study design |
| Silberg (2010) - Genetic and environmental influences on the transmission of parental depression to children's depression and conduct disturbance: An extended children of twins study | EXCLUDE on study design |
| Silberg (2012) - Unraveling the effect of genes and environment in the transmission of parental antisocial behavior to children’s conduct disturbance, depression and hyperactivity | EXCLUDE on study design |
| Simmel (2007) - Risk and Protective Factors Contributing to the Longitudinal Psychosocial Well-Being of Adopted Foster Children | EXCLUDE on study design |
| Simons (2012) - Social Adversity, Genetic Variation, Street Code, and Aggression: A Genetically Informed Model of Violent Behavior | EXCLUDE on study design |
| Singh (2011) - Parental depression and offspring psychopathology: a Children of Twins study | EXCLUDE on risk factor |
| Skar (2014) - Evaluation of Follow-Up Effects of the International Child Development Programme on Caregivers in Mozambique | EXCLUDE on study design |
| Skopp (2005) - Siblings in Domestically Violent Families: Experiences of Interparent Conflict and Adjustment Problems | EXCLUDE on risk factor |
| Smith (2018) - Behavior problems in adolescence among international adoptees, pre-adoption adversity, and parenting stress | EXCLUDE on study design |
| Smorti (2020) - Parenting and Sibling Relationships in Family with Disruptive Behavior Disorders. Are Non-Clinical Siblings More Vulnerable for Emotional and Behavioral Problems? | EXCLUDE on study design |
| Solmeyer (2017) - Parents’ Differential Treatment of Adolescent Siblings in African American Families | EXCLUDE on study design |
| Staff (2019) - Changes in Externalizing Behaviors After Children First Have an Alcoholic Drink and First Drink Heavily | EXCLUDE on risk factor |
| Stanger (2004) - Parenting and Children’s Externalizing Problems in Substance-Abusing Families | EXCLUDE on study design |
| Stevenson (2019) - An examination of father vulnerability and coercive family process after the birth of a sibling: A spillover cascade model | EXCLUDE on study design |
| Stice (1995) - A Longitudinal Examination of the Reciprocal Relations Between Perceived Parenting and Adolescents' Substance Use and Externalizing Behaviors | EXCLUDE on study design |
| Stoolmiller (2001) - Synergistic Interaction of Child Manageability Problems and Parent-Discipline Tactics in Predicting Future Growth in Externalizing Behavior for Boys | EXCLUDE on study design |
| Stover (2012) - Fathering and mothering in the family system: linking marital hostility and aggression in adopted toddlers | EXCLUDE on study design |
| Su (2018) - Understanding Mechanisms of Genetic Risk for Adolescent Internalizing and Externalizing Problems: The Mediating Role of Parenting and Personality | EXCLUDE on study design |
| Sundbakk (2019) - Impact of prenatal exposure to benzodiazepines and z-hypnotics on behavioral problems at 5 years of age: A study from the Norwegian Mother and Child Cohort Study | EXCLUDE on risk factor |
| Tamrouti-Makkink (2004) - The relation between the absolute level of parenting and differential parental treatment with adolescent siblings’ adjustment | EXCLUDE on study design |
| Tan (2006) - Parental ratings of behavioral adjustment in two samples of adopted Chinese girls: Age-related versus socio-emotional correlates and predictors | EXCLUDE on study design |
| Tan (2015) - Non-Child-Related Family Stress, Parenting Styles, and Behavior Problems in School-Age Girls Adopted from China | EXCLUDE on study design |
| Taraban (2019) - Parental Depression, Overreactive Parenting, and Early Childhood Externalizing Problems: Moderation by Social Support | EXCLUDE on risk factor |
| Taylor (2019) - Characteristics of youth with reported family history of psychosis spectrum symptoms in the Philadelphia Neurodevelopmental Cohort | EXCLUDE on study design |
| Thompson (2003) - Authoritarian parenting attitudes as a risk for conduct problems Results from a British national cohort study | EXCLUDE on study design |
| Topitzes (2011) - Child Maltreatment and Offending Behavior: Gender-Specific Effects and Pathways | EXCLUDE on study design |
| Topitzes (2012) - From Child Maltreatment to Violent Offending: An Examination of Mixed-Gender and Gender-Specific Models | EXCLUDE on study design |
| Tore (2018) - The Association of Intrapair Birth-Weight Differences With Internalizing and Externalizing Behavior Problems | EXCLUDE on risk factor |
| Towers (2000) - Genetic and environmental in􏱡uences on teacher ratings of the Child Behavior Checklist | EXCLUDE on study design |
| Trentacosta (2019) - Callous-Unemotional Behaviors and Harsh Parenting: Reciprocal Associations across Early Childhood and Moderation by Inherited Risk | EXCLUDE on outcome |
| Tronnes (2019) - Prenatal paracetamol exposure and neurodevelopmental outcomes in preschool‐aged children | EXCLUDE on risk factor |
| Tully et al (2008) - An Adoption Study of Parental Depression as an Environmental Liability for Adolescent Depression and Childhood Disruptive Disorders | EXCLUDE on risk factor |
| Tung (2019) - Childhood maltreatment affects adolescent sensitivity to parenting and close friendships in predicting growth in externalizing behavior | EXCLUDE on study design |
| Turney (2012) - Pathways of disadvantage: Explaining the relationship between maternal depression and children’s problem behaviors | EXCLUDE on risk factor |
| Turney (2017) - The Unequal Consequences of Mass Incarceration for Children | EXCLUDE on risk factor |
| Tuvblad (2013) - Psychopathic Personality and Negative Parent-to-Child Affect: A Longitudinal Cross-lag Twin Study | EXCLUDE on study design |
| Tyrell (2019) - The unique effects of maternal and paternal depressive symptoms on youth's symptomatology: Moderation by family ethnicity, family structure, and child gender | EXCLUDE on study design |
| Van Beijsterveldt (2005) - Short- and Long-Term Effects of Child Care on Problem Behaviors in a Dutch Sample of Twins | EXCLUDE on study design |
| van der Pol (2009) - Sibling Gender Configuration and Family Processes | EXCLUDE on study design |
| van der Vegt (2009) - Early Childhood Adversities and Trajectories of Psychiatric Problems in Adoptees: Evidence for Long Lasting Effects | EXCLUDE on study design |
| Van der Voort (2013) - Delinquent and aggressive behaviors in early-adopted adolescents: Longitudinal predictions from child temperament and maternal sensitivity | EXCLUDE on study design |
| Van Lieshout (2011) - Canadian Youth Born Large or Small for Gestational Age and Externalising and Internalising Problems | EXCLUDE on study design |
| Van Lieshout (2013) - Maternal Pre-Pregnancy Body Mass Index and Internalizing and Externalizing Problems in Offspring | EXCLUDE on study design |
| Vaughan (2015) - Clarifying the Associations between Age at Menarche and Adolescent Emotional and Behavioral Problems | EXCLUDE on outcome |
| Veiga (2016) - Preschoolers’ free play - connections with emotional and social functioning | EXCLUDE on study design |
| Verbeek (2012) - Postpartum depression predicts offspring mental health problems in adolescence independently of parental lifetime psychopathology | EXCLUDE on study design |
| Verona (2005) - The Intergenerational Transmission of Externalizing Behaviors in Adult Participants: The Mediating Role of Childhood Abuse | EXCLUDE on study design |
| Vitaro (2016) - Links between friends’ physical aggression and adolescents’ physical aggression: What happens if gene-environment correlations are controlled? | EXCLUDE on outcome |
| Volling (1998) - Family Relationships and Children's Emotional Adjustment as Correlates of Maternal and Paternal Differential Treatment: A Replication with Toddler and Preschool Siblings | EXCLUDE on study design |
| Volling (2019) - Maternal and paternal trajectories of depressive symptoms predict family risk and children’s emotional and behavioral problems after the birth of a sibling | EXCLUDE on study design |
| Wagner (2009) - The Limited Effects of Obstetrical and Neonatal Complications on Conduct and Attention-Deficit Hyperactivity Disorder Symptoms in Middle Childhood | EXCLUDE on study design |
| Wagner (2020) - Less imitation of arbitrary actions is a specific developmental precursor to callous–unemotional traits in early childhood | EXCLUDE on study design |
| Waldron (2009) - Parental Alcoholism and Offspring Behavior Problems: Findings in Australian Children of Twins | EXCLUDE on risk factor |
| Wang (2017) - Roles of Response Inhibition and Gene-Environment Interplay in Pathways to Adolescents’ Externalizing Problems | EXCLUDE on study design |
| Wang (2018) - Language Delay and Externalizing Problems in Preschool Age: A Prospective Cohort Study | EXCLUDE on study design |
| Warner (2006) - Predicting Caregiver-Reported Behavior Problems in Cocaine-Exposed Children at 3 Years | EXCLUDE on study design |
| Weaver (2015) - Mediation and Moderation of Divorce Effects on Children’s Behavior Problems | EXCLUDE on risk factor |
| Weeks (2019) - Discrimination Matters: Relations of Perceived Discrimination to Student Mental Health | EXCLUDE on study design |
| Weinberg (2004) - The Minnesota Transracial Adoption Study: Parent Reports of Psychosocial Adjustment at Late Adolescence | EXCLUDE on study design |
| Welner (1988) - School-aged children of depressed parents: a blind and controlled study | EXCLUDE on study design |
| Wertz (2016) - Parental monitoring and knowledge: Testing bidirectional associations with youths’ antisocial behavior | EXCLUDE on study design |
| Wesseldijk (2018) - Do Parental Psychiatric Symptoms Predict Outcome in Children With Psychiatric Disorders? A Naturalistic Clinical Study | EXCLUDE on study design |
| Whiteman (2015) - Sibling Relationships and Adolescent Adjustment: Longitudinal Associations in Two-Parent African American Families | EXCLUDE on study design |
| Whiteman (2020) - Youth’s Sibling Relationships Across the Course of a Parent’s Military Deployment: Trajectories and Implications | EXCLUDE on study design |
| Whitney (2017) - The Effects of No Child Left Behind on Children’s Socioemotional Outcomes | EXCLUDE on outcome |
| Wichers (2001) - Associations between nonshared environment and child problem behaviour | EXCLUDE on study design |
| Williams (1998) - Maternal Cigarette Smoking and Child Psychiatric Morbidity: A Longitudinal Study | EXCLUDE on study design |
| Williams (2016) - Testing for Plausibly Causal Links Between Parental Bereavement and Child Socio-Emotional and Academic Outcomes: A Propensity-Score Matching Model | EXCLUDE on risk factor |
| Wolfe (2016) - The effects of maternal alcohol use disorders on childhood relationships and mental health | EXCLUDE on study design |
| Wong (2019) - Disentangling the Effects of Exposure to Maternal Substance Misuse and Physical Abuse and Neglect on Child Behavioral Problems | EXCLUDE on study design |
| Wu (2015) - Effects of kinship care on behavioral problems by child age: A propensity score analysis | EXCLUDE on risk factor |
| Yamaoka (2019) - Positive Parenting Matters in the Face of Early Adversity | EXCLUDE on study design |
| Yang, Fombonne (2011) - Duration of gestation, size at birth and later childhood behaviour | EXCLUDE on study design |
| Yang, Tilling (2011) - Pre-natal and post-natal growth trajectories and childhood cognitive ability and mental health | EXCLUDE on study design |
| Zachrisson (2013) - Little Evidence That Time in Child Care Causes Externalizing Problems During Early Childhood in Norway | EXCLUDE on risk factor |
| Zanoti-Jeronymo (2005) - Self-concept, academic performance and behavioral evaluation of the children of alcoholic parents | EXCLUDE on study design |
| Zhang (2020) - Coercive Parenting Mediates the Relationship between Military Fathers’ Emotion Regulation and children’s Adjustment | EXCLUDE on study design |
| Zhang (2020) - Beyond orchids and dandelions: Susceptibility to environmental influences is not bimodal | EXCLUDE on outcome |
| Zondervan-Zwijnenburg (2020) - Parental Age and Offspring Childhood Mental Health: A Multi-Cohort, Population-Based Investigation | EXCLUDE on study design |
| Zvara (2019) - Maternal history of childhood maltreatment and children’s cognitive and social development | EXCLUDE on risk factor |

### **Table S10.** Summary of all the effect sizes included in the meta-analyses of positive parenting practices.

| **Reference** | **Country** | **Method** | **Sample size** | **Sex** | **Risk factor** | **Maternal/**  **paternal** | **Informant** | ***Age^EXP^*** | **Outcome** | ***Age^OUT^*** | **Time** | **RoB** | **ES** | **SE** |
| --- | --- | --- | --- | --- | --- | --- | --- | --- | --- | --- | --- | --- | --- | --- |
| Anthony (2019) | UK | Adoption study | 62 | Mixed | High Parental Warmth | Combined | Caregiver/  caregiver | 2.33 | Externalising Behaviour | 3.66 | 1.33 | High quality | -0.220 | 0.136 |
| Asbury (2006) [11] | UK | Discordant twin study | 4,090 | Mixed (whole sample) | Effective Parent-Child Communication | Combined | Caregiver/  teacher | 4.00 | Conduct Problems | 7.00 | 3.00 | High quality | 0.080 | 0.041 |
| Asbury (2006) [12] | UK | Discordant twin study | 4,090 | Mixed (whole sample) | Effective Parent-Child Communication | Combined | Caregiver/  teacher | 4.00 | Conduct Problems | 7.00 | 3.00 | High quality | -0.010 | 0.022 |
| Barnett (2013) [1] | USA | Discordant sibling study | 274 | Mixed (whole sample) | High Parental Warmth | Maternal | Observer/  caregiver | 2.98 | Externalising Behaviour | 3.98 | 1.00 | High risk | -0.018 | 0.074 |
| Boisvert (2008) [1] | USA | Discordant sibling study | 578 | Mixed (whole sample) | High Parental Warmth | Combined | Caregiver/  caregiver | 12.15 | Externalising Behaviour | 17.15 | 5.00 | High quality | -0.046 | 0.051 |
| Boisvert (2008) [2] | USA | Discordant sibling study | 578 | Mixed (whole sample) | High Parental Monitoring | Combined | Caregiver/  caregiver | 12.15 | Externalising Behaviour | 17.15 | 5.00 | High quality | -0.116 | 0.048 |
| Boyle (2004) [4] | USA | Discordant sibling study | 7,392 | Mixed | High Parental Involvement | Maternal | Caregiver/  caregiver | 7.47 | Externalising Behaviour | 7.47 | 0.00 | High risk | 0.020 | 0.009 |
| Boyle (2004) [6] | USA | Discordant sibling study | 3,757 | Mixed | High Parental Involvement | Maternal | Teacher/  teacher | 7.47 | Externalising Behaviour | 7.47 | 0.00 | High risk | 0.040 | 0.016 |
| Boyle (2004) [8] | USA | Discordant sibling study | 2,876 | Mixed | High Parental Warmth | Maternal | Observer/  caregiver | 9.03 | Externalising Behaviour | 9.03 | 0.00 | High risk | -0.010 | 0.022 |
| Caspi (2004) [3] | UK | Discordant twin study | 1,212 | Mixed | Positive Parental Feeling | Maternal | Caregiver/  caregiver | 5.00 | Externalising Behaviour | 6.50 | 1.50 | High quality | -0.110 | 0.043 |
| Caspi (2004) [4] | UK | Discordant twin study | 1,212 | Mixed | High Parental Warmth | Maternal | Caregiver/  caregiver | 5.00 | Externalising Behaviour | 6.50 | 1.50 | High quality | -0.110 | 0.043 |
| Caspi (2004) [7] | UK | Discordant twin study | 1,126 | Mixed | High Parental Warmth | Maternal | Caregiver/  teacher | 5.00 | Externalising Behaviour | 6.50 | 1.50 | High quality | -0.120 | 0.047 |
| Caspi (2004) [8] | UK | Discordant twin study | 1,126 | Mixed | High Parental Warmth | Maternal | Caregiver/  teacher | 5.00 | Externalising Behaviour | 6.50 | 1.50 | High quality | -0.100 | 0.051 |
| Deater-Deckard (2004) | USA | Adoption study | 224 | Mixed | Positive Parent-Child Relationship | Maternal | Observer/  mixed | 8.16 | Conduct Problems | 8.16 | 0.00 | Very high risk | -0.390 | 0.118 |
| Glover (2010) [1] | USA | Adoption study | 85 | Mixed | Positive Parental Feeling | Maternal | Caregiver (M)/  caregiver (F) | 5.59 | Conduct Problems | 5.59 | 0.00 | Very high risk | -0.290 | 0.112 |
| Glover (2010) [3] | USA | Adoption study | 85 | Mixed | Positive Parental Feeling | Paternal | Caregiver (F)/  caregiver (M) | 5.59 | Conduct Problems | 5.59 | 0.00 | Very high risk | -0.380 | 0.115 |
| Hou (2013) [1] | China | Discordant twin study | 690 | Mixed | High Parental Warmth | Maternal | Mixed/  mixed | 13.86 | Conduct Problems | 15.36 | 1.50 | High quality | 0.050 | 0.054 |
| Hou (2013) [3] | China | Discordant twin study | 690 | Mixed | High Parental Warmth | Paternal | Mixed/  mixed | 13.86 | Conduct Problems | 15.36 | 1.50 | High quality | -0.080 | 0.054 |
| Mark (2017) [1] | UK | Discordant sibling study | 156 | Mixed | Positive Parent-Child Relationship | Maternal | Self/  caregiver | 10.94 | Externalising Behaviour | 10.94 | 0.00 | Very high risk | -0.049 | 0.117 |
| Meunier, Bisceglia (2012) [1] | Canada | Discordant sibling study | 809 | Mixed | Positive Parent-Child Relationship | Maternal | Caregiver/  caregiver | 4.49 | Oppositional Defiant Disorder | 4.49 | 0.00 | High risk | -0.310 | 0.044 |
| Meunier, Bisceglia (2012) [2] | Canada | Discordant sibling study | 809 | Mixed | Positive Parent-Child Relationship | Paternal | Caregiver/  caregiver | 4.49 | Oppositional Defiant Disorder | 4.49 | 0.00 | High risk | -0.151 | 0.059 |
| Morcillo (2011; F) [1] | USA/Puerto Rico | Propensity score matching | 589 | Females | High Family Bonding | Combined | Caregiver/  mixed | 8.00 | Conduct Problems | 9.00 | 1.00 | High quality | -0.059 | 0.016 |
| Morcillo (2011; F) [2] | USA/Puerto Rico | Propensity score matching | 618 | Females | High Family Bonding | Combined | Caregiver/  mixed | 11.00 | Conduct Problems | 12.00 | 1.00 | High quality | -0.107 | 0.021 |
| Morcillo (2011; M) [1] | USA/Puerto Rico | Propensity score matching | 631 | Males | High Family Bonding | Combined | Caregiver/  mixed | 8.00 | Conduct Problems | 9.00 | 1.00 | High quality | -0.036 | 0.012 |
| Morcillo (2011; M) [2] | USA/Puerto Rico | Propensity score matching | 653 | Males | High Family Bonding | Combined | Caregiver/  mixed | 11.00 | Conduct Problems | 12.00 | 1.00 | High quality | -0.021 | 0.032 |
| Paine (2021) [1] | UK | Adoption study | 96 | Mixed | High Parental Warmth | Combined | Caregiver/  caregiver | 4.08 | Externalising Behaviour | 5.33 | 1.25 | High quality | -0.420 | 0.085 |
| Reuben (2016) [1] | USA | Adoption study | 225 | Mixed | High Parental Warmth | Maternal | Caregiver/  teacher | 2.25 | Externalising Behaviour | 6.50 | 4.25 | High quality | -0.182 | 0.066 |
| Reuben (2016) [3] | USA | Adoption study | 225 | Mixed | High Parental Warmth | Paternal | Caregiver/  teacher | 2.25 | Externalising Behaviour | 6.50 | 4.25 | High quality | 0.086 | 0.070 |
| Shewark (2021) [2] | USA | Adoption study | 561 | Mixed | High Parental Warmth | Maternal | Caregiver/  caregiver | 6.00 | Externalising Behaviour | 7.00 | 1.00 | High quality | -0.130 | 0.042 |
| Shewark (2021) [4] | USA | Adoption study | 561 | Mixed | High Parental Warmth | Paternal | Caregiver/  caregiver | 6.00 | Externalising Behaviour | 7.00 | 1.00 | High quality | -0.120 | 0.080 |
| Vrolik (2021) [1] | The Netherlands | Discordant twin study | 497 | Mixed | High Autonomy Support | Maternal | Self/  self | 18.00 | Externalising Behaviour | 19.00 | 1.00 | High quality | -0.060 | 0.045 |
| Vrolik (2021) [2] | The Netherlands | Discordant twin study | 497 | Mixed | High Autonomy Support | Maternal | Caregiver/  self | 18.00 | Externalising Behaviour | 19.00 | 1.00 | High quality | 0.010 | 0.045 |
| Vrolik (2021) [3] | The Netherlands | Discordant twin study | 497 | Mixed | High Autonomy Support | Paternal | Self/  self | 18.00 | Externalising Behaviour | 19.00 | 1.00 | High quality | 0.070 | 0.045 |
| Vrolik (2021) [4] | The Netherlands | Discordant twin study | 497 | Mixed | High Autonomy Support | Paternal | Caregiver/  self | 18.00 | Externalising Behaviour | 19.00 | 1.00 | High quality | 0.010 | 0.045 |
| Waller, Hyde (2018) [1] | USA | Discordant twin study | 374 | Mixed | High Parental Warmth | Combined | Mixed/  caregiver | 7.80 | Other DBD | 7.80 | 0.00 | Very high risk | 0.458 | 0.073 |
| *Abbreviations: Age^EXP^ = offspring age at exposure assessment; Age^OUT^ = offspring age at outcome assessment; Time = time between exposure and outcome assessment; RoB = risk of bias; ES = effect size; SE = standard error* | | | | | | | | | | | | | | |

### **Table S11.** Summary of all the effect sizes included in the meta-analyses of negative parenting practices.

| **Reference** | **Country** | **Method** | **Sample size** | **Sex** | **Risk factor** | **Maternal/**  **paternal** | **Informant** | ***Age^EXP^*** | **Outcome** | ***Age^OUT^*** | **Time** | **RoB** | **ES** | **SE** |
| --- | --- | --- | --- | --- | --- | --- | --- | --- | --- | --- | --- | --- | --- | --- |
| Asbury (2003) [1] | UK | Discordant twin study | 4,268 | Mixed | Harsh Discipline | Combined | Caregiver/  caregiver | 4.00 | Conduct Problems | 4.00 | 0.000 | Very high risk | 0.150 | 0.058 |
| Asbury (2003) [2] | UK | Discordant twin study | 4,268 | Mixed | Negative Parental Feeling | Combined | Caregiver/  caregiver | 4.00 | Conduct Problems | 4.00 | 0.000 | Very high risk | 0.220 | 0.085 |
| Asbury (2006) [10] | UK | Discordant twin study | 4,090 | Mixed (whole sample) | Negative Parental Feeling | Combined | Caregiver/  teacher | 4.00 | Conduct Problems | 7.00 | 3.000 | High quality | 0.020 | 0.022 |
| Asbury (2006) [9] | UK | Discordant twin study | 4,090 | Mixed (whole sample) | Harsh Discipline | Combined | Caregiver/  teacher | 4.00 | Conduct Problems | 7.00 | 3.000 | High quality | 0.070 | 0.036 |
| Barnett (2013) [2] | USA | Discordant sibling study | 274 | Mixed (whole sample) | Coercive Parenting | Maternal | Observer/  caregiver | 2.98 | Externalising Behaviour | 3.98 | 1.000 | High risk | 0.091 | 0.118 |
| Besemer (2016) [1] | USA | Within-person fixed effects | 499 | Males | Harsh Discipline | Maternal | Caregiver/  mixed | 8.65 | Oppositional Defiant Disorder | 10.90 | 2.250 | High quality | 0.022 | 0.015 |
| Besemer (2016) [2] | USA | Within-person fixed effects | 499 | Males | Low Parental Involvement | Maternal | Caregiver/  mixed | 8.65 | Oppositional Defiant Disorder | 10.90 | 2.250 | High quality | -0.057 | 0.032 |
| Besemer (2016) [3] | USA | Within-person fixed effects | 499 | Males | Poor Parent-Child Communication | Maternal | Caregiver/  mixed | 8.65 | Oppositional Defiant Disorder | 10.90 | 2.250 | High quality | 0.045 | 0.043 |
| Boyle (2004) [1] | Canada | Discordant sibling study | 2,128 | Mixed | Harsh Discipline | Maternal | Caregiver/  caregiver | 10.10 | Externalising Behaviour | 10.10 | 0.000 | High risk | 0.210 | 0.014 |
| Boyle (2004) [2] | Canada | Discordant sibling study | 1,618 | Mixed | Harsh Discipline | Maternal | Caregiver/  teacher | 10.10 | Externalising Behaviour | 10.10 | 0.000 | High risk | 0.110 | 0.014 |
| Boyle (2004) [3] | USA | Discordant sibling study | 7,392 | Mixed | Parental Hostility | Maternal | Caregiver/  caregiver | 7.47 | Externalising Behaviour | 7.47 | 0.000 | High risk | 0.330 | 0.008 |
| Boyle (2004) [5] | USA | Discordant sibling study | 3,757 | Mixed | Parental Hostility | Maternal | Teacher/  teacher | 7.47 | Externalising Behaviour | 7.47 | 0.000 | High risk | 0.170 | 0.015 |
| Boyle (2004) [7] | USA | Discordant sibling study | 2,876 | Mixed | Harsh Discipline | Maternal | Caregiver/  caregiver | 9.03 | Externalising Behaviour | 9.03 | 0.000 | High risk | 0.130 | 0.039 |
| Burt (2006) [1] | USA | Discordant twin study | 466 | Males | High Parent-Child Conflict | Maternal | Mixed/  mixed | 11.00 | Externalising Behaviour | 14.00 | 3.000 | High risk | 0.050 | 0.046 |
| Burt (2006) [2] | USA | Discordant twin study | 415 | Males | High Parent-Child Conflict | Maternal | Mixed/  mixed | 14.00 | Externalising Behaviour | 17.00 | 3.000 | High risk | 0.130 | 0.048 |
| Burt (2006) [3] | USA | Discordant twin study | 435 | Females | High Parent-Child Conflict | Maternal | Mixed/  mixed | 11.00 | Externalising Behaviour | 14.00 | 3.000 | High risk | 0.130 | 0.047 |
| Burt (2006) [4] | USA | Discordant twin study | 409 | Females | High Parent-Child Conflict | Maternal | Mixed/  mixed | 14.00 | Externalising Behaviour | 17.00 | 3.000 | High risk | 0.080 | 0.049 |
| Burt (2006) [5] | USA | Discordant twin study | 901 | Mixed | High Parent-Child Conflict | Maternal | Mixed/  mixed | 11.00 | Externalising Behaviour | 14.00 | 3.000 | High risk | 0.090 | 0.033 |
| Burt (2006) [6] | USA | Discordant twin study | 824 | Mixed | High Parent-Child Conflict | Maternal | Mixed/  mixed | 14.00 | Externalising Behaviour | 17.00 | 3.000 | High risk | 0.110 | 0.034 |
| Burt (2021) [1] | USA | Discordant twin study | 426 | Mixed | High Parent-Child Conflict | Combined | Mixed/  mixed | 8.08 | Externalising Behaviour | 8.08 | 0.000 | Very high risk | 0.050 | 0.015 |
| Burt (2021) [2] | USA | Discordant twin study | 480 | Mixed | High Parent-Child Conflict | Maternal | Caregiver/  caregiver | 10.55 | Externalising Behaviour | 10.55 | 0.000 | Very high risk | 0.070 | 0.023 |
| Caspi (2004) [1] | UK | Discordant twin study | 1,212 | Mixed | Parental Criticism | Maternal | Caregiver/  caregiver | 5.00 | Externalising Behaviour | 6.50 | 1.500 | High quality | 0.160 | 0.062 |
| Caspi (2004) [2] | UK | Discordant twin study | 1,212 | Mixed | Parental Criticism | Maternal | Caregiver/  caregiver | 5.00 | Externalising Behaviour | 6.50 | 1.500 | High quality | 0.160 | 0.062 |
| Caspi (2004) [5] | UK | Discordant twin study | 1,126 | Mixed | Parental Criticism | Maternal | Caregiver/  teacher | 5.00 | Externalising Behaviour | 6.50 | 1.500 | High quality | 0.150 | 0.058 |
| Caspi (2004) [6] | UK | Discordant twin study | 1,126 | Mixed | Parental Criticism | Maternal | Caregiver/  teacher | 5.00 | Externalising Behaviour | 6.50 | 1.500 | High quality | 0.100 | 0.051 |
| Cecil (2012) [1] | UK | Discordant twin study | 5,184 | Mixed | Harsh Discipline | Combined | Caregiver/  teacher | 5.75 | Conduct Problems | 12.00 | 6.250 | High quality | 0.140 | 0.056 |
| Cecil (2012) [2] | UK | Discordant twin study | 5,184 | Mixed | Negative Parental Feeling | Combined | Caregiver/  teacher | 5.75 | Conduct Problems | 12.00 | 6.250 | High quality | 0.040 | 0.023 |
| Cree (2021) [1] | USA | Adoption study | 337 | Mixed | Overreactive Parenting | Maternal | Caregiver (M)/  caregiver (F) | 1.50 | Externalising Behaviour | 4.50 | 3.000 | High quality | 0.224 | 0.103 |
| Ganiban (2021) [1] | USA | Adoption study | 361 | Mixed | Low Parental Involvement | Combined | Caregiver (M/F)/  caregiver (F/M) | 2.25 | Externalising Behaviour | 7.00 | 4.750 | High quality | 0.024 | 0.053 |
| Ganiban (2021) [2] | USA | Adoption study | 361 | Mixed | Overreactive Parenting | Combined | Caregiver (M/F)/  caregiver (F/M) | 2.25 | Externalising Behaviour | 7.00 | 4.750 | High quality | 0.051 | 0.053 |
| Glover (2010) [2] | USA | Adoption study | 85 | Mixed | Negative Parental Feeling | Maternal | Caregiver (M)/  caregiver (F) | 5.59 | Conduct Problems | 5.59 | 0.000 | Very high risk | 0.320 | 0.124 |
| Glover (2010) [4] | USA | Adoption study | 85 | Mixed | Negative Parental Feeling | Paternal | Caregiver (F)/  caregiver (M) | 5.59 | Conduct Problems | 5.59 | 0.000 | Very high risk | 0.470 | 0.142 |
| Harold (2012) [1] | UK/USA | IVF study | 207 | Mixed | Parental Hostility | Maternal | Caregiver/  caregiver | 6.72 | Conduct Problems | 6.72 | 0.000 | Very high risk | 0.240 | 0.093 |
| Harold (2012) [2] | UK/USA | IVF study | 170 | Mixed | Parental Hostility | Paternal | Caregiver/  caregiver | 6.72 | Conduct Problems | 6.72 | 0.000 | Very high risk | 0.350 | 0.136 |
| Harold (2013) [1] | USA | Adoption study | 218 | Mixed | Parental Hostility | Maternal | Caregiver/  caregiver | 5.98 | Conduct Problems | 5.98 | 0.000 | High quality | 0.310 | 0.094 |
| Harold (2013) [2] | USA | Adoption study | 218 | Mixed | Parental Hostility | Maternal | Caregiver/  caregiver | 5.98 | Conduct Problems | 5.98 | 0.000 | High quality | 0.210 | 0.081 |
| Harold (2013) [3] | USA | Adoption study | 218 | Mixed | Parental Hostility | Paternal | Caregiver/  caregiver | 5.98 | Conduct Problems | 5.98 | 0.000 | High quality | 0.440 | 0.133 |
| Harold (2013) [4] | USA | Adoption study | 218 | Mixed | Parental Hostility | Paternal | Caregiver (F)/  caregiver (M) | 5.98 | Conduct Problems | 5.98 | 0.000 | High quality | 0.340 | 0.103 |
| Hou (2013) [2] | China | Discordant twin study | 690 | Mixed | Parental Hostility | Maternal | Mixed/  mixed | 13.86 | Conduct Problems | 15.36 | 1.500 | High quality | 0.000 | 0.054 |
| Hou (2013) [4] | China | Discordant twin study | 690 | Mixed | Parental Hostility | Paternal | Mixed/  mixed | 13.86 | Conduct Problems | 15.36 | 1.500 | High quality | 0.020 | 0.054 |
| Klahr, McGue (2011) | USA | Adoption study | 672 | Mixed | High Parent-Child Conflict | Combined | Caregiver/  self | 14.10 | Conduct Problems | 18.20 | 4.100 | High quality | 0.120 | 0.036 |
| Klahr, Rueter (2011) [1] | USA | Adoption study | 390 | Mixed | High Parent-Child Conflict | Combined | Caregiver/  self | 14.00 | Other DBD | 14.00 | 0.000 | High risk | 0.400 | 0.155 |
| Klahr, Rueter (2011) [2] | USA | Adoption study | 390 | Mixed | Coercive Parenting | Combined | Observer/  self | 14.00 | Other DBD | 14.00 | 0.000 | High risk | 0.090 | 0.050 |
| Klahr, Rueter (2011) [3] | USA | Adoption study | 396 | Mixed | High Parent-Child Conflict | Combined | Caregiver/  observer | 14.00 | Other DBD | 14.00 | 0.000 | High risk | 0.260 | 0.101 |
| Klahr, Rueter (2011) [4] | USA | Adoption study | 396 | Mixed | Coercive Parenting | Combined | Observer/  observer | 14.00 | Other DBD | 14.00 | 0.000 | High risk | 0.330 | 0.128 |
| Kullberg (2024) [1] | UK | Discordant twin study | 5,698 | Mixed | Harsh Discipline | Combined | Self/  self | 9.00 | Conduct Problems | 12.00 | 3.000 | High quality | -0.020 | 0.040 |
| Kullberg (2024) [2] | UK | Discordant twin study | 5,698 | Mixed | Harsh Discipline | Combined | Self/  self | 12.00 | Conduct Problems | 16.00 | 4.000 | High quality | 0.040 | 0.040 |
| Latham (2017) [1] | UK | Discordant sibling study | 212 | Mixed | Coercive Parenting | Maternal | Caregiver/  caregiver | 3.92 | Conduct Problems | 5.92 | 2.000 | High quality | 0.020 | 0.160 |
| Latham (2017) [2] | UK | Discordant sibling study | 212 | Mixed | Coercive Parenting | Paternal | Caregiver/  caregiver | 3.90 | Conduct Problems | 5.92 | 2.020 | High quality | -0.220 | 0.150 |
| Lipscomb (2014) | USA | Adoption study | 233 | Mixed | Overreactive Parenting | Combined | Caregiver/  caregiver | 3.00 | Externalising Behaviour | 6.00 | 3.000 | High quality | 0.130 | 0.039 |
| Long (2015) [10] | USA | Discordant twin study | 2,606 | Mixed | Low Parental Warmth | Maternal | Self/  self | 36.69 | Antisocial Personality Disorder | 36.69 | 0.000 | Very high risk | 0.034 | 0.030 |
| Long (2015) [11] | USA | Discordant twin study | 2,606 | Mixed | Overreactive Parenting | Paternal | Self/  self | 36.69 | Antisocial Personality Disorder | 36.69 | 0.000 | Very high risk | -0.071 | 0.030 |
| Long (2015) [12] | USA | Discordant twin study | 2,606 | Mixed | Harsh Discipline | Paternal | Self/  self | 36.69 | Antisocial Personality Disorder | 36.69 | 0.000 | Very high risk | 0.015 | 0.040 |
| Long (2015) [1] | USA | Discordant twin study | 2,606 | Mixed | Low Parental Warmth | Maternal | Self/  self | 36.69 | Conduct Disorder | 36.69 | 0.000 | Very high risk | 0.037 | 0.030 |
| Long (2015) [2] | USA | Discordant twin study | 2,606 | Mixed | Overreactive Parenting | Maternal | Self/  self | 36.69 | Conduct Disorder | 36.69 | 0.000 | Very high risk | -0.024 | 0.030 |
| Long (2015) [3] | USA | Discordant twin study | 2,606 | Mixed | Harsh Discipline | Maternal | Self/  self | 36.69 | Conduct Disorder | 36.69 | 0.000 | Very high risk | 0.047 | 0.040 |
| Long (2015) [4] | USA | Discordant twin study | 2,606 | Mixed | Low Parental Warmth | Paternal | Self/  self | 36.69 | Conduct Disorder | 36.69 | 0.000 | Very high risk | 0.025 | 0.030 |
| Long (2015) [5] | USA | Discordant twin study | 2,606 | Mixed | Overprotective Parenting | Paternal | Self/  self | 36.69 | Conduct Disorder | 36.69 | 0.000 | Very high risk | -0.005 | 0.030 |
| Long (2015) [6] | USA | Discordant twin study | 2,606 | Mixed | Harsh Discipline | Paternal | Self/  self | 36.69 | Conduct Disorder | 36.69 | 0.000 | Very high risk | 0.084 | 0.040 |
| Long (2015) [7] | USA | Discordant twin study | 2,606 | Mixed | Low Parental Warmth | Maternal | Self/  self | 36.69 | Antisocial Personality Disorder | 36.69 | 0.000 | Very high risk | -0.006 | 0.030 |
| Long (2015) [8] | USA | Discordant twin study | 2,606 | Mixed | Overprotective Parenting | Maternal | Self/  self | 36.69 | Antisocial Personality Disorder | 36.69 | 0.000 | Very high risk | -0.075 | 0.030 |
| Long (2015) [9] | USA | Discordant twin study | 2,606 | Mixed | Harsh Discipline | Maternal | Self/  self | 36.69 | Antisocial Personality Disorder | 36.69 | 0.000 | Very high risk | 0.070 | 0.040 |
| Lysenko (2013) [1] | UK | Discordant sibling study | 9,096 | Males | Harsh Discipline | Maternal | Caregiver/  caregiver | 4.00 | Conduct Problems | 7.00 | 3.000 | High risk | 0.321 | 0.048 |
| Lysenko (2013) [2] | UK | Discordant sibling study | 9,096 | Females | Harsh Discipline | Maternal | Caregiver/  caregiver | 4.00 | Conduct Problems | 7.00 | 3.000 | High risk | 0.357 | 0.054 |
| Marceau (2013) | USA | Adoption study | 561 | Mixed | Overreactive Parenting | Combined | Caregiver/  caregiver | 1.50 | Externalising Behaviour | 2.25 | 0.750 | High quality | 0.150 | 0.076 |
| Mark (2017) [2] | UK | Discordant sibling study | 156 | Mixed | High Parent-Child Conflict | Maternal | Self/  caregiver | 10.94 | Externalising Behaviour | 10.94 | 0.000 | Very high risk | 0.299 | 0.112 |
| Narusyte (2011) [1] | Sweden | Extended children of twins study | 3,540 | Mixed | Parental Criticism | Paternal | Caregiver/  self | 16.20 | Externalising Behaviour | 16.20 | 0.000 | High quality | 0.240 | 0.038 |
| Narusyte (2011) [2] | Sweden | Extended children of twins study | 3,540 | Mixed | Parental Criticism | Maternal | Caregiver/  self | 16.20 | Externalising Behaviour | 16.20 | 0.000 | High quality | -0.090 | 0.046 |
| Oliver (2015) | UK | Discordant twin study | 6,308 | Mixed | Negative Parental Feeling | Combined | Caregiver/  caregiver | 5.56 | Conduct Problems | 9.02 | 3.465 | High quality | 0.100 | 0.039 |
| Pike (1996) [10] | USA | Discordant twin study | 186 | Mixed | High Parent-Child Conflict | Maternal | Observer/  caregiver | 13.71 | Other DBD | 13.71 | 0.000 | Very high risk | -0.070 | 0.104 |
| Pike (1996) [11] | USA | Discordant twin study | 186 | Mixed | High Parent-Child Conflict | Maternal | Observer/  caregiver | 13.71 | Other DBD | 13.71 | 0.000 | Very high risk | 0.100 | 0.104 |
| Pike (1996) [12] | USA | Discordant twin study | 186 | Mixed | High Parent-Child Conflict | Maternal | Observer/  observer | 13.71 | Other DBD | 13.71 | 0.000 | Very high risk | 0.290 | 0.148 |
| Pike (1996) [13] | USA | Discordant twin study | 186 | Mixed | High Parent-Child Conflict | Paternal | Self/  self | 13.71 | Other DBD | 13.71 | 0.000 | Very high risk | 0.390 | 0.198 |
| Pike (1996) [14] | USA | Discordant twin study | 186 | Mixed | High Parent-Child Conflict | Paternal | Self/  caregiver | 13.71 | Other DBD | 13.71 | 0.000 | Very high risk | -0.040 | 0.105 |
| Pike (1996) [16] | USA | Discordant twin study | 186 | Mixed | High Parent-Child Conflict | Paternal | Self/  caregiver | 13.71 | Other DBD | 13.71 | 0.000 | Very high risk | 0.100 | 0.104 |
| Pike (1996) [17] | USA | Discordant twin study | 186 | Mixed | High Parent-Child Conflict | Paternal | Self/  observer | 13.71 | Other DBD | 13.71 | 0.000 | Very high risk | -0.150 | 0.102 |
| Pike (1996) [18] | USA | Discordant twin study | 186 | Mixed | High Parent-Child Conflict | Paternal | Caregiver/  self | 13.71 | Other DBD | 13.71 | 0.000 | Very high risk | 0.140 | 0.103 |
| Pike (1996) [19] | USA | Discordant twin study | 186 | Mixed | High Parent-Child Conflict | Paternal | Caregiver (F)/  caregiver (M) | 13.71 | Other DBD | 13.71 | 0.000 | Very high risk | 0.190 | 0.101 |
| Pike (1996) [1] | USA | Discordant twin study | 186 | Mixed | High Parent-Child Conflict | Maternal | Self/  self | 13.71 | Other DBD | 13.71 | 0.000 | Very high risk | 0.330 | 0.168 |
| Pike (1996) [20] | USA | Discordant twin study | 186 | Mixed | High Parent-Child Conflict | Paternal | Caregiver/  caregiver | 13.71 | Other DBD | 13.71 | 0.000 | Very high risk | 0.540 | 0.275 |
| Pike (1996) [21] | USA | Discordant twin study | 186 | Mixed | High Parent-Child Conflict | Paternal | Caregiver/  observer | 13.71 | Other DBD | 13.71 | 0.000 | Very high risk | 0.220 | 0.112 |
| Pike (1996) [22] | USA | Discordant twin study | 186 | Mixed | High Parent-Child Conflict | Paternal | Observer/  self | 13.71 | Other DBD | 13.71 | 0.000 | Very high risk | -0.070 | 0.104 |
| Pike (1996) [23] | USA | Discordant twin study | 186 | Mixed | High Parent-Child Conflict | Paternal | Observer/  caregiver | 13.71 | Other DBD | 13.71 | 0.000 | Very high risk | 0.020 | 0.105 |
| Pike (1996) [24] | USA | Discordant twin study | 186 | Mixed | High Parent-Child Conflict | Paternal | Observer/  caregiver | 13.71 | Other DBD | 13.71 | 0.000 | Very high risk | 0.060 | 0.104 |
| Pike (1996) [25] | USA | Discordant twin study | 186 | Mixed | High Parent-Child Conflict | Paternal | Observer/  observer | 13.71 | Other DBD | 13.71 | 0.000 | Very high risk | 0.290 | 0.148 |
| Pike (1996) [2] | USA | Discordant twin study | 186 | Mixed | High Parent-Child Conflict | Maternal | Self/  caregiver | 13.71 | Other DBD | 13.71 | 0.000 | Very high risk | -0.130 | 0.103 |
| Pike (1996) [3] | USA | Discordant twin study | 186 | Mixed | High Parent-Child Conflict | Maternal | Self/  caregiver | 13.71 | Other DBD | 13.71 | 0.000 | Very high risk | 0.060 | 0.104 |
| Pike (1996) [4] | USA | Discordant twin study | 186 | Mixed | High Parent-Child Conflict | Maternal | Self/  observer | 13.71 | Other DBD | 13.71 | 0.000 | Very high risk | -0.140 | 0.103 |
| Pike (1996) [5] | USA | Discordant twin study | 186 | Mixed | High Parent-Child Conflict | Maternal | Caregiver/  self | 13.71 | Other DBD | 13.71 | 0.000 | Very high risk | 0.200 | 0.101 |
| Pike (1996) [6] | USA | Discordant twin study | 186 | Mixed | High Parent-Child Conflict | Maternal | Caregiver/  caregiver | 13.71 | Other DBD | 13.71 | 0.000 | Very high risk | 0.270 | 0.137 |
| Pike (1996) [7] | USA | Discordant twin study | 186 | Mixed | High Parent-Child Conflict | Maternal | Caregiver (M)/  caregiver (F) | 13.71 | Other DBD | 13.71 | 0.000 | Very high risk | 0.470 | 0.239 |
| Pike (1996) [8] | USA | Discordant twin study | 186 | Mixed | High Parent-Child Conflict | Maternal | Caregiver/  observer | 13.71 | Other DBD | 13.71 | 0.000 | Very high risk | 0.290 | 0.148 |
| Pike (1996) [9] | USA | Discordant twin study | 186 | Mixed | High Parent-Child Conflict | Maternal | Observer/  self | 13.71 | Other DBD | 13.71 | 0.000 | Very high risk | -0.110 | 0.104 |
| Reuben (2016) [2] | USA | Adoption study | 225 | Mixed | Overreactive Parenting | Maternal | Caregiver/  teacher | 2.25 | Externalising Behaviour | 6.50 | 4.250 | High quality | 0.019 | 0.068 |
| Reuben (2016) [4] | USA | Adoption study | 225 | Mixed | Overreactive Parenting | Paternal | Caregiver/  teacher | 2.25 | Externalising Behaviour | 6.50 | 4.250 | High quality | -0.010 | 0.072 |
| Richmond (2006) [1] | Not reported | Discordant sibling study | 186 | Mixed | High Parent-Child Conflict | Maternal | Caregiver/  caregiver | 15.00 | Externalising Behaviour | 15.00 | 0.000 | Very high risk | 0.021 | 0.005 |
| Richmond (2006) [2] | Not reported | Discordant sibling study | 186 | Mixed | High Parent-Child Conflict | Paternal | Caregiver/  caregiver | 15.00 | Externalising Behaviour | 15.00 | 0.000 | Very high risk | 0.016 | 0.005 |
| Richmond (2009) [1] | Not reported | Discordant sibling study | 228 | Mixed | High Parent-Child Conflict | Maternal | Caregiver/  self | 15.00 | Externalising Behaviour | 15.00 | 0.000 | Very high risk | 0.299 | 0.100 |
| Richmond (2009) [2] | Not reported | Discordant sibling study | 228 | Mixed | High Parent-Child Conflict | Paternal | Caregiver/  self | 15.00 | Externalising Behaviour | 15.00 | 0.000 | Very high risk | 0.355 | 0.118 |
| Riggins-Caspers (2003) [2] | Not reported | Adoption study | 150 | Mixed | Harsh Discipline | Combined | Caregiver/  self |  | Conduct Disorder |  |  | Very high risk | 0.250 | 0.097 |
| Riggins-Caspers (2003) [4] | Not reported | Adoption study | 150 | Mixed | Harsh Discipline | Combined | Caregiver/  self |  | Oppositional Defiant Disorder |  |  | Very high risk | 0.420 | 0.127 |
| Rolon-Arroyo (2018) [1] | USA | Within-person fixed effects | 162 | Mixed | Overreactive Parenting | Maternal | Caregiver/  caregiver | 5.76 | Conduct Disorder | 6.74 | 0.980 | High quality | 0.110 | 0.050 |
| Rolon-Arroyo (2018) [2] | USA | Within-person fixed effects | 162 | Mixed | Overreactive Parenting | Maternal | Caregiver/  caregiver | 5.76 | Oppositional Defiant Disorder | 6.74 | 0.980 | High quality | 0.180 | 0.050 |
| Roos (2016) | USA | Adoption study | 293 | Mixed | Low Parental Involvement | Maternal | Caregiver/  caregiver | 4.50 | Externalising Behaviour | 6.50 | 2.000 | High quality | 0.139 | 0.066 |
| Samek (2014) [1] | USA | Adoption study | 533 | Mixed | Low Parental Involvement | Combined | Self/  self | 18.24 | Antisocial Personality Disorder | 23.00 | 4.760 | High quality | -0.300 | 0.153 |
| Samek (2014) [2] | USA | Adoption study | 533 | Mixed | High Parent-Child Conflict | Combined | Self/  self | 18.24 | Antisocial Personality Disorder | 23.00 | 4.760 | High quality | 0.370 | 0.188 |
| Samek (2014) [3] | USA | Adoption study | 533 | Mixed | Negative Parent-Child Relationship | Combined | Self/  self | 18.24 | Antisocial Personality Disorder | 23.00 | 4.760 | High quality | 0.270 | 0.137 |
| Shelton (2008) [1] | UK | Discordant twin study | 462 | Mixed | High Parental Hostility | Maternal | Self/  self | 15.28 | Conduct Problems | 22.28 | 7.000 | High quality | 0.279 | 0.071 |
| Shelton (2008) [2] | UK | Discordant twin study | 462 | Mixed | High Parental Hostility | Maternal | Caregiver/  caregiver | 15.28 | Conduct Problems | 22.28 | 7.000 | High quality | 0.368 | 0.094 |
| Shelton (2008) [3] | UK | Discordant twin study | 462 | Mixed | Low Parental Warmth | Maternal | Self/  self | 15.28 | Conduct Problems | 22.28 | 7.000 | High quality | 0.031 | 0.066 |
| Shelton (2008) [4] | UK | Discordant twin study | 462 | Mixed | Low Parental Warmth | Maternal | Caregiver/  caregiver | 15.28 | Conduct Problems | 22.28 | 7.000 | High quality | 0.179 | 0.069 |
| Shewark (2021) [1] | USA | Adoption study | 561 | Mixed | Parental Hostility | Maternal | Caregiver/  caregiver | 6.00 | Externalising Behaviour | 7.00 | 1.000 | High quality | 0.420 | 0.035 |
| Shewark (2021) [3] | USA | Adoption study | 561 | Mixed | Parental Hostility | Paternal | Caregiver/  caregiver | 6.00 | Externalising Behaviour | 7.00 | 1.000 | High quality | 0.230 | 0.040 |
| Viding (2009) [1] | UK | Discordant twin study | 4,056 | Mixed | Harsh Discipline | Maternal | Caregiver/  caregiver | 7.00 | Conduct Problems | 7.00 | 0.000 | High quality | 0.460 | 0.139 |
| Viding (2009) [2] | UK | Discordant twin study | 4,056 | Mixed | Harsh Discipline | Maternal | Caregiver/  teacher | 7.00 | Conduct Problems | 7.00 | 0.000 | High quality | 0.120 | 0.036 |
| Viding (2009) [3] | UK | Discordant twin study | 4,056 | Mixed | Harsh Discipline | Maternal | Caregiver/  caregiver | 7.00 | Conduct Problems | 12.00 | 5.000 | High quality | 0.200 | 0.060 |
| Viding (2009) [4] | UK | Discordant twin study | 4,056 | Mixed | Harsh Discipline | Maternal | Caregiver/  teacher | 7.00 | Conduct Problems | 12.00 | 5.000 | High quality | 0.070 | 0.027 |
| Viding (2009) [5] | UK | Discordant twin study | 4,056 | Mixed | Harsh Discipline | Maternal | Caregiver/  caregiver | 7.00 | Conduct Problems | 12.00 | 5.000 | High quality | 0.120 | 0.036 |
| Viding (2009) [6] | UK | Discordant twin study | 4,056 | Mixed | Harsh Discipline | Maternal | Caregiver/  teacher | 7.00 | Conduct Problems | 12.00 | 5.000 | High quality | 0.070 | 0.027 |
| Waller, Hyde (2018) [2] | USA | Discordant twin study | 374 | Mixed | Parental Hostility | Combined | Mixed/  caregiver | 7.80 | Other DBD | 7.80 | 0.000 | Very high risk | 0.137 | 0.172 |
| *Abbreviations: Age^EXP^ = offspring age at exposure assessment; Age^OUT^ = offspring age at outcome assessment; Time = time between exposure and outcome assessment; RoB = risk of bias; ES = effect size; SE = standard error* | | | | | | | | | | | | | | |

### **Table S12.** Descriptive summary of the participant characteristics and study features of the studies included in the meta-analysis by risk of bias category.

|  | **Very high risk** | | **High risk** | | **High quality** | |
| --- | --- | --- | --- | --- | --- | --- |
|  | k | % | k | % | k | % |
| **Average percentage female** | - | 49.1 | - | 51.1 | - | 47.1 |
| **Average percentage of mothers** | - | 68.1 | - | 92.9 | - | 78.7 |
| **Average percentage of fathers** | - | 31.9 | - | 7.1 | - | 19.6 |
| **Majority ancestry** † |  |  |  |  |  |  |
| Caucasian | 8 | 66.7 | 2 | 33.3 | 12 | 44.4 |
| African American | - | - | 1 | 16.7 | - | - |
| Asian | - | - | 1 | 16.7 | 3 | 11.1 |
| Hispanic | - | - | - | - | 1 | 3.7 |
| Not reported | 4 | 33.3 | 2 | 33.3 | 11 | 40.7 |
| **Year of publication** |  |  |  |  |  |  |
| 1995 - 1999 | 1 | 8.3 | - | - | - | - |
| 2000 - 2004 | 2 | 16.7 | - | - | - | - |
| 2005 - 2009 | 2 | 16.7 | 2 | 33.3 | 4 | 14.8 |
| 2010 - 2014 | 3 | 25.0 | 4 | 66.7 | 8 | 29.6 |
| 2015 - 2019 | 3 | 25.0 | - | - | 8 | 29.6 |
| 2020 - 2024 | 1 | 8.3 | - | - | 7 | 25.9 |
| **Geographical region** †† |  |  |  |  |  |  |
| USA | 24 | 51.1 | 24 | 51.1 | 24 | 51.1 |
| UK | 14 | 29.8 | 14 | 29.8 | 14 | 29.8 |
| Not reported | 3 | 6.4 | 3 | 6.4 | 3 | 6.4 |
| Canada | 2 | 4.3 | 2 | 4.3 | 2 | 4.3 |
| China | 1 | 2.1 | 1 | 2.1 | 1 | 2.1 |
| Sweden | 1 | 2.1 | 1 | 2.1 | 1 | 2.1 |
| Puerto Rico | 1 | 2.1 | 1 | 2.1 | 1 | 2.1 |
| The Netherlands | 1 | 2.1 | 1 | 2.1 | 1 | 2.1 |
| **Cohort** †† |  |  |  |  |  |  |
| Early Growth and Development Study | - | - | - | - | 8 | 29.6 |
| Twins Early Development Study | 1 | 7.7 | 1 | 12.5 | 5 | 18.5 |
| Not reported | 3 | 23.1 | - | - | 1 | 3.7 |
| Sibling Interaction and Behavior Study | - | - | 1 | 12.5 | 2 | 7.4 |
| Northeast-Northwest Collaborative Adoption Projects | 2 | 15.4 | - | - | - | - |
| Twin Study of Behavioral and Emotional Development in Children | 2 | 15.4 | - | - | - | - |
| Wales Adoption Cohort Study | - | - | - | - | 2 | 7.4 |
| Beijing Twin Study | - | - | - | - | 1 | 3.7 |
| Boricua Youth Study | - | - | - | - | 1 | 3.7 |
| Cardiff IVF study | 1 | 7.7 | - | - | - | - |
| Cardiff Study of All Wales and North West of England Twins | - | - | - | - | 1 | 3.7 |
| Environmental Risk Longitudinal Twin Study | - | - | - | - | 1 | 3.7 |
| Fragile Families and Child Wellbeing Study | - | - | 1 | 12.5 | - | - |
| Healthy Babies Healthy Children | - | - | 1 | 12.5 | - | - |
| Minnesota Twin Family Study | - | - | 1 | 12.5 | - | - |
| National Longitudinal Study of Youth | - | - | 1 | 12.5 | - | - |
| National Longitudinal Survey of Children and Youth | - | - | 1 | 12.5 | - | - |
| Nonshared Environment and Adolescent Development project | 1 | 7.7 | - | - | - | - |
| Ontario Child Health Study | - | - | 1 | 12.5 | - | - |
| Panel Study of Income Dynamics - Child Development Supplement study | - | - | - | - | 1 | 3.7 |
| Pittsburgh Youth Study | - | - | - | - | 1 | 3.7 |
| Research on Adolescent Development And Relationships | - | - | - | - | 1 | 3.7 |
| Sisters and Brothers Study | 1 | 7.7 | - | - | - | - |
| The Twins, Family and Behaviour | - | - | - | - | 1 | 3.7 |
| Twin Study of Behavioral and Emotional Development in Adolescents | 1 | 7.7 | - | - | - | - |
| Twin and Offspring Study in Sweden and from the Twin Study of Child and Adolescent Development both from the Swedish Twin Registry | - | - | - | - | 1 | 3.7 |
| Virginia Adult Twin Study of Psychiatric and Substance Use Disorders | 1 | 7.7 | - | - | - | - |
| **Quasi-experimental method ††** |  |  |  |  |  |  |
| Adoption study | 3 | 25.0 | 1 | 16.7 | 12 | 44.4 |
| Discordant twin study | 5 | 41.7 | 1 | 16.7 | 9 | 33.3 |
| Discordant sibling study | 3 | 25.0 | 4 | 66.7 | 2 | 7.4 |
| Within-person fixed effects | - | - | - | - | 2 | 7.4 |
| Extended children of twins study | - | - | - | - | 1 | 3.7 |
| IVF study | 1 | 8.3 | - | - | - | - |
| Propensity score matching | - | - | - | - | 1 | 3.7 |
| **Study design** †† |  |  |  |  |  |  |
| Cross-sectional | 7 | 58.3 | 3 | 50.0 | 2 | 7.1 |
| Longitudinal | 5 | 41.7 | 3 | 50.0 | 26 | 92.9 |
| **Informants for the exposure and outcome** †† |  |  |  |  |  |  |
| Discordant | 8 | 61.5 | 4 | 50.0 | 14 | 45.2 |
| Concordant | 5 | 38.5 | 4 | 50.0 | 17 | 54.8 |
| **Number of covariates in analyses** |  |  |  |  |  |  |
| 0 | 5 | 41.7 | - | - | 4 | 14.8 |
| 1 | 3 | 25.0 | 1 | 16.7 | 2 | 7.4 |
| 2 | 2 | 16.7 | 2 | 33.3 | 10 | 37.0 |
| 3 | 1 | 8.3 | - | - | 4 | 14.8 |
| 4 | 1 | 8.3 | - | - | 3 | 11.1 |
| 5 | - | - | 1 | 16.7 | 2 | 7.4 |
| 6 | - | - | 2 | 33.3 | 1 | 3.7 |
| 7 | - | - | - | - | 1 | 3.7 |
| **Type of covariates** †† |  |  |  |  |  |  |
| Child sex | 4 | 28.6 | 6 | 27.3 | 11 | 15.9 |
| Child age | 4 | 28.6 | 4 | 18.2 | 10 | 14.5 |
| Prior DBD | - | - | 2 | 9.1 | 6 | 8.7 |
| Adoption factors | - | - | - | - | 8 | 11.6 |
| Marital status/quality | 2 | 14.3 | 3 | 13.6 | 2 | 2.9 |
| Other factors | 1 | 7.1 | 2 | 9.1 | 4 | 5.8 |
| Socio-economic factors | - | - | 2 | 9.1 | 4 | 5.8 |
| Prior parenting | - | - | 1 | 4.5 | 5 | 7.2 |
| Obstetric complications | - | - | - | - | 6 | 8.7 |
| Ethnicity | 1 | 7.1 | - | - | 3 | 4.3 |
| Other parenting factors | - | - | - | - | 3 | 4.3 |
| Parental psychopathology | 1 | 7.1 | 1 | 4.5 | 1 | 1.4 |
| In utero exposure to toxins | - | - | - | - | 3 | 4.3 |
| Home environment | - | - | - | - | 2 | 2.9 |
| Interactions between variables | - | - | 1 | 4.5 | - | - |
| Birth order | 1 | 7.1 | - | - | - | - |
| Parent age | - | - | - | - | 1 | 1.4 |

*Note:* *very high-risk studies scored below 5.5, high-risk scored between 5.5 and 7 and high-quality studies scored above 7 on the adapted Newcastle Ottawa scale. Abbreviations: k = number of studies; % = percentage;*

# Figures

### **Figure S1.** A simplified representation of the data structure for the meta-analysis


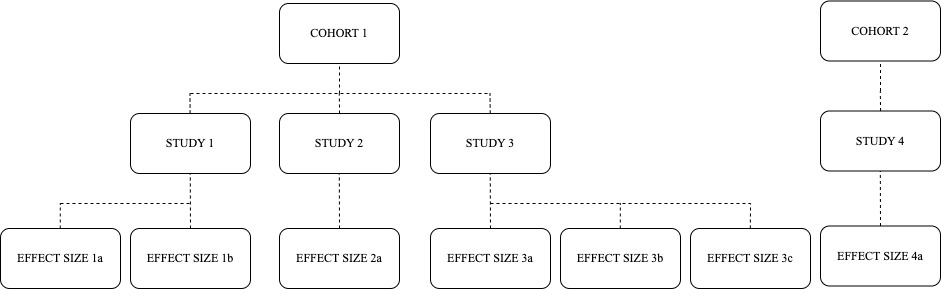


Note. Here effect sizes are nested within studies nested within cohorts, consistent with the three-level random-effects model.

### **Figure S2.** Population attributable impact of negative parenting.


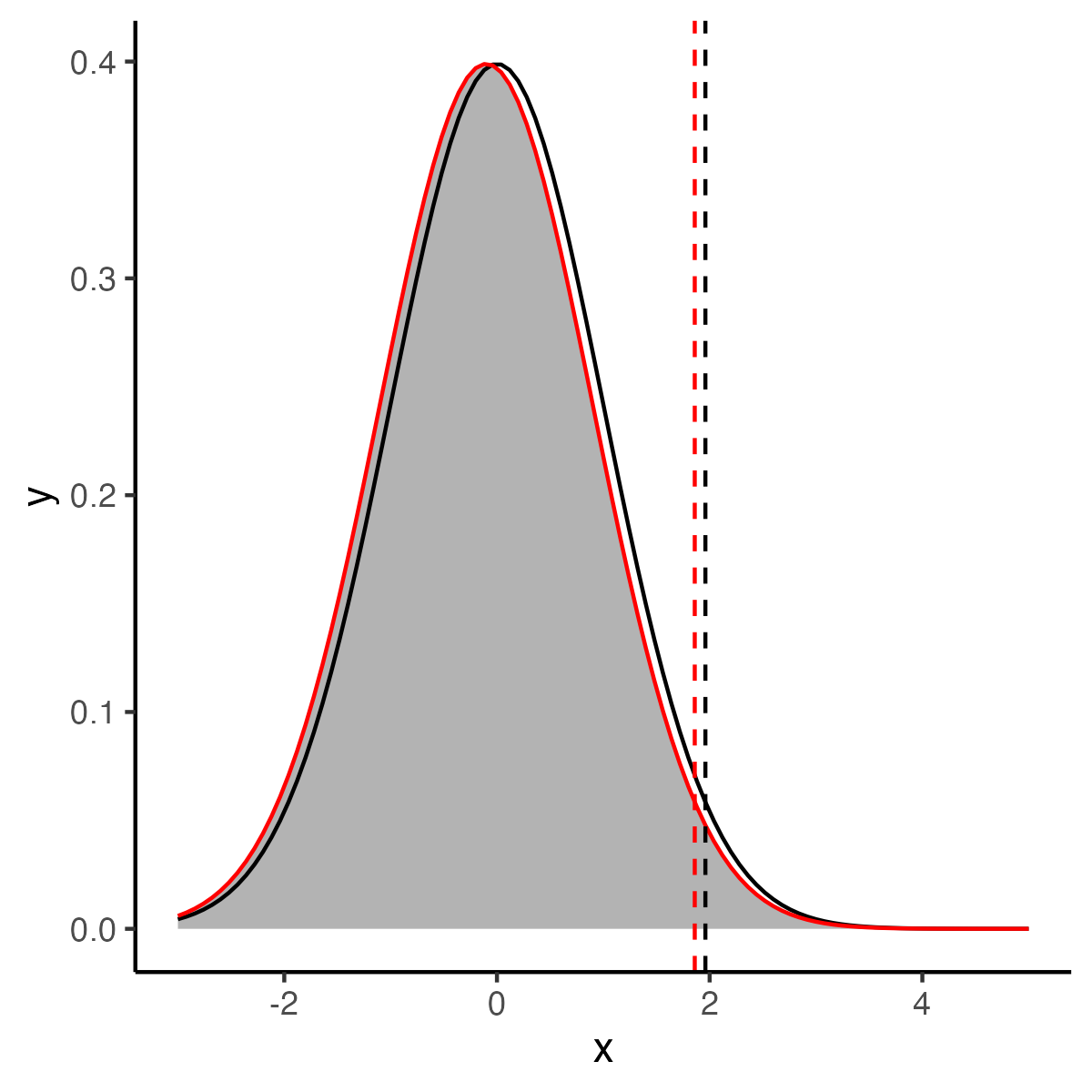


Note. the black vertical line represents ‘z’ the DBD score value above which a diagnosis would be recorded, the red vertical line represents the new value of ‘z’ after a change in parenting practices.

### **Figure S3.** PRISMA flow diagram of search results

Records identified through database searching:

(n = 5970)

Records removed *before screening*:

Duplicate records removed (n = 1444)

Records screened on title and abstract

(n = 4526)

Records excluded

(n = 4060)

Reports assessed for eligibility via full-text

(n = 466)

Reports excluded: (n = 421

- Outcome (n = 34)
- Exposure (n = 106)
- Publication type (n = 1)
- Study design (n = 278)
- Timing of exposure and outcome (n = 2)

Studies included in review

(n = 45)

**Identification of studies via databases and registers**

**Identification**

**Screening**

**Included**

### **Figure S4.** Funnel plots for studies reporting effect estimates for positive parenting (2A; k [number of studies]= 17; ES [number of effect sizes] = 35) and those reporting effect estimates for negative parenting (2B; k = 38; ES = 122). The different colours represent different cohorts.


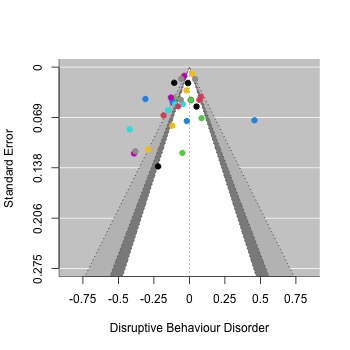

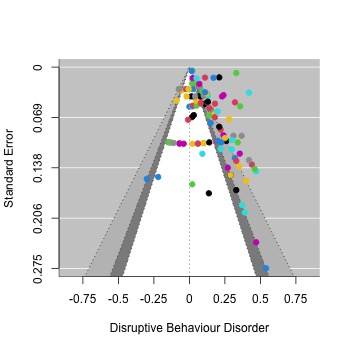


**A**

**B**

### **
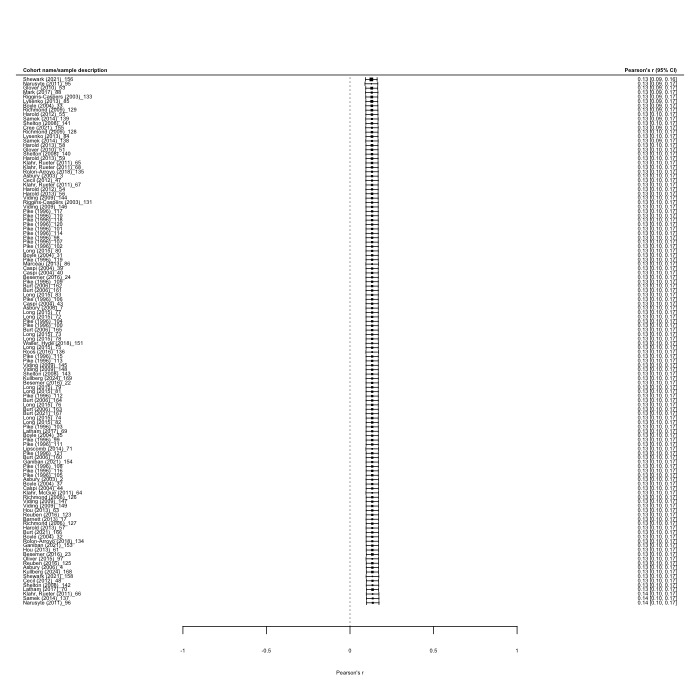

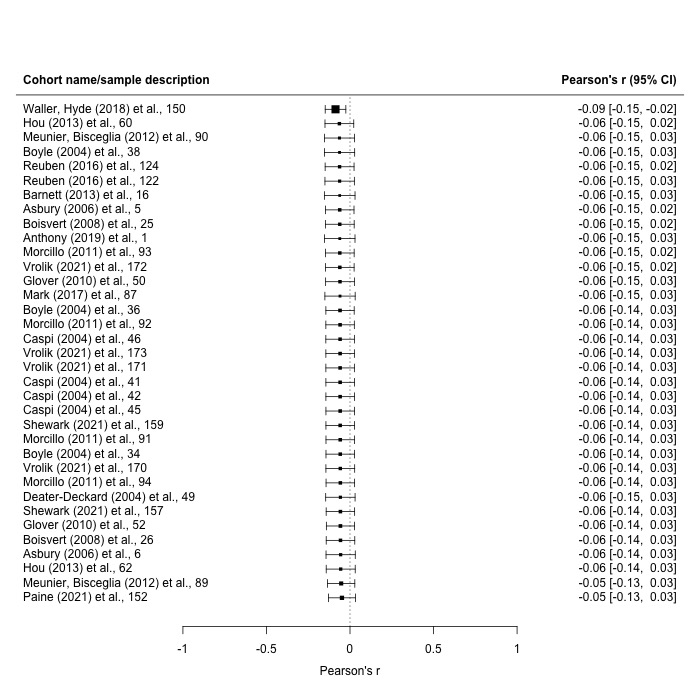
Figure S5.** Leave-one-out sensitivity analyses assessing whether any effect size had undue influence on the meta-analytic results for positive parenting (left) or negative parenting (right)

### **
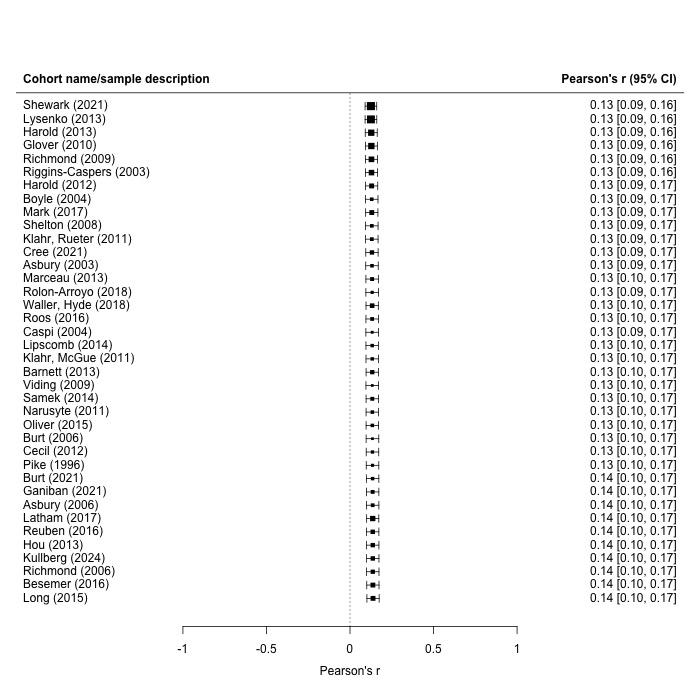

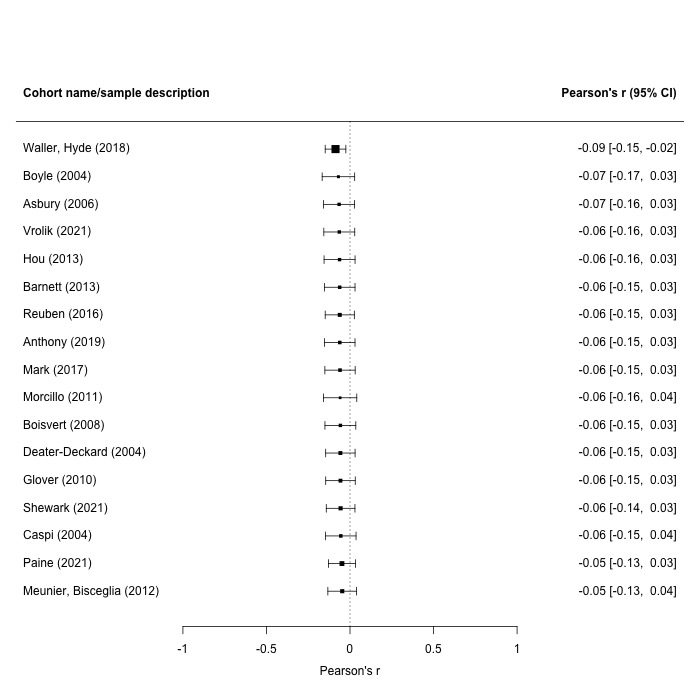
Figure S6.** Leave-one-out sensitivity analyses assessing whether any study had undue influence on the meta-analytic results for positive parenting (left) or negative parenting (right)

### **
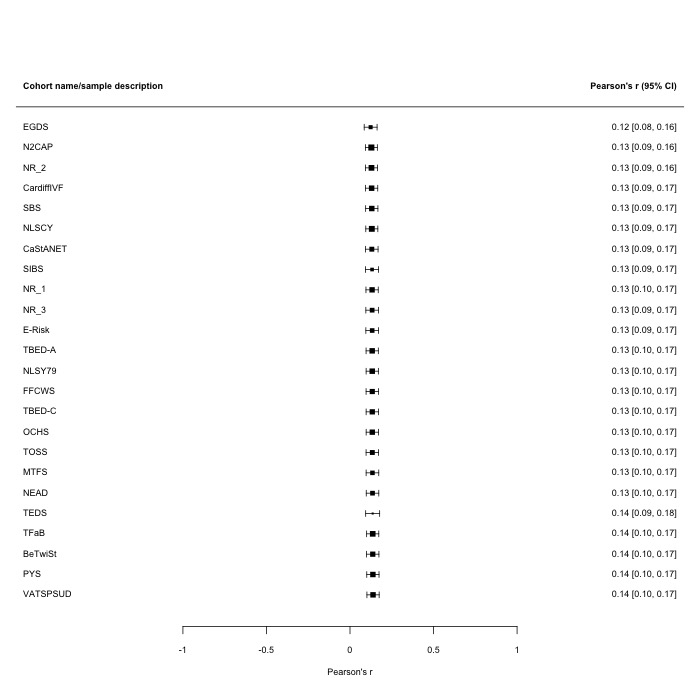

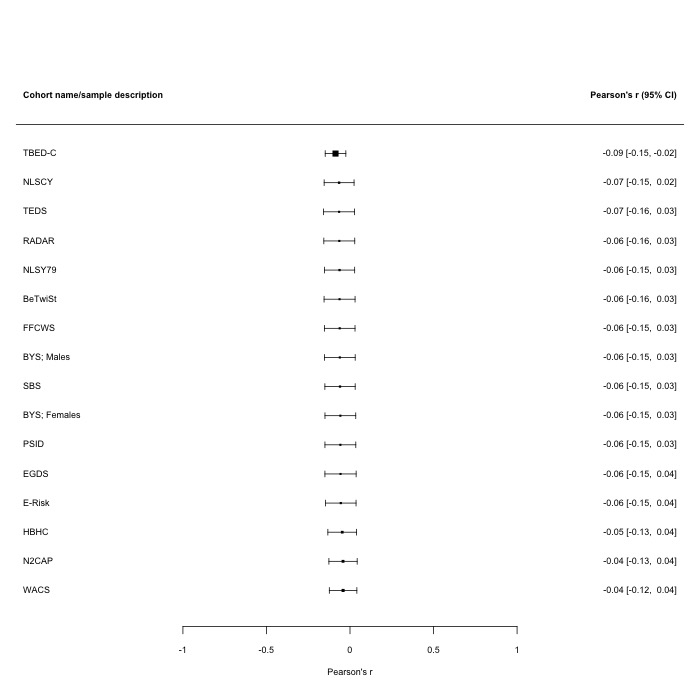
Figure S7.** Leave-one-out sensitivity analyses assessing whether any cohort had undue influence on the meta-analytic results for positive parenting (left) or negative parenting (right).

### **Figure S8.** Funnel plots for studies reporting effect estimates for negative parenting practices in studies categorised as very high-risk (S4A; k [number of studies] =11 ; ES [number of effect sizes] = 52), high-risk (S4B; k = 5 ; = 18) and high-quality (S4C, k = 22; ES =50 ). The different colours represent different cohorts.


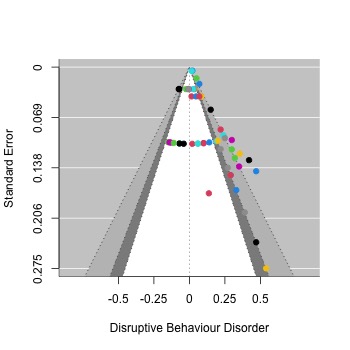

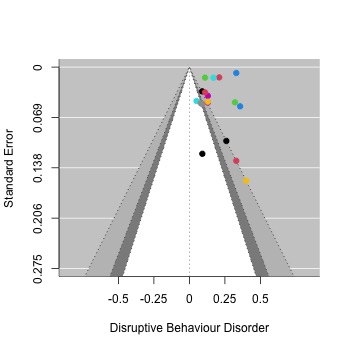

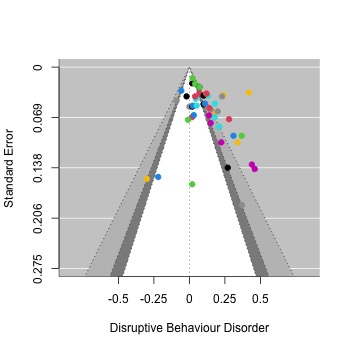


**A**

**B**

**C**

### **
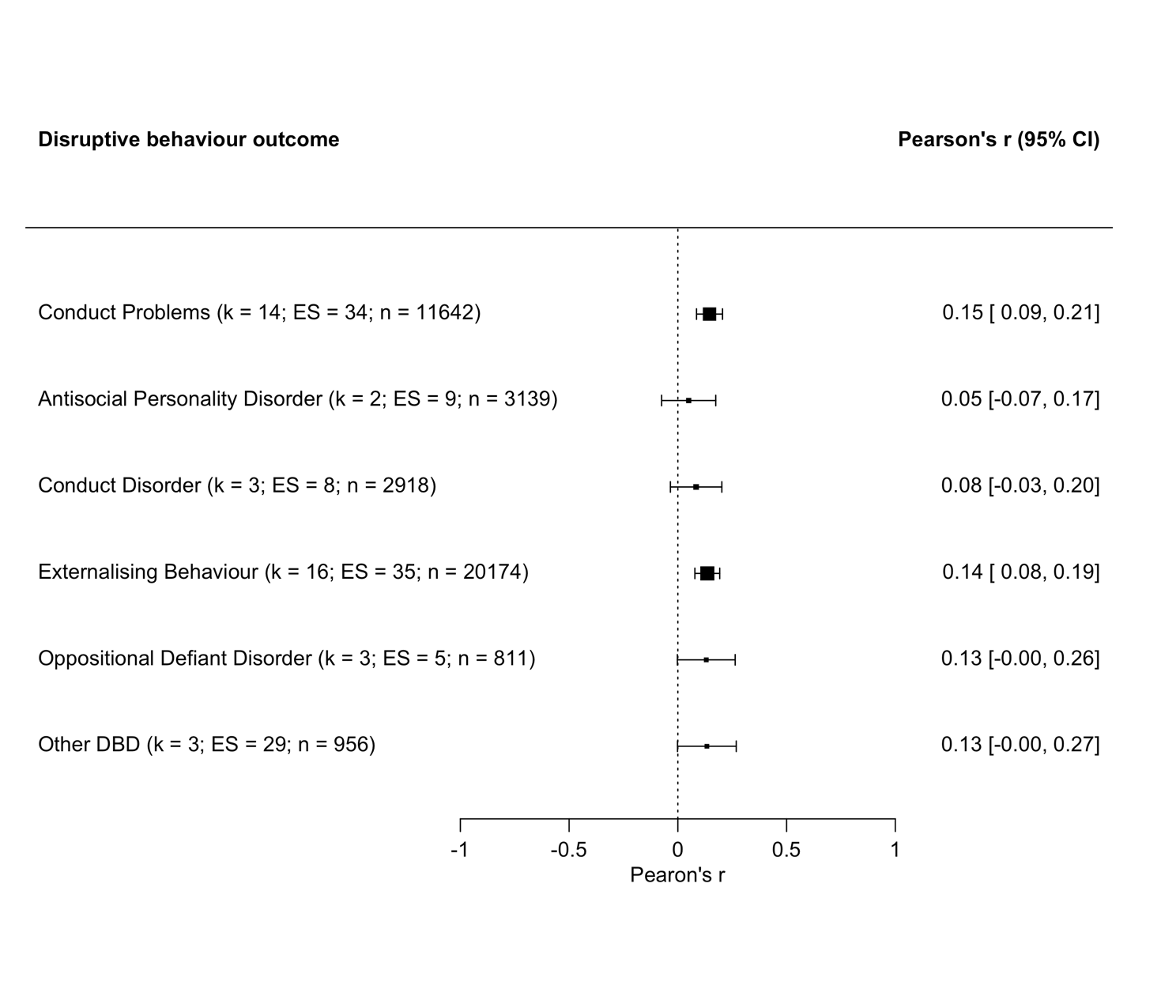
Figure S9.** Forest plot of the disruptive behaviour disorder outcome subgroup analyses.

### **Figure S10.** Forest plot of the quasi-experimental study type subgroup analyses.


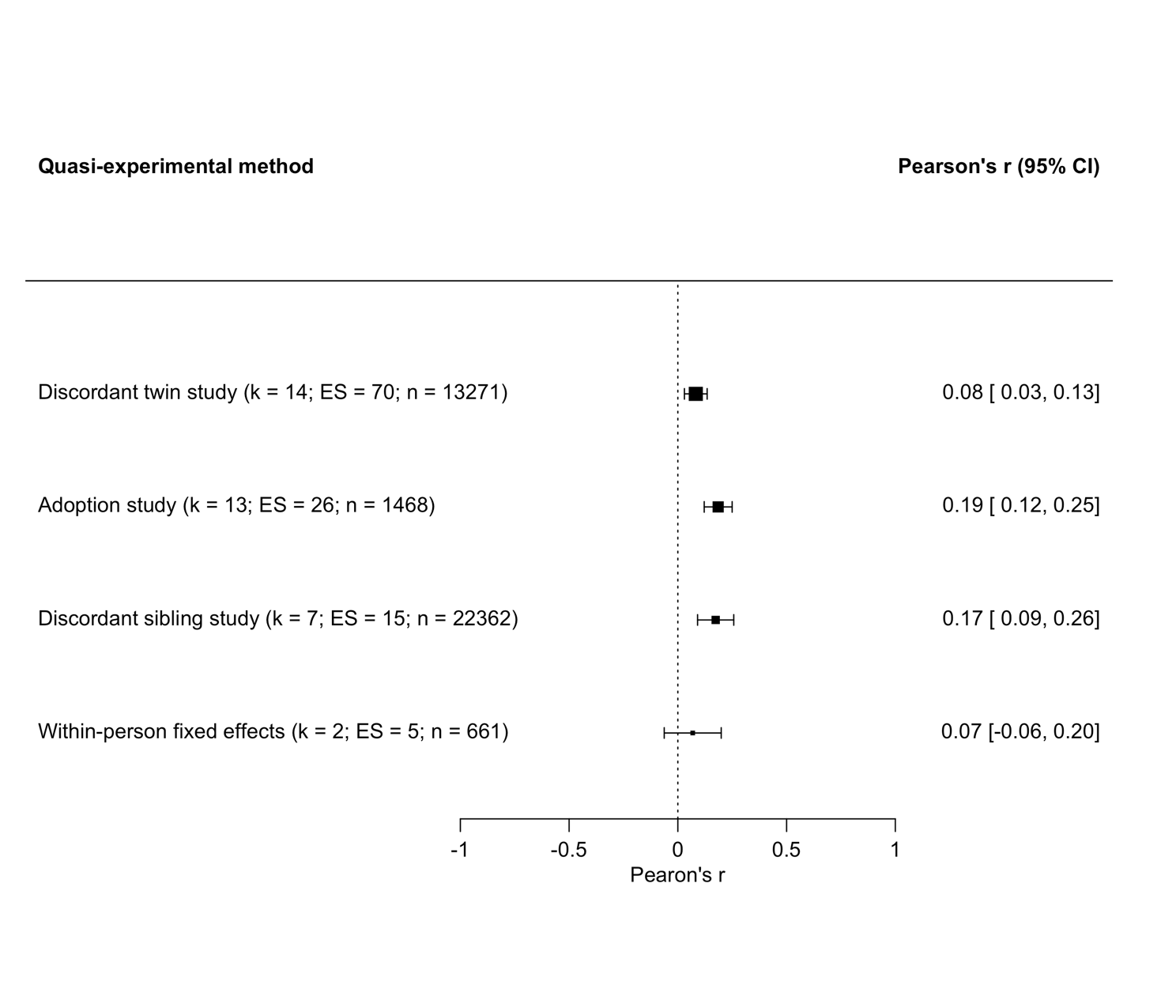


### **Figure S11.** Forest plot of the informant type subgroup analyses.


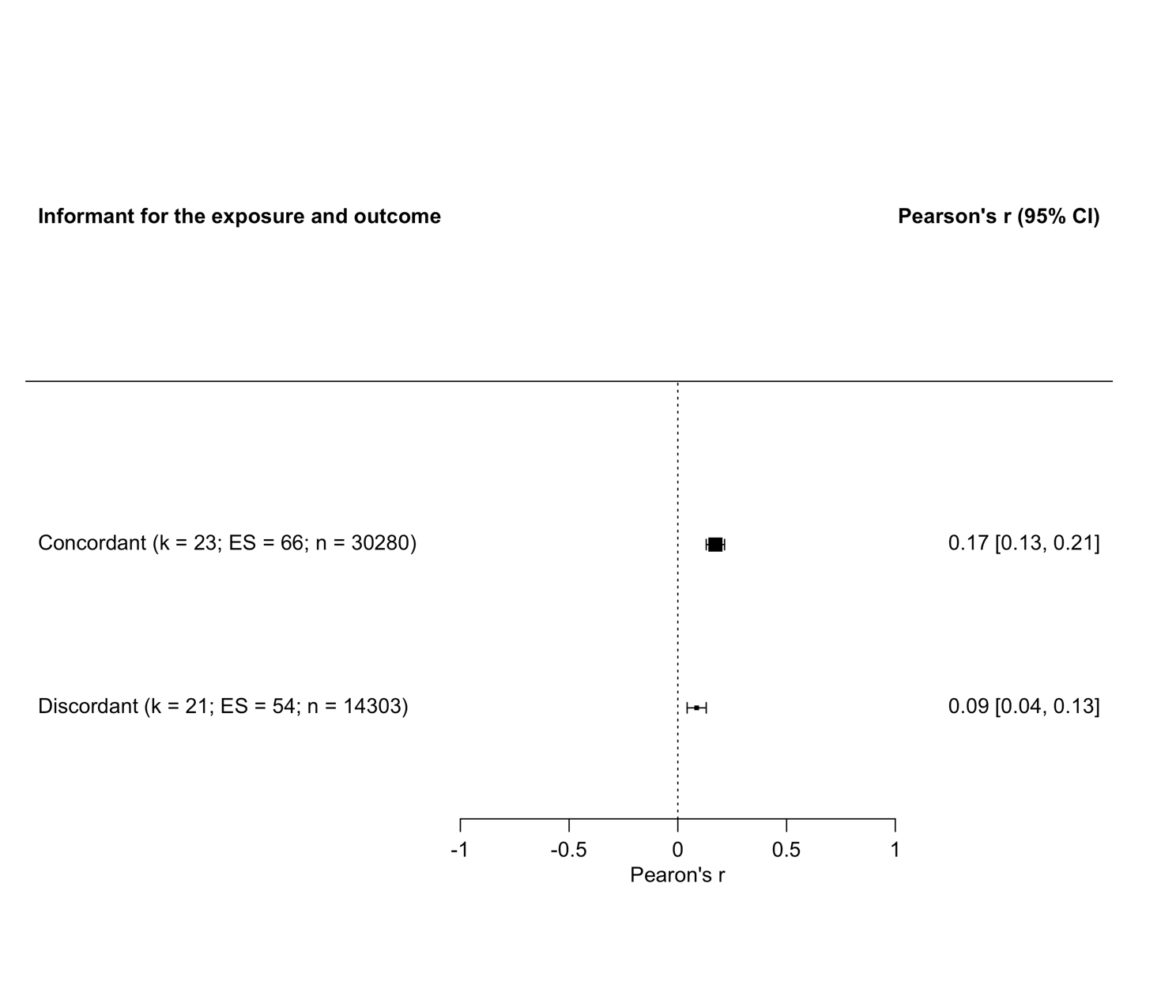


### **Figure S12.** Forest plot of the study quality subgroup analyses.


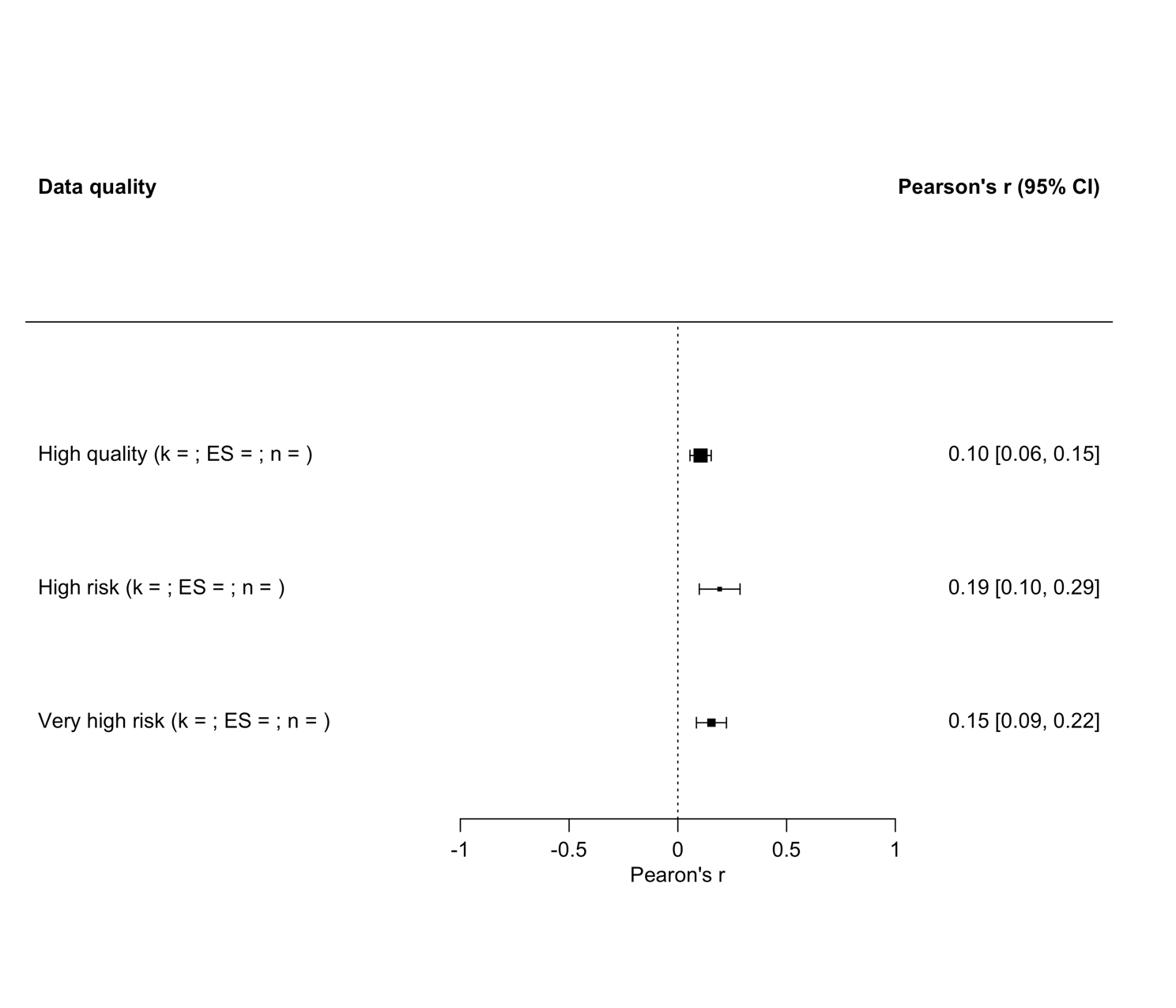


### **Figure S13.** Forest plot of the maternal versus paternal parenting practices subgroup analyses.


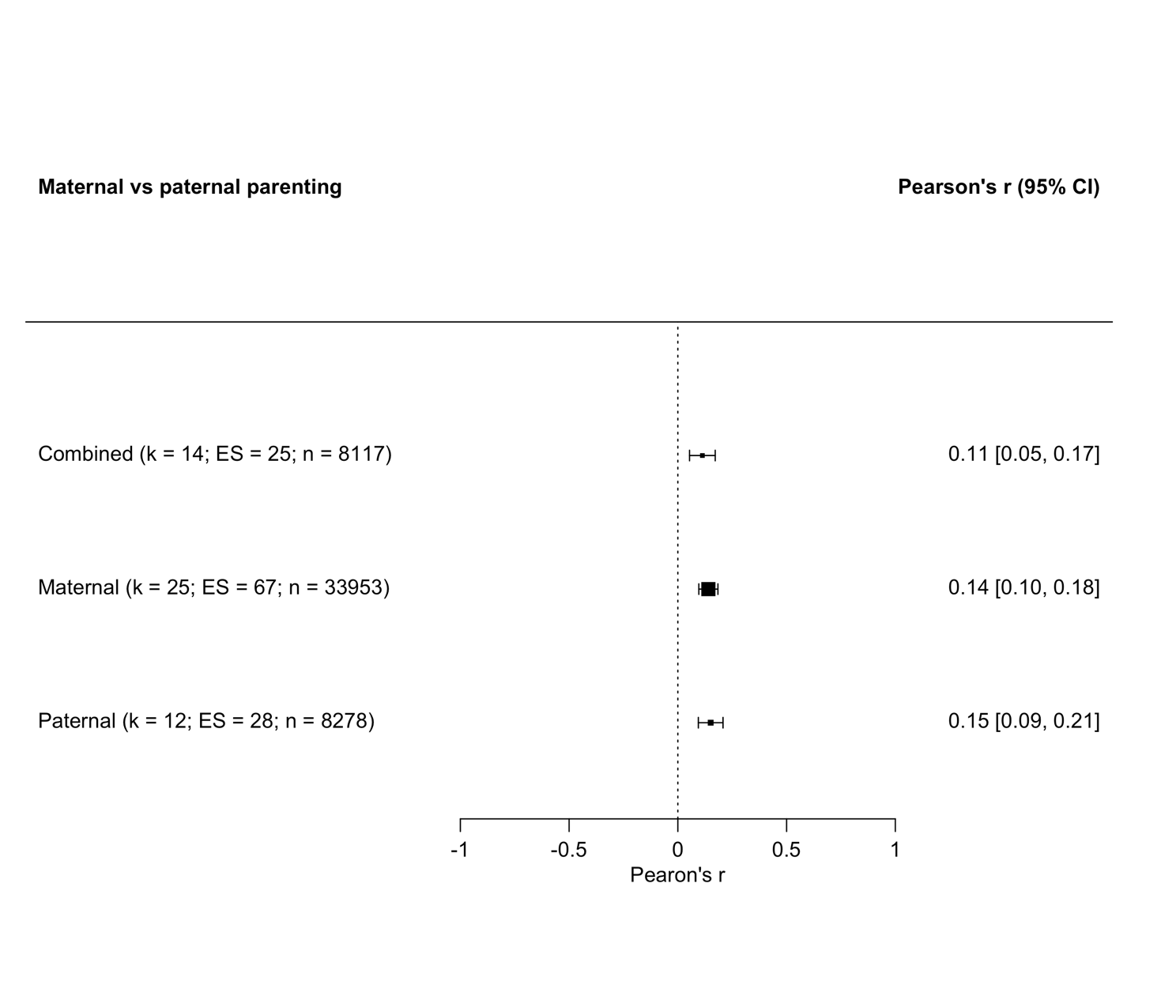


**References**

1 Pingault J-B, O’Reilly PF, Schoeler T, *et al.* Using genetic data to strengthen causal inference in observational research. *Nat Rev Genet* 2018;**19**:566–80. doi:10.1038/s41576-018-0020-3

2 Rutter M. ‘Natural Experiments’ as a Means of Testing Causal Inferences. In: *Causality: Wiley Series in Probability and Statistics*. 2012. 253–72. doi:10.1002/9781119945710.ch18

3 Gunasekara FI, Richardson K, Carter K, *et al.* Fixed effects analysis of repeated measures data. *Int J Epidemiol* 2014;**43**:264–9. doi:10.1093/ije/dyt221

4 Bor J, Moscoe E, Mutevedzi P, *et al.* Regression Discontinuity Designs in Epidemiology. *Epidemiology* 2014;**25**:729–37. doi:10.1097/EDE.0000000000000138

5 Davies NM, Holmes M V., Davey Smith G. Reading Mendelian randomisation studies: a guide, glossary, and checklist for clinicians. *BMJ* 2018;:k601. doi:10.1136/bmj.k601

6 Jandoc R, Burden AM, Mamdani M, *et al.* Interrupted time series analysis in drug utilization research is increasing: systematic review and recommendations. *J Clin Epidemiol* 2015;**68**:950–6. doi:10.1016/j.jclinepi.2014.12.018

7 Bärnighausen T, Oldenburg C, Tugwell P, *et al.* Quasi-experimental study designs series—paper 7: assessing the assumptions. *J Clin Epidemiol* 2017;**89**:53–66. doi:10.1016/j.jclinepi.2017.02.017

8 Hernan MA. A definition of causal effect for epidemiological research. *J Epidemiol Community Heal* 2004;**58**:265–71. doi:10.1136/jech.2002.006361

9 Rubin DB. For objective causal inference, design trumps analysis. *Ann Appl Stat* 2008;**2**:808–40. doi:10.1214/08-AOAS187

10 Rubin DB. The designversus the analysis of observational studies for causal effects: parallels with the design of randomized trials. *Stat Med* 2007;**26**:20–36. doi:10.1002/sim.2739

11 Funk MJ, Westreich D, Wiesen C, *et al.* Doubly Robust Estimation of Causal Effects. *Am J Epidemiol* 2011;**173**:761–7. doi:10.1093/aje/kwq439

12 National Institute for Health and Care Excellence. Antisocial behaviour and conduct disorders in children and young people: recognition and management. 2013. http://guidance.nice.org.uk/CG158
